# Exchange-mediated spin–electric control of single molecules on surfaces

**DOI:** 10.1038/s41567-026-03353-w

**Published:** 2026-06-29

**Authors:** Paul Greule, Wantong Huang, Máté Stark, Kwan Ho Au-Yeung, Johannes Schwenk, Jose Reina-Gálvez, Christoph Sürgers, Wolfgang Wernsdorfer, Christoph Wolf, Philip Willke

**Affiliations:** 1 Physikalisches Institut, Karlsruhe Institute of Technology, Karlsruhe, Germany; 2 Center for Integrated Quantum Science and Technology, Karlsruhe Institute of Technology, Karlsruhe, Germany; 3 Department of Physics, University of Konstanz, Konstanz, Germany; 4 Institute for Quantum Materials and Technologies, Karlsruhe, Germany; 5 Center for Quantum Nanoscience, Institute for Basic Science, Seoul, Republic of Korea; 6 Ewha Womans University, Seoul, Republic of Korea

**Keywords:** Organic-inorganic nanostructures, Quantum information

## Abstract

Individual magnetic molecules are promising building blocks for quantum technologies owing to their chemical tunability, nanoscale dimensions and ability to self-assemble into ordered arrays. However, exploiting their properties in quantum information processing requires precise local control of their spin. Here we demonstrate spin–electric coupling for two molecular spin systems—iron phthalocyanine (FePc) and Fe–FePc complexes—adsorbed on a surface. We use electron spin resonance combined with scanning tunnelling microscopy to locally address them and electrically tune them using an applied bias voltage. These measurements reveal a nonlinear voltage dependence of the resonance frequency, linked to the energetic position of the molecular orbitals. We attribute this effect to a transport-mediated exchange field from the magnetic tip, providing a large, highly localized and broadly applicable spin–electric coupling mechanism. Finally, we demonstrate that the spin–electric coupling enables all-electrical coherent spin control. In Rabi oscillation measurements of both single and coupled Fe–FePc complexes, we show that the spin dynamics can be tuned using the exchange field, demonstrating a pathway towards electrically controlled quantum operations.

## Abstract (web summary)

Single molecules that host isolated spins are promising candidates for applications in quantum technologies. Now it is shown that exchange interactions enable electric control of spins in two different molecular species.

## Main

Electron and nuclear spins in single molecules have attracted substantial interest as potential building blocks for applications in spintronics, quantum sensing, and quantum computing^1^. Magnetic molecular systems are of nanoscopic size, benefit from self-assembly and offer unique structural as well as chemical tunability via modern synthetic chemistry^2,3^. A key challenge lies in achieving reliable and local control over individual spin centres. A potential solution is the use of electric fields, which—in contrast to magnetic fields—can be efficiently applied in a confined region^4^. Thus, spin–electric coupling (SEC) in molecules has in recent years emerged as a promising control mechanism, which has been discussed theoretically^5–7^ and realized experimentally^8–16^ in a variety of systems. These specifically tailored molecular platforms typically rely on structural distortions that modulate key parameters of the spin Hamiltonian, such as zero-field splitting, g-factor, orbital angular momentum, hyperfine interaction or exchange coupling. Often, effective SEC requires a soft, electrically polarizable molecular environment and spin energy levels that are highly sensitive to structural changes. However, the experimentally observed shifts Δf in spin resonance frequencies due to electric fields have so far remained relatively modest, amounting to less than $\frac{\Delta f}{{f}_{0}}$ < 1% of the qubit’s unperturbed resonance frequency *f*_0 _= *gμ*_B_*B*/*h*. This limitation arises partly because most SEC mechanisms across platforms are found to be linear^17^, and exceptions of higher-order contributions have been rarely observed and discussed^6,18–20^.

Recently, SEC has been studied using electron spin resonance scanning tunnelling microscopy (ESR–STM): This combination of ESR with STM has emerged as a powerful tool providing sub-ångström resolution while also permitting coherent control of single spin states^21–24^. For SEC, shifts of up to $\frac{\Delta f}{{f}_{0}}$ ≈ 3% have been reported for individual atomic and molecular spins dominated by a linear response^25,26^. Different theoretical models have been proposed to account for SEC in an STM junction, including piezoelectric distortion^27,28^ and transport-mediated exchange interaction^29–31^. While the latter promises a nonlinear, universal and highly localized mechanism for tuning atomic-scale quantum systems^29,30^, neither this predicted behaviour nor its application in coherent control has been demonstrated experimentally.

In this study, we use ESR–STM to investigate the SEC in two molecular spin systems on MgO/Ag(001)—iron phthalocyanine (FePc) and an Fe–FePc complex. We show that varying the d.c. bias voltage in the STM junction shifts the resonance frequency of the spin transition ${f}_{{\rm{res}}}\propto {V}_{{\rm{DC}}}$. In FePc, this shift becomes strongly nonlinear—reaching close to 30%—when the electrochemical potential is close to the lowest unoccupied molecular orbital (LUMO). We attribute this behaviour to a transport-mediated exchange interaction between the magnetic tip and the molecule spin^26,29–31^, which predicts such a logarithmic divergence. This effect is not only large and highly localized (even more so than electric fields), but also universal and consequently broadly applicable to other spin systems such as quantum dots and solid-state spin defects. In the second part, we demonstrate that the SEC can be readily employed in all-electrical coherent control of spin dynamics: Rabi oscillation measurements on Fe–FePc complexes reveal that individual spins can be selectively detuned using ${V}_{{\rm{DC}}}$ only. Finally, we extend this to electric tuning of coupled dimer spins, highlighting the potential of SEC for individual control with nanoscale precision in larger spin assemblies.

## Results and discussion

### SEC of molecules on MgO/Ag(001)

**Fig. 1:**
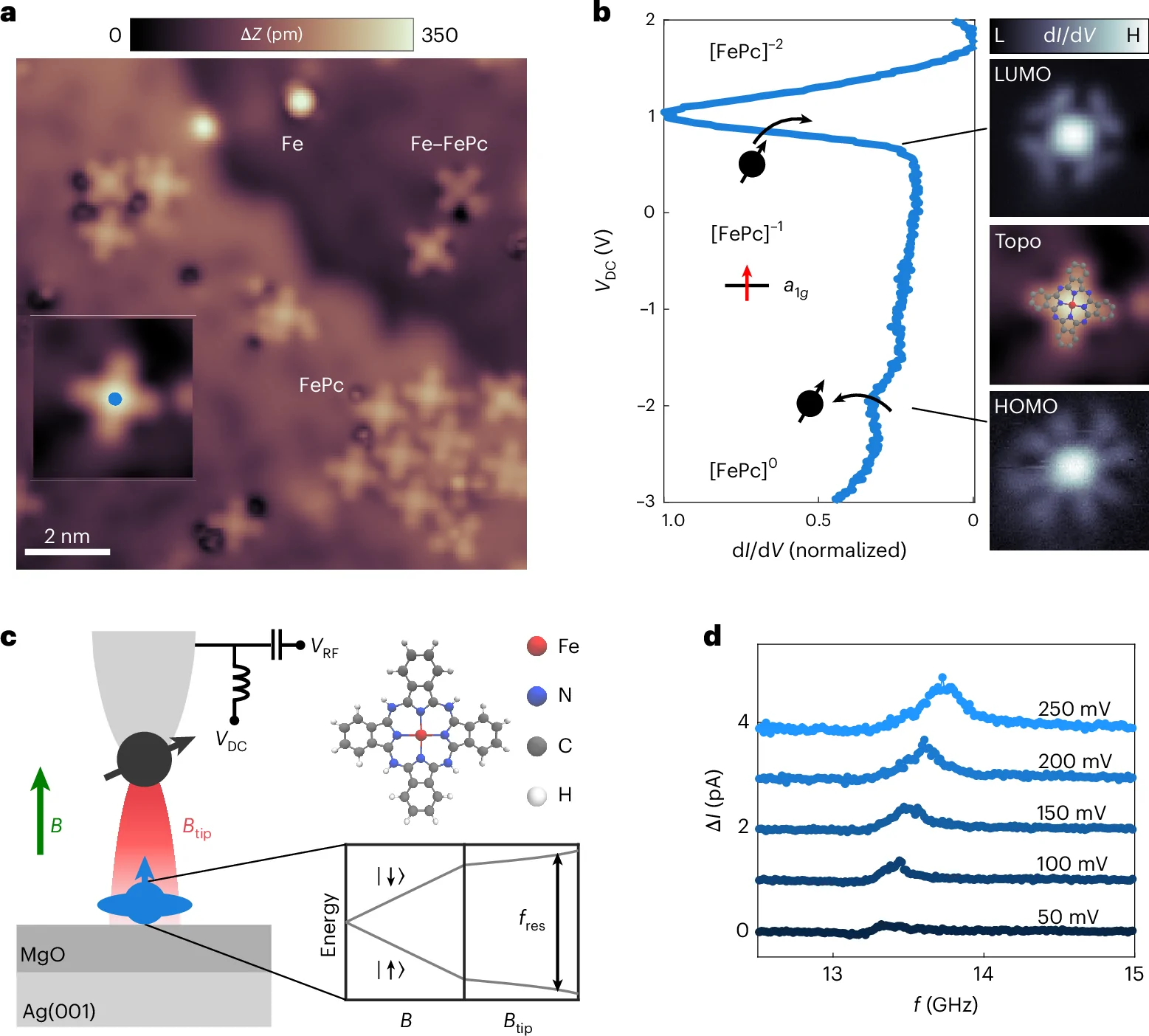
Molecular spins on MgO/Ag(001). **a**, STM topography of the surface with the deposited Fe atoms and FePc molecules and a built Fe–FePc complex (image conditions: $I=20\,{\rm{pA}},{V}_{{\rm{DC}}}=-100\,{\rm{mV}}$). The inset shows a close-up topography of a single FePc molecule (2.2 nm × 2.2 nm, *I* = 50 pA, *V*_DC_ = 100 mV). The blue dot marks the tip position of the experiments shown in **b** and **d**. **b**, Left: differential conductance (d*I*/d*V*) spectra acquired on the centre of the FePc (${I}_{{\rm{set}}}=30\,{\rm{pA}},\,{V}_{{\rm{set}}}=2\,{\rm{V}},\,{V}_{\mathrm{mod}}=10\,{\rm{mV}}$). The arrows indicate the removal (addition) of an electron leading to the transition from the [FePc]^−1^ (with an unpaired spin in the molecule’s *a*_1*g*_ orbital) to the [FePc]^0^ and [FePc]^−2^ charge state. Right: the d*I*/d*V* maps show the spatial extent of both states (−2,000 mV and +400 mV) alongside the topography with an inserted chemical structure drawing. **c**, Left: schematic drawing of the experimental set-up with a spin-polarized STM tip above the FePc molecule atop MgO/Ag(001). A d.c. bias voltage ${V}_{{\rm{DC}}}$ and RF voltage ${V}_{{\rm{RF}}}$ are applied across the tunnel junction. The magnetic tip, realized by picking up individual Fe atoms from the surface, creates a highly localized tip field ${B}_{{\rm{tip}}}$ acting on the surface spin together with an externally applied magnetic field B. Right: This is illustrated by the energy level diagram of the spin ½. Top right: chemical configuration of the FePc molecule. **d**, Electron spin resonance (ESR) measured on an FePc molecule for different ${V}_{{\rm{DC}}}$ showing the change in tunnel current ΔI as a function of frequency f (ESR conditions: ${I}_{{\rm{set}}}=20\,{\rm{pA}},\,{V}_{{\rm{set}}}=60\,{\rm{mV}},{B}=484\,{\rm{mT}},\,{V}_{{\rm{RF}}}=10\,{\rm{mV}}$). The frequency sweeps were taken at constant height (open feedback loop) and are vertically shifted for clarity.

An STM topography of the sample is shown in Fig. 1a. We deposited Fe atoms and FePc molecules onto two monolayers of MgO atop an Ag(001) crystal. FePc molecules were shown to form an *S* = 1/2 system that is localized on the central Fe atom^32^. In addition, we include in this work a spin complex that consists of one FePc molecule that is strongly coupled to an adjacent Fe atom via one of its ligands. As shown previously^33^, these Fe–FePc organometallic complexes can be built using tip-assisted assembly and form a mixed-spin (1,1/2) ferrimagnet with a well-separated doublet of ${m}_{{\rm{z}}}=\pm \frac{1}{2}$, mimicking an *S* = 1/2 system. Both FePc and Fe–FePc constitute ideal two-level systems that allow coherent quantum control^22,33^. We employ both in this study, as the molecular orbital structure of FePc best demonstrates the exchange bias mechanism, while coherent control is facilitated in the Fe–FePc complex due to its resilience to inelastic electron scattering^33^.

**Extended Data Fig. 1:**
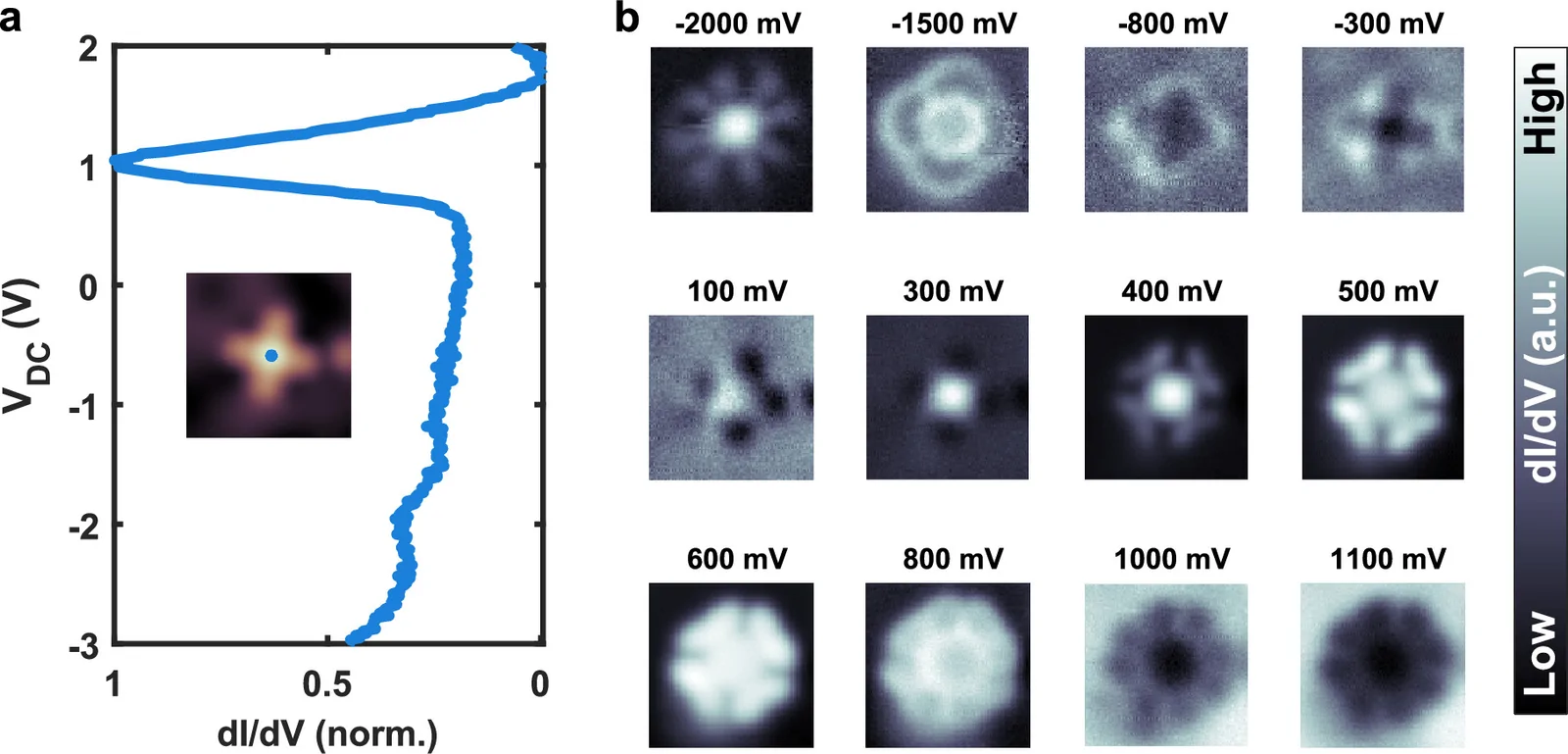
Differential conductance maps of FePc. **a**, Normalized differential conductance (d*I*/d*V*) spectra acquired on an isolated FePc molecule (blue) (${I}_{{\rm{set}}}=3\,{\rm{pA}},\,{V}_{{\rm{set}}}=2000\,{\rm{mV}},\,{V}_{\mathrm{mod}}=10\,{\rm{mV}}$). The inset shows the measurement position as a colored dot on the topography. **b**, Spatially resolved d*I*/d*V* maps of the single FePc molecule shown in **a**. The signal is recorded in closed loop at the bias voltages ${V}_{{\rm{DC}}}$ via a lock-in modulation ${V}_{\mathrm{mod}}$ ($2.2\,{\rm{nm}}\times 2.2\,{\rm{nm}}$, ${I}_{{\rm{set}}}=50\,{\rm{pA}},\,{V}_{\mathrm{mod}}=10\,{\rm{mV}}$).

**Extended Data Fig. 2:**
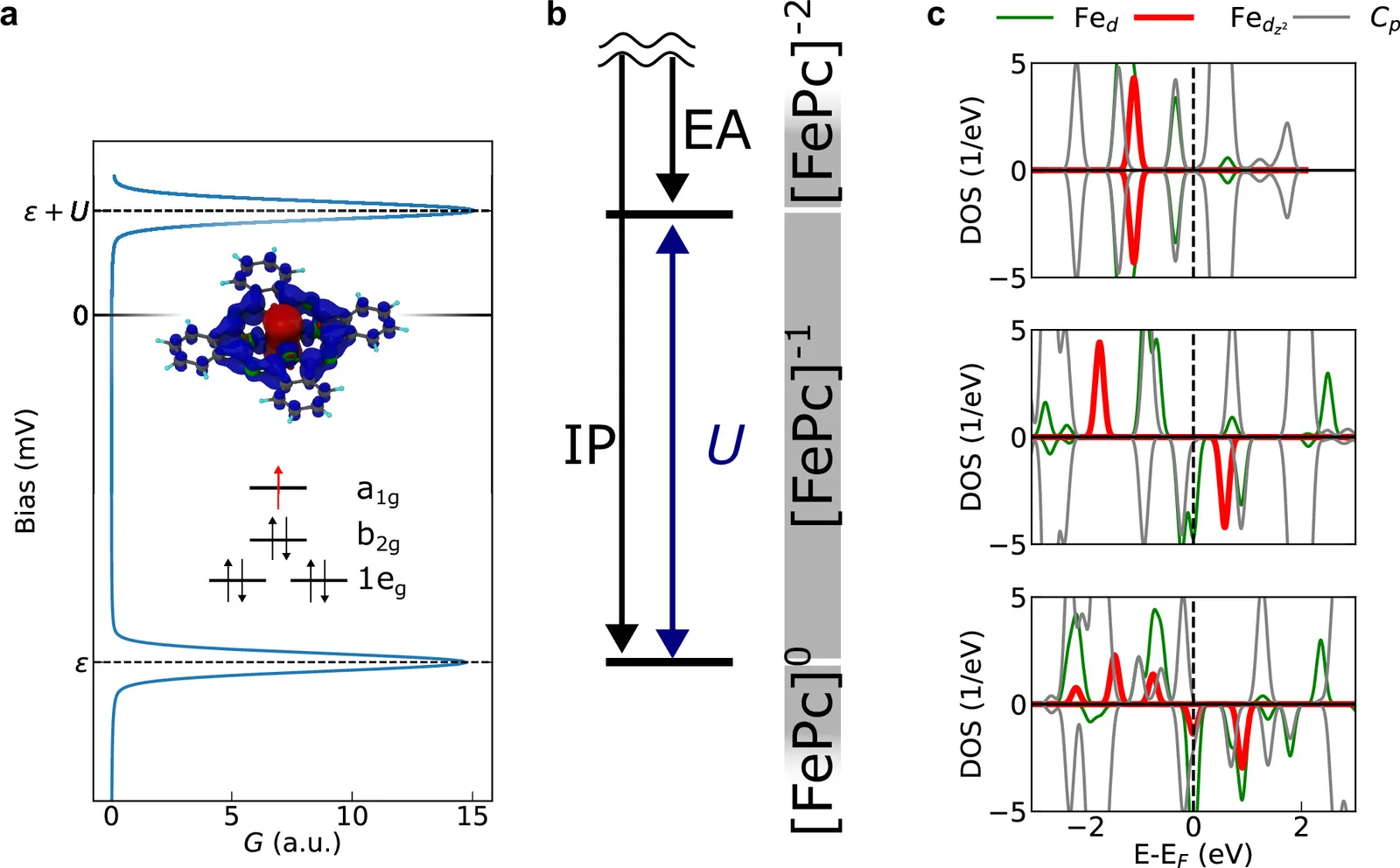
DFT calculations of FePc. **a**, Schematic representation of the spectral function for a single impurity Anderson model (SIAM) with the two relevant charge transitions at ionization energy ε and charging energy ε+U. The inset shows the energy level diagram of the [FePc]^−1^ charge state with the single occupied a_1g_ orbital which has a strong dz^2^ contribution (71%). The spin polarization iso-surface above demonstrates the strong dz^2^ character of the orbital. **b**, Correspondence to the DFT ΔSCF calculations, indicating ionization potential (IP) and electron affinity (EA); their difference is U. **c**, Calculated spin-polarized projected DOS (PDOS) for the three charge configurations: [FePc]^−2^, [FePc]^−1^ and [FePc]^0^.

The electronic structure of the FePc molecule is characterized in Fig. 1b by differential conductance d*I*/d*V* measurements (see also ref. ^32^). We observe pronounced conductance peaks around −2,000 mV (+1,000 mV) related to the process of removing (adding) an electron to the molecule^34^. We assign these energy positions to the highest occupied molecular orbital (HOMO) and the LUMO^32,35^, respectively (see also Extended Data Fig. 1 and Supplementary Section 3). Density functional theory (DFT) calculations indicate that the FePc electronic configuration involves one unpaired spin in the *a*_1*g*_ orbital. This results from a charge transfer from the substrate ([FePc]^−1^) and leads to an *S* = 1/2 ground state^22,29,32^ (see also Extended Data Fig. 2 and Supplementary Section 4). The resulting magnetic spin state can be probed by ESR–STM (Fig. 1c). For ESR, the ${m}_{{\rm{z}}}=\pm \frac{1}{2}$ ground states are split by an external magnetic field *B* perpendicular to the sample surface

1 $${{hf}}_{\mathrm{res}}=g{\mu }_{{\rm{B}}}\left(B+{B}_{\mathrm{tip}}\right),$$

where ${f}_{{\rm{res}}}$ is the resonance frequency, h is Planck’s constant, ${\mu }_{{\rm{B}}}$ is the Bohr magneton and g is the *g*-factor. For both spin systems, g was found to be approximately 2 (refs. ^32,33^). Moreover, ${B}_{{\rm{tip}}}$ accounts for the influence of the highly localized magnetic tip field leading to a shift $\Delta f={f}_{{\rm{res}}}-{f}_{0}$ of the surface spin’s resonance frequency. ${B}_{{\rm{tip}}}$ consists of both magnetic exchange and magnetic dipole–dipole interaction^36,37^, which results in different amplitude and sign of ${B}_{{\rm{tip}}}$ depending on the particular magnetic tip apex.

**Extended Data Fig. 3:**
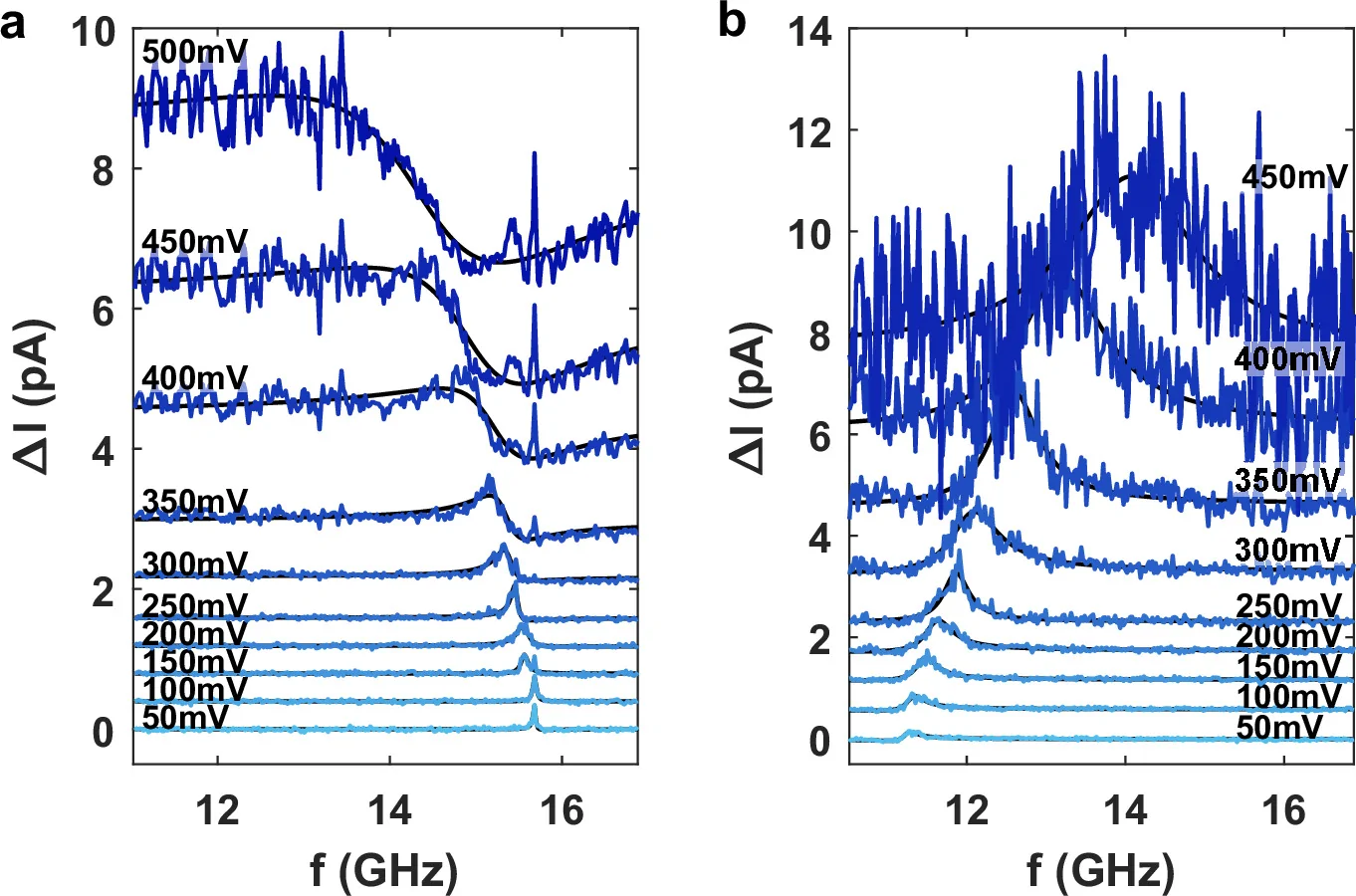
Voltage dependent frequency sweeps on FePc. **a**, Waterfall plot of individual frequency sweeps from the experimental data presented in Fig. 2a showing the change in tunneling current ΔI as a function of frequency f for various bias voltages ${V}_{{\rm{DC}}}$. The black solid lines represent fits to the Fano function shown in Eq. (S1). The traces are vertically offset for clarity. **b**, Analogous to **a** showing frequency sweeps from the experimental data presented in Fig. 2b.

Motivated by SEC, which was recently observed for individual Ti atoms on MgO/Ag(001)^25^ in ESR–STM, we investigated the dependence of the ESR signal as a function of bias voltage ${V}_{{\rm{DC}}}$ (Fig. 1d). Here, we keep the tip–sample distance and the external field B constant while sweeping the ESR frequency. Indeed, we find a linear dependence ${f}_{{\rm{res}}}\propto {V}_{{\rm{DC}}}$ for the voltage range shown. This frequency shift can be interpreted as a contribution to the Zeeman energy by the SEC (see sketch in Fig. 1c). The intensity of the ESR signal increases with$\,\left|{V}_{{\rm{DC}}}\right|$, mainly due to increasing tunnelling current *I* (Extended Data Fig. 3). For different magnetic tips, we observe that the linear voltage dependence occurs with a varying magnitude ranging from 0.5 to 8 MHz mV^−1^ (Supplementary Section 7). In the case of Ti atoms, a linear shift of similar magnitude was explained by a piezoelectric coupling between the magnetic tip and the surface spin^25^: as a consequence of ${V}_{{\rm{DC}}}$, the spin is displaced in the magnetic field of the tip, which increases or decreases ${B}_{{\rm{tip}}}$. This effect is additionally accompanied by a change in the *g*-factor. By contrast, in recent works by some of the authors, it was theoretically proposed that SEC can also result from transport-mediated exchange interaction^29–31^.

### Nonlinear SEC

**Fig. 2:**
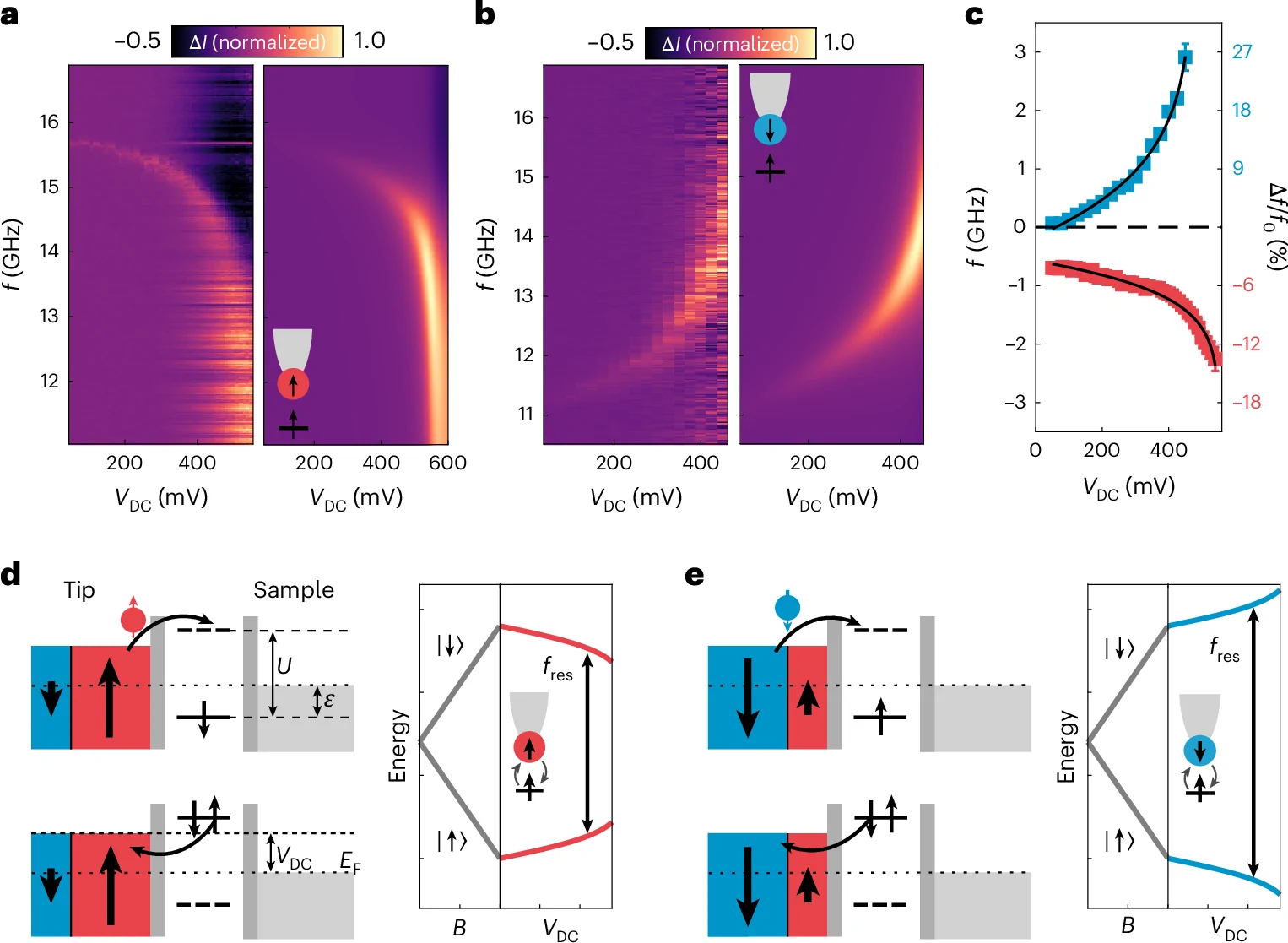
Nonlinear SEC in the ESR spectra on FePc. **a**, Colour map of the ESR signal ΔI as a function of ${V}_{{\rm{DC}}}$ and f on a single FePc molecule. Left: experimental data (ESR conditions: ${I}_{{\rm{set}}}=10\,{\rm{pA}},\,{V}_{{\rm{set}}}=60\,{\rm{mV}},\,B=585\,{\rm{mT}},\,{V}_{{\rm{RF}}}=8\,{\rm{mV}}$). Sharp horizontal lines are rectifications of the RF transfer function. Right: simulation according to the exchange bias model. The inset illustrates an STM tip with a spin polarization P>0 used for the simulation. **b**, ESR map $\Delta I(f,\,{V}_{{\rm{DC}}})$ analogous to **a**, but with a different magnetic tip with P<0 (${I}_{{\rm{set}}}=20\,{\rm{pA}},\,{V}_{{\rm{set}}}=60\,{\rm{mV}},\,B=399\,{\rm{mT}},\,{V}_{{\rm{RF}}}=8\,{\rm{mV}}$). **c**, Frequency shift $\Delta f={f}_{{\rm{res}}}-2{\mu }_{{\rm{B}}}B/h$ over ${V}_{{\rm{DC}}}$ extracted from the spectra in **a** (red) and **b** (blue). The black lines show corresponding fits of the exchange bias model, see equations (1) and (2). The second *y-*axis on the right-hand side displays the relative change of the resonance frequency $\Delta f/{f}_{0}$ with ${f}_{0}=16.38\,{\rm{GHz}}\,(11.17\,{\rm{GHz}})$ for the red (blue) dataset. **d**,**e**, Schematic drawings of the virtual tunnelling processes leading to the tip-induced exchange field: The molecular spin is described via a single-impurity Anderson model (SIAM)^45,46^. The molecular energy levels, described by the ionization energy ϵ and the Coulomb repulsion energy U, lie between the electrochemical potential of the left spin-polarized tip electrode and the right sample electrode, separated by the vacuum tunnelling barrier and the MgO layer, respectively. The bias voltage ${V}_{{\rm{DC}}}$ moves the potential of the magnetic tip closer to the doubly occupied level for positive voltages. The polarization of the tip determines the dominating virtual tunnelling (spin up in **d**, spin down in **e**), which favour different spin states of the molecule. This leads to different ${B}_{{\rm{tip}}}$, as depicted in the energy level diagrams.

To elucidate the mechanism, Fig. 2 shows the voltage dependence of the resonance frequency for FePc molecules over a wider bias voltage range than in Fig. 1d and for different magnetic tips. For the first magnetic tip in Fig. 2a, we find that the resonance peak position starts to shift drastically and in a nonlinear manner at voltages above ≈250 mV. This shift Δf is accompanied by an increase in peak linewidth and amplitude as well as a change in its asymmetry. In addition, we find that Δf changes sign when employing a tip of opposite magnetic field direction ${B}_{{\rm{tip}}}$ (Fig. 2b). In Fig. 2c, we compare the shift of the resonance frequency $\Delta f({V}_{{\rm{DC}}})$ for both datasets. Due to the nonlinearity, the relative shift $\frac{\Delta f}{{f}_{0}}$ in Fig. 2c is rather large $[\pm (10-30) \%$] compared with previous works (≈3% in ref. ^25^ and ≈0.2% in ref. ^9^). We stress that such nonlinear behaviour as found in Fig. 2 is not expected from a piezoelectric displacement model as previously used for Ti atoms^25^. Moreover, the latter relies on the electric-field-induced displacement of the charged surface spin, and we find this to be incompatible with the observed sign of Δf for FePc (Supplementary Section 6).

### Exchange-mediated SEC

In the following, we aim to explain the behaviour by the exchange interaction between the molecule and the magnetic tip, referred to as exchange bias^26,29,30,38–42^. Notably, the nonlinear part of Δf emerges when the applied ${V}_{{\rm{DC}}}$ reaches the onset of the FePc LUMO shown in Fig. 1b. This indicates that the SEC mechanism is influenced by the unoccupied electronic states. A similar effect is found in quantum dot spin systems^38–40^, for instance in carbon nanotubes contacted with ferromagnetic electrodes^38^. The concept of exchange bias relies on virtual tunnelling processes into the excited states and was recently described in the framework of ESR–STM^26,29–31^. Figure 2d,e shows a schematic of the exchange bias in the tunnelling junction for two magnetic tips with opposite polarizations. In both cases, increasing ${V}_{{\rm{DC}}}$ raises the electrochemical potential of the tip. Subsequently, the up (down) polarization of the spin-polarized tip enhances virtual tunnelling of spin up (down) electrons into the doubly occupied state. Due to the imbalance of the spin densities in the tip electrode, the virtual tunnelling processes cause different energy corrections for the spin up and down state, which adds to the Zeeman energy. This spin-dependent energy correction can be written as^38^

2 $${B}_{\mathrm{tip}}=-\frac{P{\gamma }_{{\rm{T}}}}{2{\rm{\pi }}}\mathrm{ln}\left(\left|\frac{\epsilon -e{V}_{\mathrm{DC}}}{\epsilon +U-e{V}_{\mathrm{DC}}}\right|\right)+{B}_{0}.$$

**Extended Data Fig. 4:**
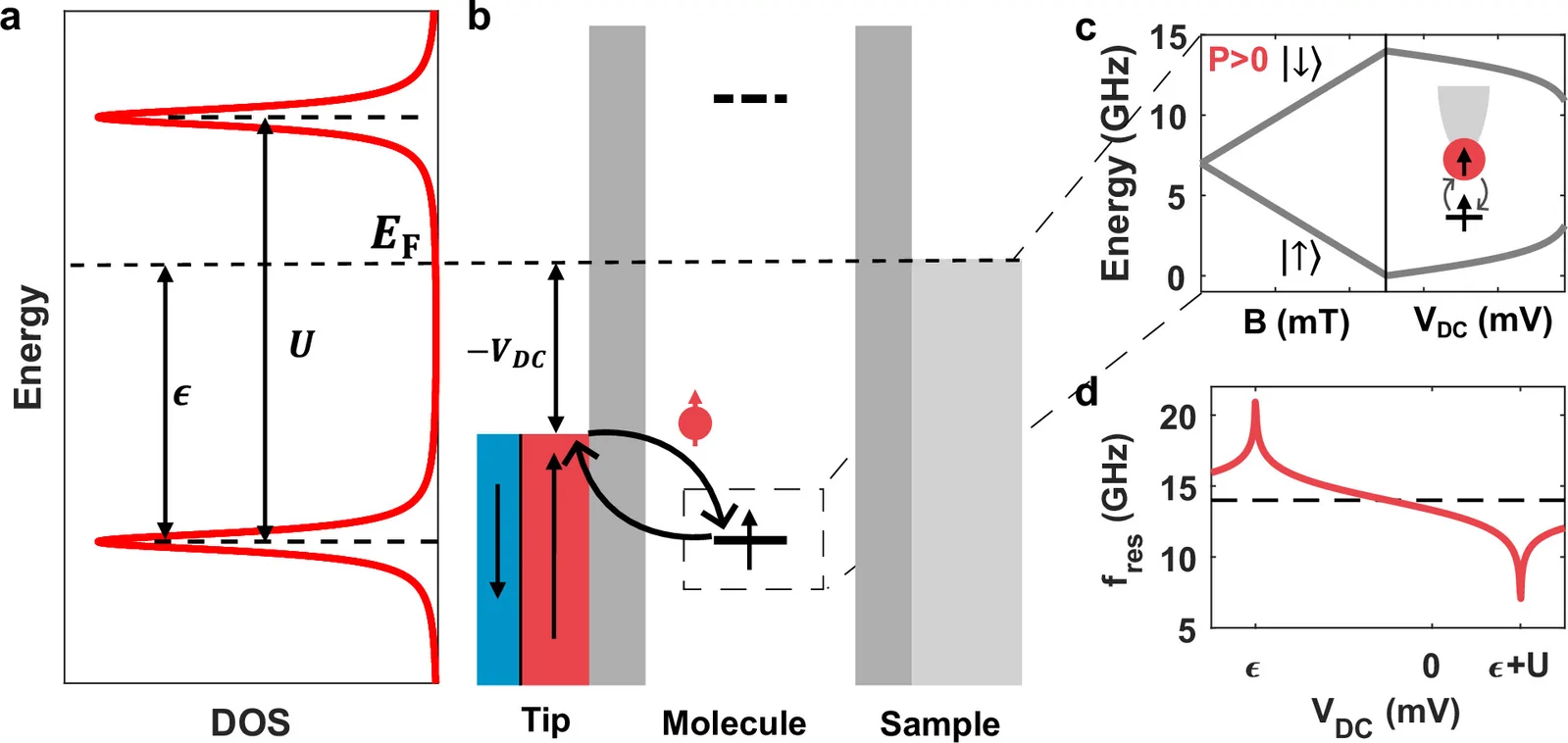
Single Impurity Anderson Model and Exchange Bias. **a**, Density of states (DOS) of the single impurity Anderson model (SIAM) with the ionization energy ϵ and the Coulomb repulsion energy U. **b**, Schematic drawing of the tunnel junction. The energy levels from the SIAM are located between the spin-polarized tip (left) and the non-magnetic sample electrode (right). The energy barriers stemming from the vacuum and MgO are illustrated by dark grey boxes. The applied bias voltage ${V}_{{\rm{DC}}}$ shifts the tip’s chemical potential relative to the molecule and sample. The virtual tunneling process which mediates the exchange coupling is illustrated by the black curved arrows. **c**, Energy level diagram of a spin under the influence of an external magnetic field B as well as ${V}_{{\rm{DC}}}$ for a positively spin-polarized tip (P>0). The Zeeman splitting caused by ${B}$ is followed by an energy correction due to the transport mediated exchange coupling as a function of ${V}_{{\rm{DC}}}$. **d**, Resonance frequency ${f}_{{\rm{res}}}$ calculated for a spin S = 1/2 system within the SIAM as a function of ${V}_{{\rm{DC}}}$. As a reference, the dashed black line shows the resonance frequency in the absence of tip field. The exchange bias features a logarithmic divergence at ϵ and ϵ+U.

**Extended Data Fig. 5:**
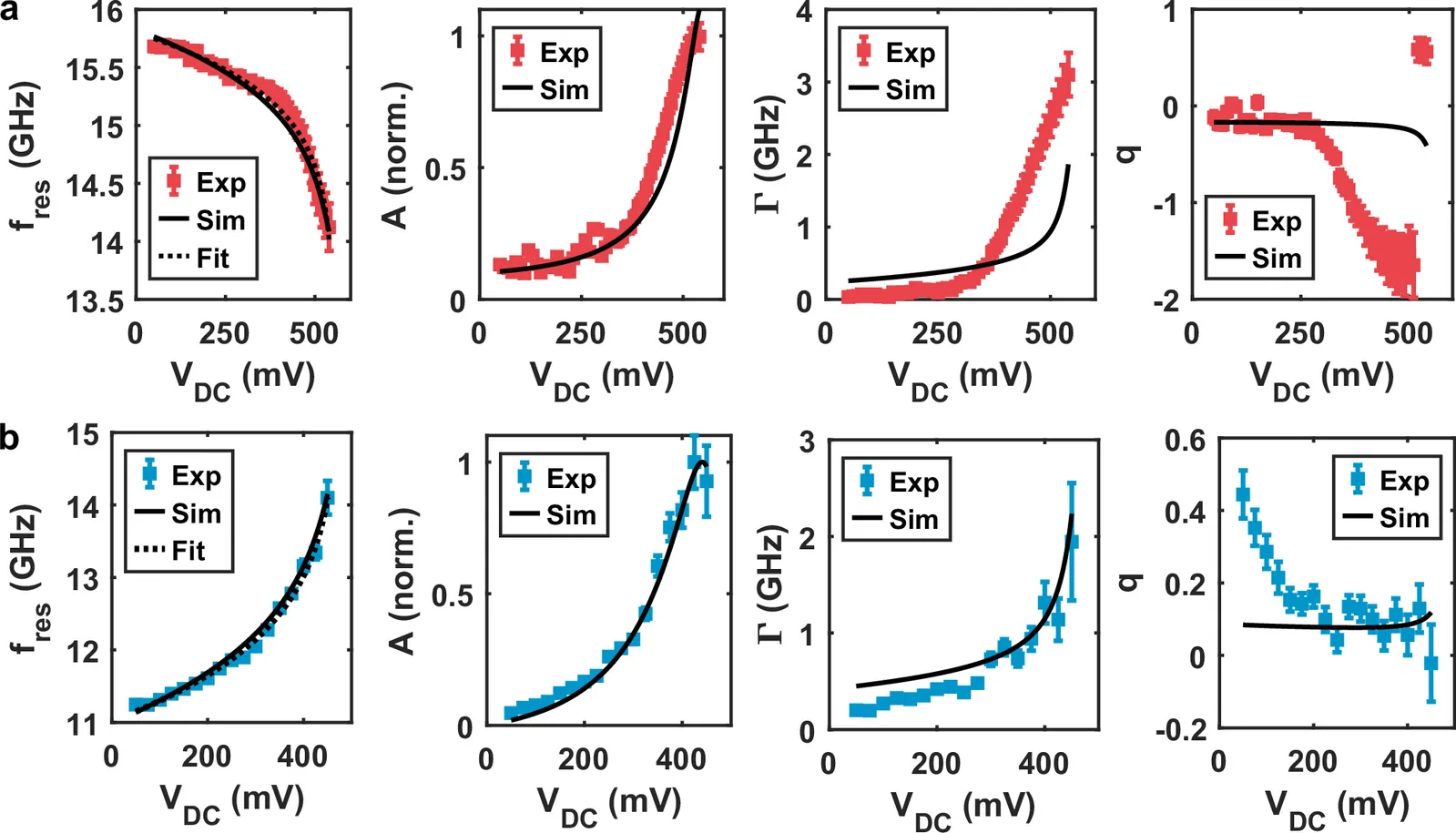
Comparison of the ESR peak parameters. **a**, Comparison between experiment and simulation for the data presented in Fig. 2a. The panels present the extracted parameters from Eq. (S1) ${(f}_{{\rm{res}}},{A},\,\Gamma ,{q}$) to the respective frequency sweeps as a function of ${V}_{{\rm{DC}}}$. The resonance frequencies obtained from the experiment are shown in red with error bars from the Fano fits. Simulation results are shown in black. The leftmost panel showing ${f}_{{\rm{res}}}$ additionally includes the fit to the analytical formula presented in Eq. (2). **b**, Same analysis as in **a**, applied to the data from Fig. 2b with the experiment in blue.

Here, the first term is the exchange field component along the quantization axis of the surface spin. The spin polarization $P=\frac{{n}_{\uparrow }-{n}_{\downarrow }}{{n}_{\uparrow }+{n}_{\downarrow }}$ quantifies the imbalance of the density of spin up (${n}_{\uparrow }$) and down electrons (${n}_{\downarrow }$) and sets the direction of the observed frequency shift ascribed to the tip. The coupling between the molecule and the tip ${\gamma }_{{\rm{T}}}$ can be controlled in the experiment via the conductance setpoint ${\gamma }_{{\rm{T}}}\propto G$, which alters the width of the vacuum barrier. e is the electron charge, and ${B}_{0}$ accounts for a residual tip field, stemming for instance from magnetic dipole contributions. The logarithmic relation between ${V}_{{\rm{DC}}}$ and the energy levels of the molecule,$\,\epsilon$ (ionization energy) and ϵ+U (U is the Coulomb repulsion energy) results in a nonlinear, diverging behaviour close to these energy levels (see Extended Data Fig. 4 and Supplementary Section 6.3 for details). Using the model, we can describe the experimental data in Fig. 2a (Fig. 2b) with a positive (negative) tip polarization: In Fig. 2c, we first use equations (1) and (2) to fit the nonlinear divergence of $\Delta f({V}_{{\rm{DC}}})$. Here, we use the FePc HOMO level, obtained in Fig. 1b, and fix *ε* = 2,000 meV. We subsequently find $\left(\epsilon +U\right)=553\pm 6\,{\rm{meV}}$ and $477\pm 10\,{\rm{meV}}$ for positive (P>0) and negative (P<0) tip polarizations, respectively. The observed deviation can be explained by differences in the orbital energies for the two different molecules, together with variations in the setpoint conductance. Overall, the obtained ϵ+U is in good agreement with the conductance peak associated with the LUMO from Fig. 1b. Here, we attribute the difference to the observed maximum in d*I*/d*V* (Fig. 1b, ${E}_{{\rm{L}}}\approx \mathrm{1,000}\,{\rm{meV}}$) to the onset of multiple orbital states inside the d*I*/d*V* peak (Extended Data Figs. 1 and 2 and Supplementary Sections 4 and 9). We note that the transition from molecular orbitals to the single-impurity Anderson model of the exchange bias model is not trivial. However, our DFT calculations (Extended Data Fig. 2) indicate that the spin-carrying orbital is a single orbital with strong *d*-character, which can be well approximated by a single-impurity Anderson model. We reproduce the data in Fig. 2a,b by performing full transport simulations, which aim to capture all features of the ESR–STM spectrum. The results are presented in Fig. 2a,b alongside the experimental data and show a close agreement in amplitude and resonance frequency of the peak (Extended Data Fig. 5). The convincing match between experiment and theory in Fig. 2a–c supports that the SEC is a result of the exchange bias mechanism outlined above.

### Coherent spin control of molecules

**Fig. 3:**
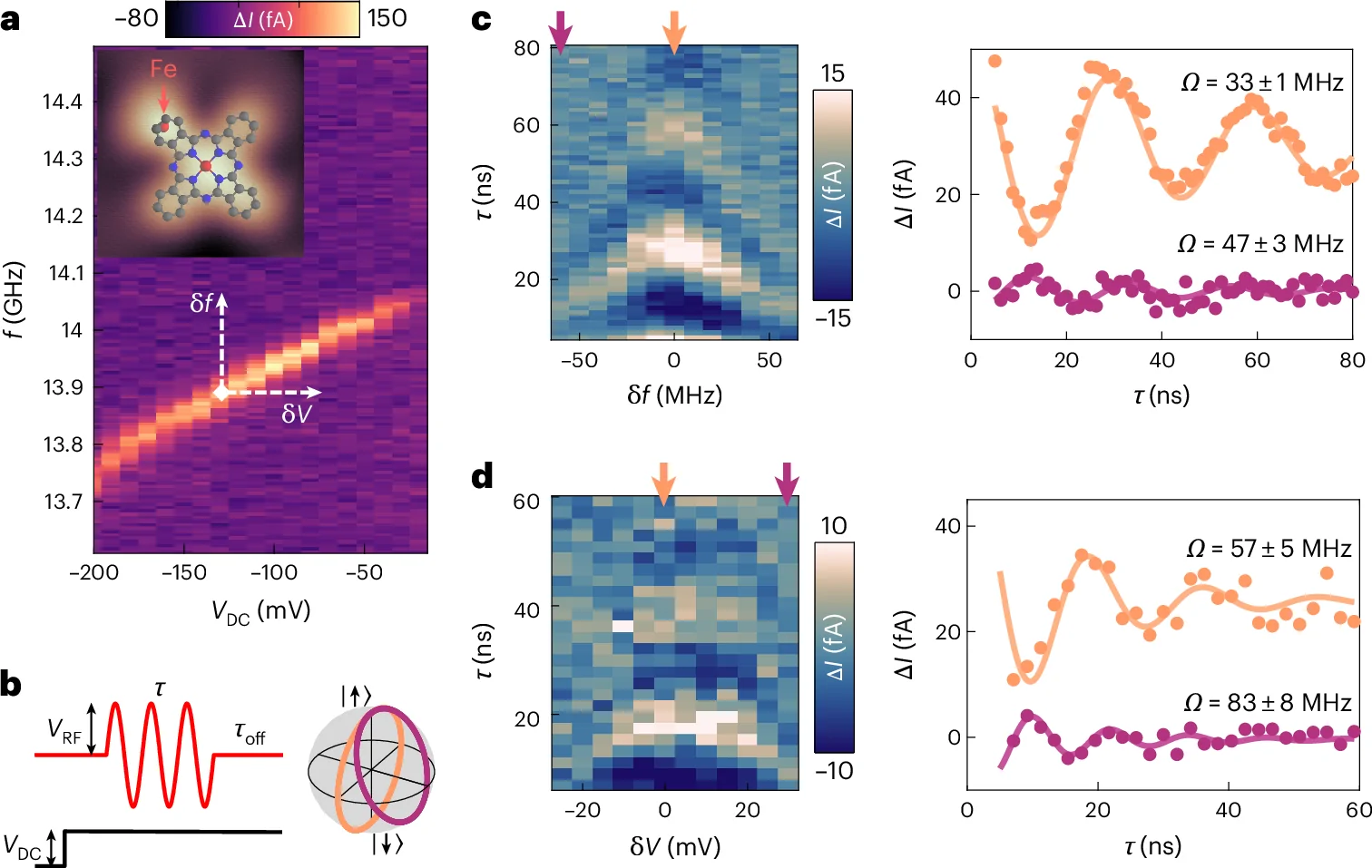
Spin–electric Rabi detuning on an Fe–FePc complex. **a**, ESR colour map $$\Delta I\left(f,\,{V}_{{\rm{DC}}}\right)$$ on the Fe site of an Fe–FePc complex (ESR conditions: $${I}_{\mathrm{set}}=6\,\mathrm{pA},\,{V}_{\mathrm{set}}=-60\,\mathrm{mV},B=469\,\mathrm{mT},\,{V}_{\mathrm{RF}}=10\,\mathrm{mV}$$). The chemical structure of the Fe–FePc is overlaid on the inset topography. The added Fe atom is highlighted by a red arrow, marking the site at which the measurements shown in **c** and **d** were performed. White arrows indicate the detuning in frequency $${\rm{\delta }}f=f-{f}_{\mathrm{res}}$$ and voltage $${\rm{\delta }}V={V}_{\mathrm{DC}}-{V}_{\mathrm{set}}$$ from the resonance. **b**, Left: schematic drawing of the Rabi pulse scheme. The RF signal consists of an RF pulse with duration τ and amplitude $${V}_{{\rm{RF}}}$$ followed by an off-time $${\tau }_{{\rm{off}}}$$. The total cycle time $${\tau }_{{\rm{cycle}}}=\tau +{\tau }_{{\rm{off}}}$$ is kept constant, and a d.c. voltage $${V}_{{\rm{DC}}}$$ is applied continuously for readout. Right: Bloch sphere representation of the spin evolution on resonance (orange) and off resonance (purple). **c**, Rabi oscillations for different frequency detuning $${\rm{\delta }}f$$ (Rabi conditions: $$\,{I}_{\mathrm{set}}=4\,\mathrm{pA},\,{V}_{\mathrm{set}}=-60\,\mathrm{mV},\,$$$$B=473\,\mathrm{mT},\,{V}_{\mathrm{RF}}=60\,\mathrm{mV},\,$$$$f=14.04\,\mathrm{GHz},\,{\tau }_{\mathrm{cycle}}=\,250\,\mathrm{ns}$$). Left: colour map of ΔI as a function of $${\rm{\delta }}f$$ and τ. The arrows refer to the traces shown on the right. Right: single traces on (orange) and off (purple) resonance, plotting ΔI as a function of τ. Solid lines are fits based on equation (3) (see Extended Data Table 1 for the parameters). Traces are vertically shifted for clarity. **d**, Rabi oscillations for detuning the voltage $${\rm{\delta }}V$$ instead of $${\rm{\delta }}f$$ (Rabi conditions: $${I}_{\mathrm{set}}=5\,\mathrm{pA},\,{V}_{\mathrm{set}}=-60\,\mathrm{mV},\,$$$$B=450\,\mathrm{mT},\,{V}_{\mathrm{RF}}=20\,\mathrm{mV},\,$$$$f=14.25\,\mathrm{GHz},\,{\tau }_{\mathrm{cycle}}=\,400\,\mathrm{ns}$$). Left: colour map of $$\Delta I({\rm{\delta }}V,\tau )$$. Right: single traces analogous to **c**.

**Extended Data Fig. 6:**
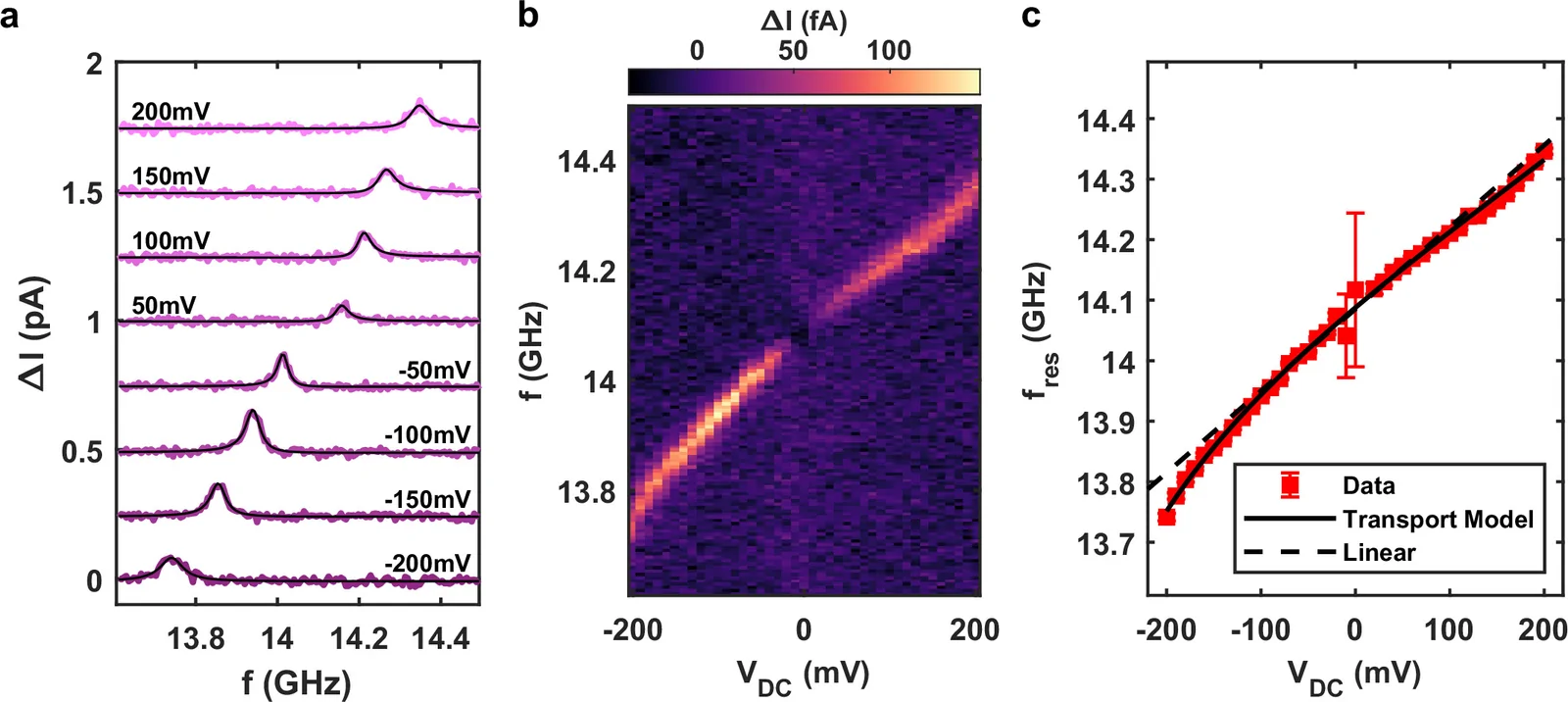
Spin-electric coupling measurement on a Fe-FePc complex. **a**, Waterfall plot of frequency sweeps showing ΔI as a function of f for several $${V}_{{\rm{DC}}}$$ measured on the Fe-site of a Fe-FePc complex (ESR conditions: $${I}_{{\rm{set}}}=6{\rm{pA}},\,{V}_{{\rm{set}}}=-60\,{\rm{mV}},\,{V}_{{\rm{RF}}}=10\,{\rm{mV}},{B}=469\,{\rm{mT}}$$). The solid black lines represent Fano fits to the experimental data (purple). With changing $${V}_{{\rm{DC}}}$$ we see a clear shift of the resonance position. **b**, Corresponding colormap $$\Delta I(f,{V}_{{\rm{DC}}})$$ of the data set, revealing a higher amplitude for negative voltages. **c**, Extracted resonance frequency $${f}_{0}$$ over $${V}_{{\rm{DC}}}$$ shown in red. Values and error bars are obtained from Fano fits to the frequency sweeps displayed in **b**. A linear fit (dashed line) ranging from $$-100\,{\rm{mV}}$$ to $$100\,{\rm{mV}}$$ deviates at higher voltages from the experimental data. A fit using the exchange bias model (solid line) described by Eq. (2) in the main text captures the non-linear behavior at higher voltage magnitudes. The fitted parameters are: $$\epsilon =\,-344\pm 47\,\mathrm{meV},$$$$U=998\pm 269\,\mathrm{meV},\,$$$${B}_{0}=41\pm 4\,\mathrm{mT},\,$$$${\gamma }_{{\rm{T}}}=66.5\pm$$$$14.8\,\mathrm{mT},P=-1$$. We find ϵ and U to be consistent with the values obtained from d*I*/d*V* measurements (see Fig. S4a).

To further demonstrate that a strong SEC enables all-electrical spin control, we utilize the SEC in coherent control schemes, for which we employ Fe–FePc complexes (Fig. 1a and Fig. 3a, inset). The ESR colour map in Fig. 3a shows the shift of ${f}_{{\rm{res}}}$ as a function of ${V}_{{\rm{DC}}}$. For the complex we also find an onset of nonlinear behaviour (Extended Data Fig. 6), while performing ESR at $\left|{V}_{{\rm{DC}}}\right| > 300\,{\rm{mV}}$ remains challenging. Nevertheless, the spin complex is generally easier to use in coherent control experiments than pristine FePc (see ref. ^33^ and Supplementary Section 5). The pulse scheme used for Rabi oscillation measurements is depicted in Fig. 3b (refs. ^21,22^). The resulting coherent oscillation of the spin state leads to a change in tunnel current ΔI as a function of the radio-frequency (RF) pulse duration τ (ref. ^21^):

3 $$\Delta I=A \sin \left(\varOmega \tau +\phi \right) {{\rm{e}}}^{-\tau /{T}_{2}}.$$

With the amplitude A, the Rabi rate Ω, the Rabi phase ϕ and phase coherence time ${T}_{2}$. Moreover, detuning from resonance ${\rm{\delta }}f=f-{f}_{\mathrm{res}}$ leads to a change in both amplitude A and Rabi rate Ω of the observed oscillation:

4 $$\begin{array}{cc}\varOmega =\sqrt{{\varOmega }_{0}^{2}+{{\rm{\delta }}f}^{2}}, & A={A}_{0}\frac{{\varOmega }_{0}^{2}}{{\varOmega }^{2}}\end{array}.$$

Here ${A}_{0}$ and ${\varOmega }_{0}$ are the parameters at ${f}_{{\rm{res}}}$. Consequently, the Rabi oscillations $\Delta I(\tau )$ can be tuned by ${\rm{\delta }}f$, resulting in the typical chevron pattern (Fig. 3c). Utilizing the SEC, we now realize an all-electrical detuning via a change in voltage ${\rm{\delta }}V={V}_{\mathrm{DC}}-{V}_{\mathrm{set}}$ (Fig. 3d) while keeping ${\rm{\delta }}f=0$. We obtain a chevron pattern as well for $\Delta I(\tau ,{\rm{\delta }}V)$, in which the amplitude A (Rabi rate Ω) decreases (increases) for increasing |${\rm{\delta }}{V|}$. Compared with the frequency tuning, the pattern is slightly distorted. We attribute this to a linear contribution to ${\varOmega }_{0}\propto {V}_{\mathrm{DC}}$ predicted for spin resonance in the exchange bias model^29,30^. In addition, we expect a dependence of the amplitude with tunnelling current $A\propto I\propto$ ${V}_{{\rm{DC}}}$ (see Supplementary Section 10 for details).

### Coherent control of a molecule dimer

**Fig. 4:**
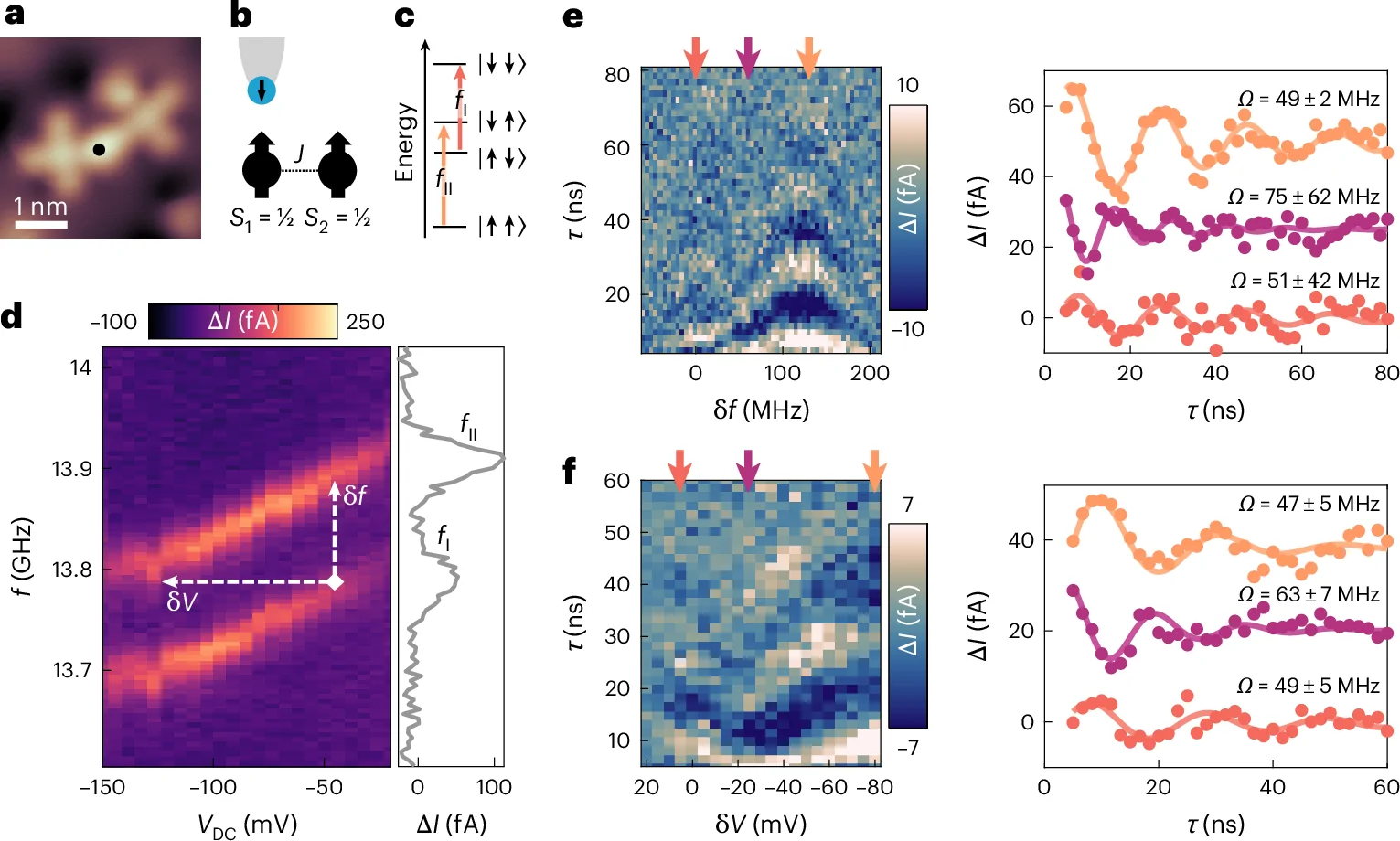
Spin–electric Rabi detuning in a coupled spin system. **a**, Topography of two coupled Fe–FePc complexes (image conditions: $I=10\,{\rm{pA}},\,{V}_{{\rm{DC}}}=-100\,{\rm{mV}}$). The black dot marks the tip position of the subsequent measurements. **b**, Schematic drawing of the two spin ½ with their exchange coupling J and the tip above the first spin ${S}_{1}$. **c**, Schematic energy level diagram of the combined spin states with the two ESR transitions ${f}_{{\rm{I}}}$ and ${f}_{{\rm{II}}}$. **d**, ESR colour map $\Delta I(f,\,{V}_{{\rm{DC}}})$ measured on the coupled spin system (ESR conditions: ${I}_{{\rm{set}}}=8\,{\rm{pA}},\,{V}_{{\rm{set}}}=-40\,{\rm{mV}},{B}=462\,{\rm{mT}},\,{V}_{{\rm{RF}}}=10\,{\rm{mV}}$). A single frequency sweep (right) at $-50\,{\rm{mV}}$ reveals two distinct peaks corresponding to ${f}_{{\rm{I}}}$ and ${f}_{{\rm{II}}}$, that is, transitions corresponding to different spin states of the remote spin ${S}_{2}$. White arrows indicate the detuning in frequency ${\rm{\delta }}f$ and voltage ${\rm{\delta }}V$. **e**, Frequency detuning of Rabi oscillations (Rabi conditions: ${I}_{{\rm{set}}}=8\,{\rm{pA}},\,{V}_{{\rm{set}}}=-40\,{\rm{mV}},{B}=458\,{\rm{mT}},\,{V}_{{\rm{RF}}}=80\,{\rm{mV}},{f}=13.97\,{\rm{GHz}}$). Left: colour map of ΔI as a function of ${\rm{\delta }}f$ and τ. The pattern shows two chevrons corresponding to the two ESR transitions. The arrows at the top refer to the single traces to the right. Right: single traces of $\Delta {I}$ versus τ for three ${\rm{\delta }}f$. Solid lines show fits to the circular datapoints based on equation (3) (see Extended Data Table 1 for the parameters). The traces were shifted vertically for clarity. **f**, Electric detuning of Rabi oscillations, analogous to **e** (Rabi conditions: ${I}_{{\rm{set}}}=8\,{\rm{pA}},\,{V}_{{\rm{set}}}=-40\,{\rm{mV}},{B}=462\,{\rm{mT}},\,{V}_{{\rm{RF}}}=70\,{\rm{mV}},{f}=14.04\,{\rm{GHz}}$). Left: colour map of $\Delta I({\rm{\delta }}V,\tau )$ showing the continuous electrical tuning from one ESR transition to the other. Right: ΔI as a function of τ for three different ${\rm{\delta }}V$ (also see Supplementary Section 12).

**Extended Data Fig. 7:**
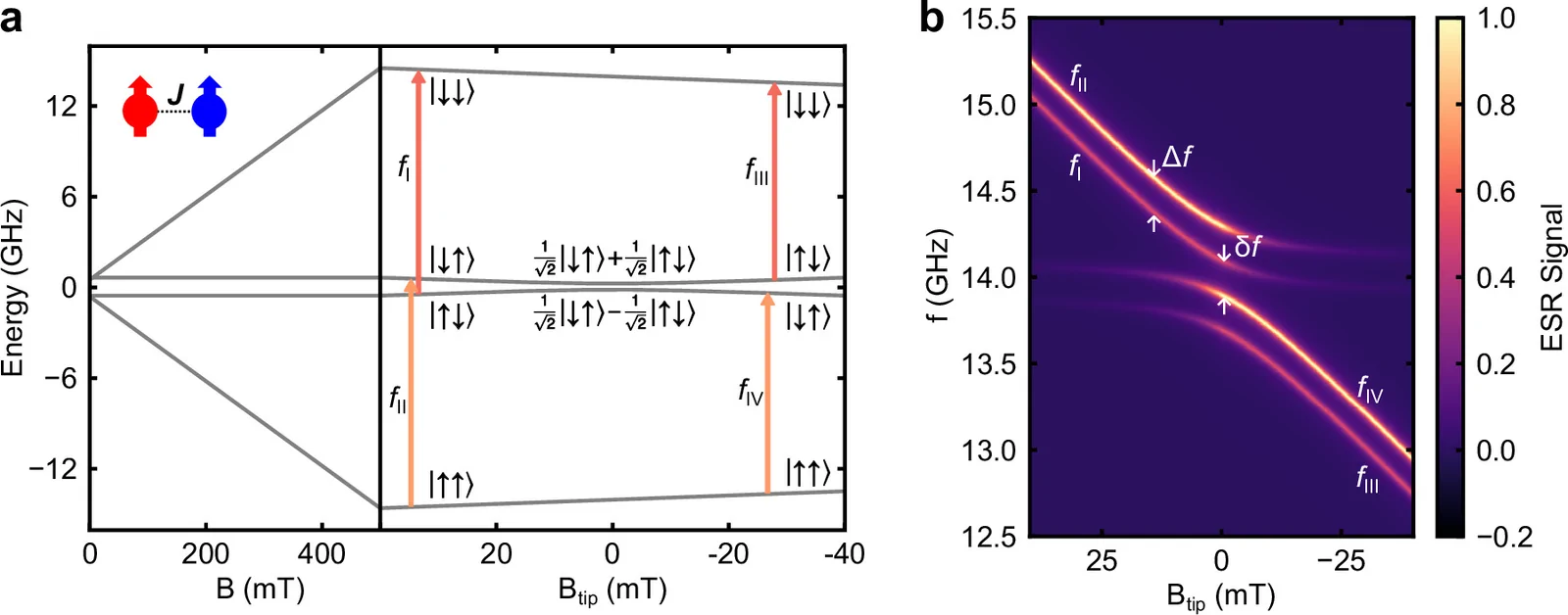
Coupled spin model and simulation. **a**, Zeeman diagram of two coupled S = 1/2 plotting the energies of the four eigenstates corresponding to Eq. (S12) over the external magnetic field B and the tip field ${B}_{{\rm{tip}}}$. The tip field, causing a mismatch of the two Zeeman energies of the spins, tunes the eigenstates from mixed states to Zeeman product states. The colored arrows show the possible transitions when performing ESR on the first spin. **b**, Simulation of the corresponding ESR signal as a function of frequency and tip field. Besides shifting the resonances to lower values, the tuning with ${B}_{{\rm{tip}}}$ causes a continuous evolution between the observable transitions with all four accessible at *B*_tip_ = 0.

Finally, we realize the SEC detuning in a two-spin system. In Fig. 4a, two complexes are brought into proximity using tip-assisted manipulation to establish a coupled spin system. The resulting configuration (Fig. 4b) consists of a readout spin ${S}_{1}$ that is ferromagnetically coupled to the second spin ${S}_{2}$ mainly through Heisenberg exchange interaction J (Extended Data Fig. 7 and Supplementary Section 11). Because the coupling is substantially smaller than the Zeeman energy, the system exhibits four distinct energy levels (Fig. 4c). The two resulting ESR transitions ${f}_{{\rm{I}}}$ and ${f}_{{\rm{II}}}$ (Fig. 4c,d) primarily reflect the alignments of ${S}_{2}$ in either *|*↑〉 and *|*↓〉 state^43,44^. ${f}_{{\rm{I}}}$ and ${f}_{{\rm{II}}}$ shift again as a function of ${V}_{{\rm{DC}}}$, with the exchange bias from the tip acting on ${S}_{1}$. The energy splitting ${{f}_{\mathrm{II}}-f}_{{\rm{I}}}\,\approx \,130\,\mathrm{MHz}$ remains constant across the whole voltage range, indicating that the spin–spin coupling between ${S}_{1}$ and ${S}_{2}$ is unaffected by the SEC. In the corresponding Rabi oscillation measurements (Fig. 4e), we now tune from ${f}_{{\rm{I}}}$ to ${f}_{{\rm{II}}}$ by changing ${\rm{\delta }}f$, which leads to two chevron patterns. The weaker intensity of the left chevron arises from the low thermal population of the excited state $|\downarrow {{\rangle }}$ of ${S}_{2}$. Again, the SEC enables all-electrical detuning via a change in voltage ${\rm{\delta }}V$ (Fig. 4f). The main limitation in this approach is the increased tunnelling current at higher voltages, which induces spin relaxation and decoherence^21,22^. However, the bias-controlled exchange field still permits to tune from the first transition (${\rm{\delta }}V\approx 5\,\mathrm{mV}$) to the second (${\rm{\delta }}V\approx -80\,\mathrm{mV}$).

## Conclusion

**Extended Data Fig. 8:**
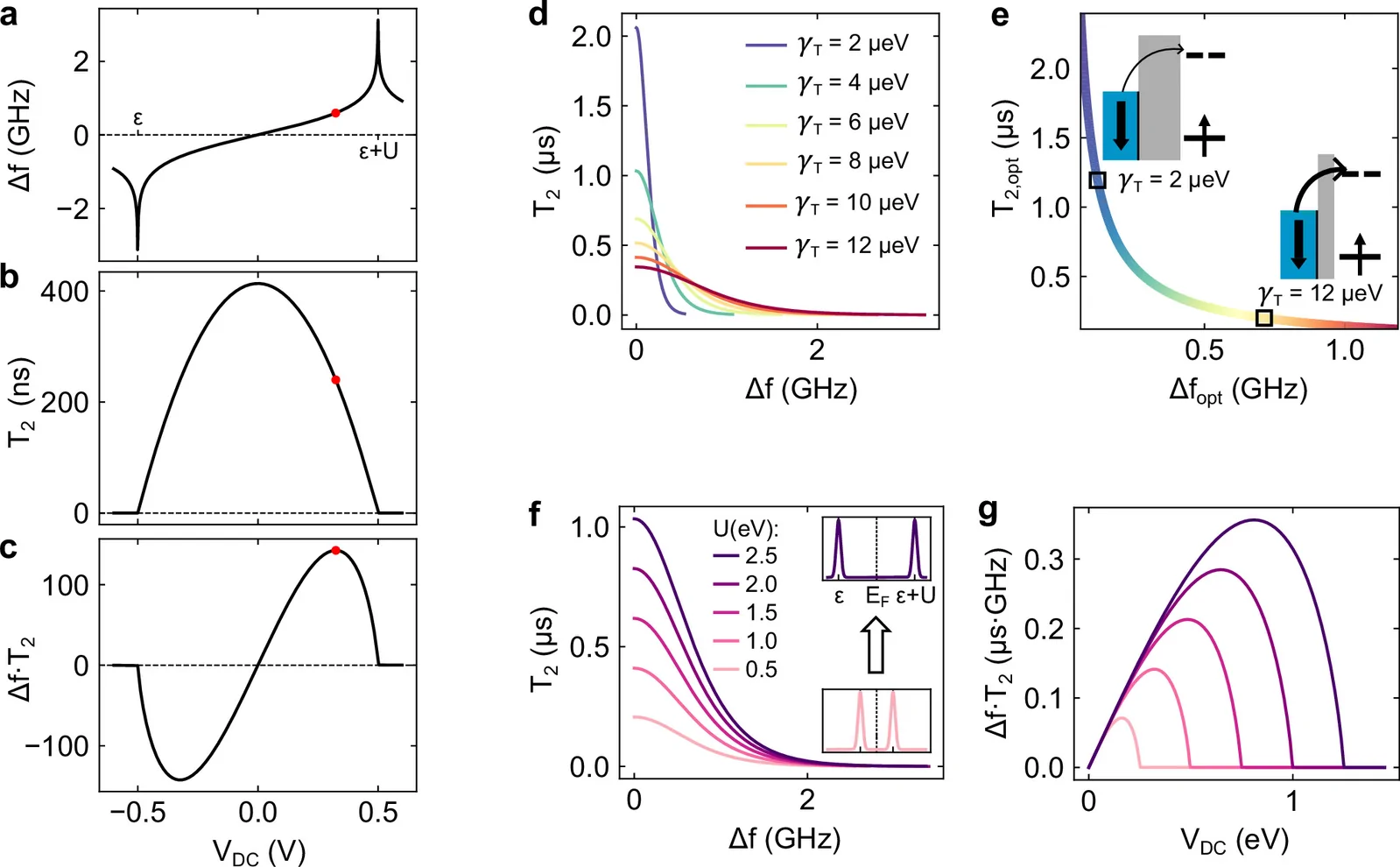
Operation strategy of the exchange bias. **a**, Δf from the exchange bias as a function of ${V}_{{\rm{DC}}}$ according to Eq. (2). **b**, ${T}_{2}$ as a function of ${V}_{{\rm{DC}}}$ according to Eq. (S17). **c**, $\Delta f\times {T}_{2}({V}_{{\rm{DC}}})$ with the maximum at ${V}_{{\rm{opt}}}$ marked with a red dot. (a-c) use: $U=1\,{\rm{eV}},\,\epsilon =-0.5\,{\rm{eV}},\,\Delta =0.25\,{\rm{meV}},\,{\gamma }_{{\rm{T}}}=10\,{\rm{\mu }}{\rm{eV}},{P}=-1$. **d**, ${T}_{2}$ as a function of Δf for several coupling strengths ${\gamma }_{{\rm{T}}}$. **e**, Plot of the decoherence time over the frequency shift at ${V}_{{\rm{opt}}}$ for different couplings ${\gamma }_{{\rm{T}}}=1\,{\rm{\mu }}{\rm{eV}}$ to $20\,{\rm{\mu }}{\rm{eV}}$. The insets show schematic drawings of the tunnel junction illustrating a weak (left) and strong (right) coupling ${\gamma }_{{\rm{T}}}$. **f**, ${T}_{2}$ as a function of Δf for several U. The DOS in the inset illustrates how larger U shifts the energy states. **g**, $\Delta f\times {T}_{2}({V}_{{\rm{DC}}})$ for the U values used in **f**.

Our measurements highlight that molecular spin systems can be tuned electrically via the bias voltage ${V}_{{\rm{DC}}}$. In particular, the ESR measurements near the LUMO of FePc suggest that the strong nonlinear SEC arises from the exchange bias due to enhanced virtual tunnelling. While this does not exclude the existence of other contributions, our results indicate that, in the present molecular systems, exchange bias is the dominant effect. Notably, the exchange bias mechanism has several important implications. First, unlike piezoelectric models, it does not require displacement of the molecule or any of its components, which extends applicability to a broader class of molecular systems, including rigid solid-state defects. Second, it permits the integration of molecular spins into devices, where they can be readily tuned via the polarization of nearby ferromagnetic electrodes. Third, the SEC strength observed here reaches close to 30% and is substantially larger than most reported electric tuning effects for molecular spins. In this regime, the resonance peak broadens due to increased decoherence from tunnelling electrons and enhanced electrode coupling. Nevertheless, we believe that, by carefully optimizing both the junction properties and the molecular orbital structure, a good compromise between SEC and preserving spin coherence can be achieved (Extended Data Fig. 8 and Supplementary Section 13).

**Extended Data Fig. 9:**
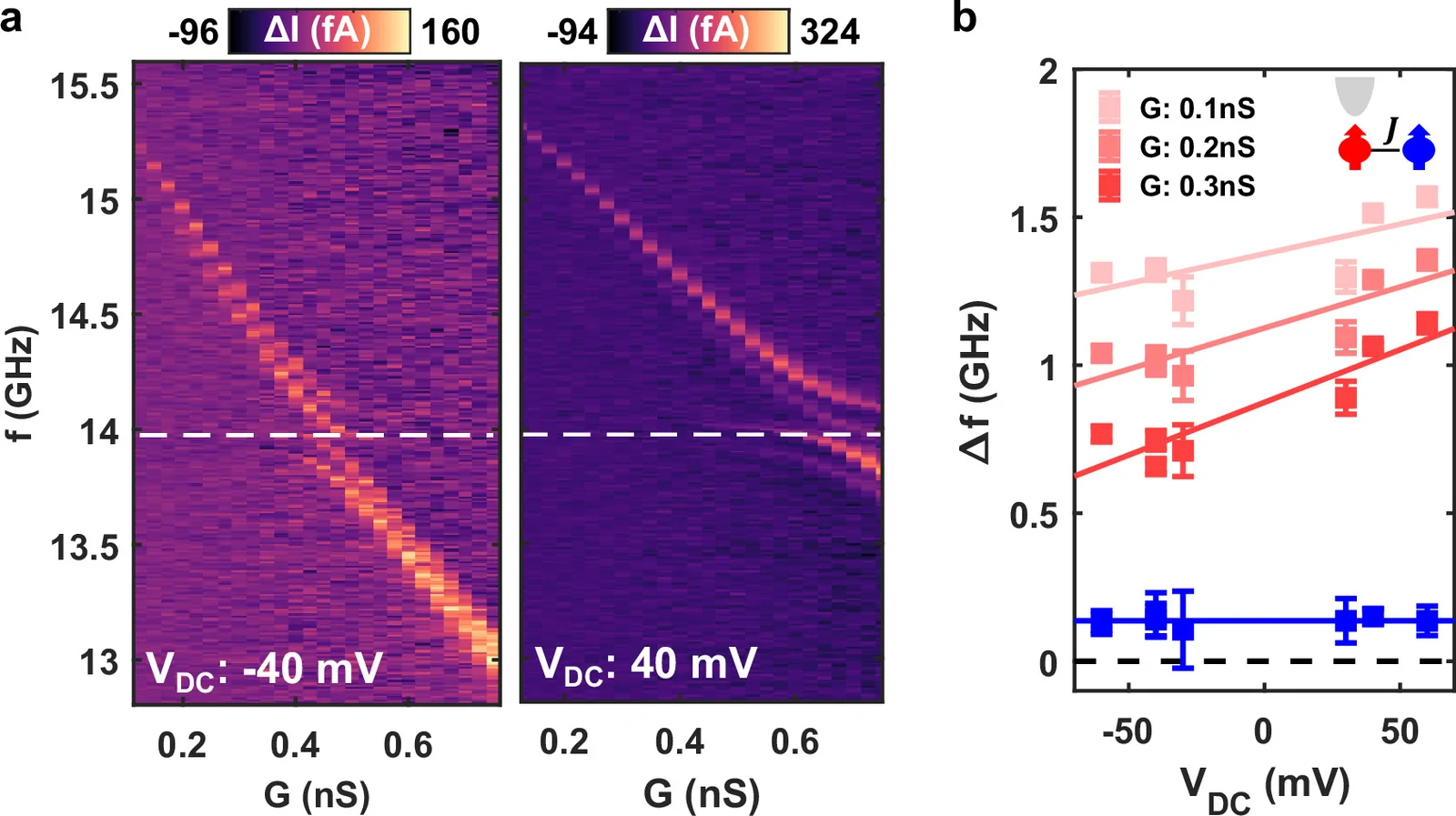
Spatial dependence of the exchange bias. **a**, Colormaps of $\Delta {I}$ as a function of f and G showing the avoided level crossing of the coupled spin system at ${V}_{{\rm{DC}}}=-40\,{\rm{mV}}$ and ${V}_{{\rm{DC}}}=40\,{\rm{mV}}$ (ESR conditions: ${V}_{{\rm{RF}}}=10\,{\rm{mV}},{B}=494\,{\rm{mT}}$). The white dashed lines show the frequency position of the avoided level crossing. **b**, Extracted frequency shift $\Delta f={f}_{{\rm{res}}}-2{\mu }_{{\rm{B}}}B/h$ as a function of ${V}_{{\rm{DC}}}$. Values and error bars are obtained from Fano fits to the corresponding frequency sweeps. Blue: Second spin with the mean value illustrated by the straight line. Red: Shift of the first spin for different G with linear fits shown as solid lines for visual aid. The inserted sketch at the top right corner reflects the measurement setup. The data shows that while the resonance frequency of the probed spin shifts with G and ${V}_{{\rm{DC}}}$, the resonance frequency of the coupled spin stays completely unaffected, demonstrating the strong localization of the exchange bias.

Finally, the results in Figs. 3 and 4 demonstrate not only the feasibility of combining coherent spin control with SEC, but also the ability to electrically tune one spin relative to another with nanometre precision. This precision arises from the particularities of the exchange bias: while a pure electric field from the tip apex would still act over distances exceeding tens of nanometres, the interplay between exchange interaction and the bias voltage—mediated by the magnetic tip electrode—localizes the SEC to the subnanometre scale. Further analysis of the data in Fig. 4 shows that only the molecular spin under the tip is tuned, while the other one stays completely unaffected (Extended Data Fig. 9). Crucially, the ability to tune between two distinct spin resonances represents a key step towards conditional spin control in coupled spin systems. Consequently, the potential for tuning spin dynamics via the exchange bias paves the way for fast all-electrical gate operations in larger molecular quantum systems.

## Methods

### Sample preparation

Sample preparation and all experiments were carried out in a Unisoku USM1600 system with a home-built dilution refrigerator. The data shown in Figs. 1 and 2 were measured at a base temperature of $1\,{\rm{K}}$, while the data in Figs. 3 and 4 were measured at 50 mK. The in situ sample preparation was performed under ultrahigh vacuum conditions with a base pressure of <5 × 10^−10^ mbar. The Ag(001) single crystal was first cleaned through multiple cycles of argon ion sputtering and subsequent annealing using an electron beam. MgO was grown by evaporating Mg in an oxygen-rich atmosphere (≈1 × 10^−6^ mbar) while maintaining the substrate at 510 °C. A deposition time of 10 min resulted in partial coverage (≈50%), with MgO islands ranging from two to five monolayers in thickness. FePc molecules were deposited onto the surface using a home-built Knudsen cell (deposition time 90 s, pressure 9 × 10^−10^ mbar). Afterwards, Fe atoms were deposited onto the cooled sample by electron-beam evaporation for $21\,{\rm{s}}$.

### Experimental set-up

For the ESR and Rabi measurements, we prepared spin-polarized tips by picking up 1–20 Fe atoms from the MgO surface. In most cases, the ESR active tips also showed strong asymmetries in d*I*/d*V* spectra around 0 V on FePc molecules due to inelastic electron tunnelling scattering. For the experiments shown, we applied the d.c. and RF voltage to the tip and corrected this to the convention that the voltage is applied to the sample. The RF signal was generated by a Rohde & Schwarz SMB100B generator and mixed with the d.c. bias voltage ${V}_{{\rm{DC}}}$ via a Marki Microwave MDPX-0305 Diplexer. For ESR, a magnetic field B is applied perpendicular to the sample surface. The presented ESR frequency sweeps were measured with an on/off modulation at 323 Hz, where the RF voltage ${V}_{{\rm{RF}}}$ in continuous-wave mode was present only in the A cycle. The applied ${V}_{{\rm{DC}}}$ was present in both the A and B cycles and therefore applied throughout the entire measurement. During the voltage-dependent frequency sweeps, the feedback of the STM controller was turned off to keep the position of the tip the same when altering ${V}_{{\rm{DC}}}$. The signal was read out via a Stanford Research Systems SR860 digital lock-in amplifier.

### Data evaluation

To analyse the frequency sweeps of our experiments, we fitted the following Fano function to our resonance peaks:

S1 $$\begin{array}{rcl}\Delta I=\frac{A}{{q}^{2}+1}\frac{{\left(q \epsilon +1\right)}^{2}}{1+{\epsilon }^{2}}+c & \mathrm{with} & \epsilon =\frac{f-{f}_{\mathrm{res}}}{0.5 \varGamma }\end{array},$$

with the amplitude A, the resonance frequency ${f}_{{\rm{res}}}$, the linewidth (full width at half maximum) Γ and the q-factor, which captures the asymmetry of the resonance peak.

For the Rabi measurements presented in Figs. 3 and 4, we followed the detection scheme introduced by ref. ^21^: instead of a continuous-wave RF signal, we applied pulse trains (Fig. 3b) with on-time τ and off-time ${\tau }_{{\rm{off}}}$, keeping the sum (${\tau }_{\mathrm{cycle}}$) fixed. The RF pulses were only present in the A cycle and triggered with a Zurich Instruments HDAWG. Therefore, the measured signal presents an average of the spin state-related tunnel current. For each data point τ, the signal was averaged for 3 s. After the data acquisition, a linear background^21^ caused by current rectification with increasing pulse duration was subtracted.

## Online content

Any methods, additional references, Nature Portfolio reporting summaries, source data, extended data, supplementary information, acknowledgements, peer review information; details of author contributions and competing interests; and statements of data and code availability are available at https://doi.org/10.1038/s41567-026-03353-w.

## Supplementary information

Supplementary Information Supplementary Sections 1–13, including Supplementary Figs. 1–14 and Table 1.

Peer Review File

## Data Availability

All data supporting this work are available via figshare at https://doi.org/10.6084/m9.figshare.31554586 (ref. ^47^).

## Extended data

**Extended Data Table 1:** Fit parameters of the Rabi measurements

Fig.	*δf* | *δV*	*A* (fA)	Ω (MHz)	*T*_2_ (ns)	*ϕ* (°)
3c	0 MHz	24 ± 4	33 ± 1	54 ± 12	8.5 ± 6.6
	-60 MHz	4 ± 3	47 ± 3	42 ± 38	154 ± 31
3d	0 mV	27 ± 17	57 ± 5	16 ± 10	-29 ± 25
	30 mV	9 ± 9	83 ± 8	15 ± 13	56 ± 43
4e	0 MHz	8 ± 5	51 ± 4	38 ± 36	-147 ± 40
	60 MHz	17 ± 10	75 ± 6	16 ± 9	-90 ± 29
	130 MHz	20 ± 4	49 ± 2	36 ± 11	-114 ± 16
4f	5 mV	6 ± 4	49 ± 5	31 ± 30	36 ± 42
	-25 mV	14 ± 8	63 ± 7	14 ± 8	-93 ± 35
	-80 mV	20 ± 10	47 ± 5	15 ± 8	8 ± 22

The table presents the found quantities of the Rabi oscillations in Figs. 3 and 4 from fits of Eq. (3) to the experimental data.
