# Supplementary material for: Exchange-mediated spin–electric control of single molecules on surfaces
Source: Nat Phys. 2026 Jun 29;22(9):1394–400. doi: 10.1038/s41567-026-03353-w (PMC13558043; doi:10.1038/s41567-026-03353-w)
Supplement: Supplementary file 1 [file 41567_2026_3353_MOESM1_ESM.pdf]

nature physics

Supplementary information https://doi.org/10.1038/s41567-026-03353-w

Exchange-mediated spin–electric control of single molecules on surfaces

In the format provided by the authors and unedited

Table of Contents

1. Sample Preparation 2
2. Methods and Data Evaluation 2
3. Properties of Iron Phthalocyanine on MgO 3
4. Density Functional Theory (DFT) of FePc 5
5. Properties of the Fe-FePc Complex on MgO 6
6. Spin-Electric Coupling Models 8
   6.1 Piezo-Electric Displacement with g-factor Modulation 8
   6.2 Piezo-Electric Displacement with Tip Exchange Field 8
   6.3 The Exchange Bias Model 10
7. Linear Spin-Electric Coupling (SEC) of FePc and Fe-FePc 12
8. Polarity Preference of FePc and Fe-FePc 14
9. Non-linear Spin-Electric Coupling of FePc 16
10. Coherent Control Experiments 19
11. SEC in a Coupled Spin System 22
12. Rabi Measurements in a Coupled Spin System 25
13. Operation and Implementation Strategies of the Exchange Bias 26
   13.1 Chemical Design Principles for Molecules 26
   13.2 Decoherence 26

1. Sample Preparation

Sample preparation and all experiments were carried out in a Unisoku USM1600 system with a home-built dilution refrigerator. The data shown in Fig. 1 and 2 were measured at a base temperature of $1\ \mathrm{K}$, while the data of Fig. 3 and 4 were measured at $50\ \mathrm{mK}$. The in-situ sample preparation was performed under ultra-high vacuum conditions with a base pressure of $< 5 \times 10^{-10}\ \mathrm{mbar}$. The Ag(001) single crystal was first cleaned through multiple cycles of argon ion sputtering and subsequent annealing using an electron beam. MgO was grown by evaporating Mg in an oxygen-rich atmosphere ($\sim 1 \times 10^{-6}\ \mathrm{mbar}$) while maintaining the substrate at $510°\ \mathrm{C}$. A deposition time of $10\ \mathrm{min}$ resulted in partial coverage ($\sim 50\%$) with MgO islands ranging from 2 to 5 monolayers in thickness. FePc molecules were deposited onto the surface using a home-built Knudsen cell (deposition time: $90\ \mathrm{s}$, pressure: $9 \times 10^{-10}\ \mathrm{mbar}$). Afterwards, Fe atoms were deposited onto the cooled sample by electron-beam evaporation for $21\ \mathrm{s}$.

2. Methods and Data Evaluation

For the ESR and Rabi measurements we prepared spin-polarized tips by picking up $1 - 20$ Fe atoms from the MgO surface. In most cases the ESR active tips also showed strong asymmetries in d*I*/d*V* spectra around $0\ \mathrm{V}$ on FePc molecules due to inelastic electron tunneling scattering. For the experiments shown, we applied the DC and RF voltage to the tip and corrected this to the convention that the voltage is applied to the sample. The RF signal was generated by a Rohde & Schwarz SMB100B generator and mixed with the DC bias voltage V_{DC} via a Marki Microwave MDPX-0305 Diplexer. For ESR, a magnetic field B is applied perpendicular to the sample surface. The presented ESR frequency sweeps were measured with an on/off modulation at $323\ \mathrm{Hz}$ where the RF voltage V_{RF} in continuous-wave (cw) mode was only present in the A-cycle. The applied V_{DC} was present in A- and B-cycle and therefore applied during the whole measurement. During the voltage dependent frequency sweeps the feedback of the STM controller was turned off to keep the position of the tip the same when altering V_{DC}. The signal was read out via a Stanford Research Systems SR860 digital lock-in amplifier. To analyze the frequency sweeps of our experiments we fitted the following Fano-function to our resonance peaks:

$$\Delta I = \frac{A}{q^2+1}\frac{(q\cdot\epsilon+1)^2}{1+\epsilon^2} + c \qquad \text{with} \qquad \epsilon = \frac{f - f_{\mathrm{res}}}{0.5\cdot\Gamma} \tag{S1}$$

with the amplitude A, the resonance frequency f_{res}, the linewidth (FWHM) Γ and the q-factor which captures the asymmetry of the resonance peak.

For the Rabi measurements presented in Fig. 3 and 4 we followed the detection scheme introduced by Ref.[1]: Instead of a cw RF signal, we applied pulse trains (see Fig. 3b) with on-time τ and off-time τ_{off} keeping the sum (τ_{cylce}) fixed. The RF pulses were only present in the A-cycle and triggered with a Zurich Instruments HDAWG. Therefore, the measured signal presents an average of the spin state-related tunnel

current. For each data point τ, the signal was averaged for $3\,\mathrm{s}$. After the data acquisition, a linear background[1] caused by current rectification with increasing pulse duration was subtracted.

3. Properties of Iron Phthalocyanine on MgO

When adsorbed on 2 ML of MgO, the ground state of FePc has been found to be singly negatively charged, i.e. $[FePc]^{-1}$ compared to its electronic configuration in vacuum. Here, the center Fe is in a $3d^7$ electron configuration. This results in an unpaired electron in a dz^2-like orbital leading to a S=1/2 magnetic state (see Ref.[2]). Additional information on the density functional theory (DFT) calculations is provided in Extended Data Fig. 2 and Section S4. Here, we present further experimental characterization.

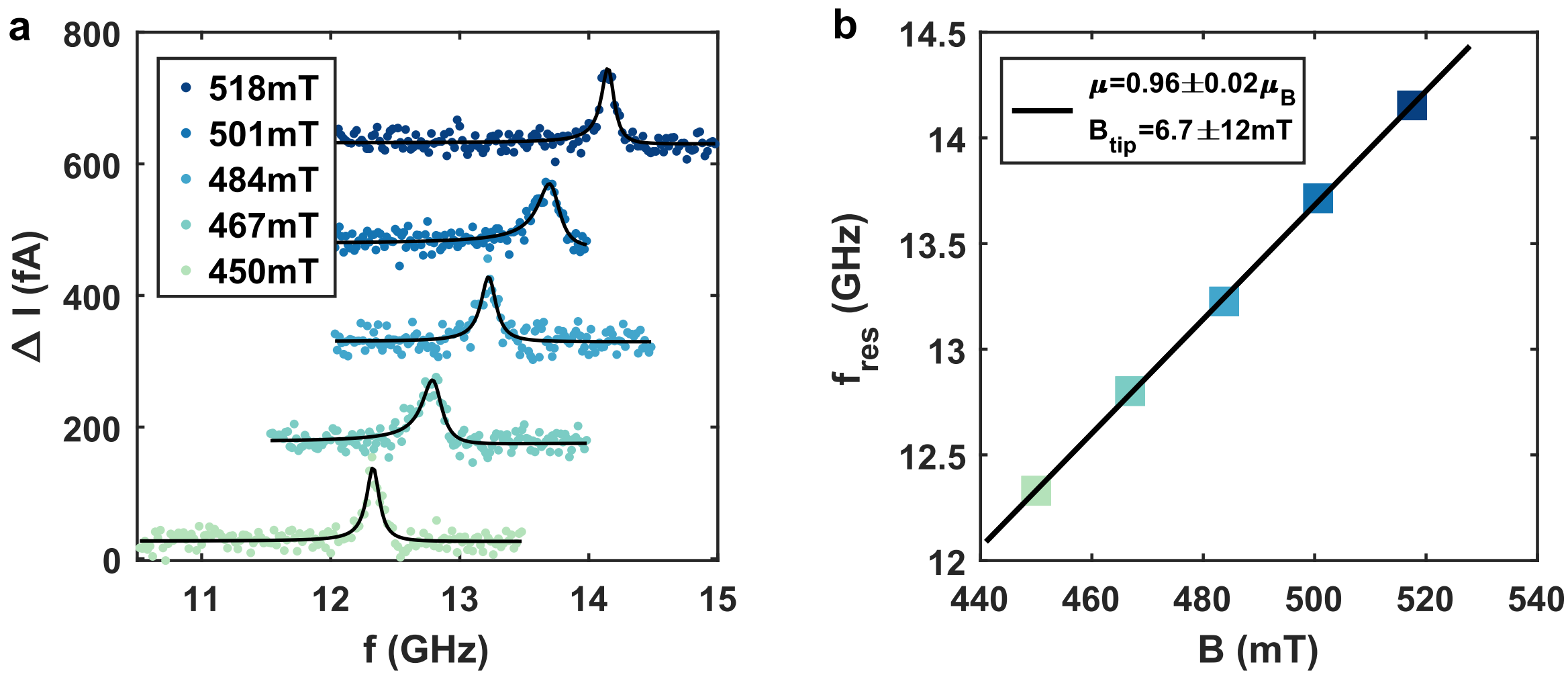

Supplementary Fig. S1. (a) ESR frequency sweeps measured at different magnetic fields B (ESR conditions: $I_{\mathrm{set}} = 8\,\mathrm{pA}, V_{\mathrm{set}} = 50\,\mathrm{mV}, V_{\mathrm{RF}} = 10\,\mathrm{mV}$). The traces were vertically shifted for clarity and the black solid lines are Fano fits to the data. **(b)** Determination of the magnetic moment μ. The extracted resonance frequencies from the fits in (a) are plotted as a function of B. The solid black line is a linear fit ($f_0 = \mu(B + B_{\mathrm{tip}})/h$) to the data, which yields $\mu = 0.96 \pm\ 0.02\,\mu_{\mathrm{B}}$ and $B_{\mathrm{tip}} = 6.7 \pm 12.0\,\mathrm{mT}$.

g-factor: The magnetic moment of the FePc molecule has been investigated in previous works[2,3]. While Zhang et al. find on average $\mu = 1.028 \pm\ 0.023\,\mu_{\mathrm{B}}$, Chen et al. find $\mu = 0.96 \pm\ 0.004\,\mu_{\mathrm{B}}$. In Fig. S1 we present our own measurement finding $\mu = 0.96 \pm\ 0.02\,\mu_{\mathrm{B}}$. Besides variations between molecules due to their surroundings, the tip magnetic field B_{tip} (consisting of dipole-dipole and exchange interaction) can affect the correct assignment of the g-factor[4]. Given these variations, we use $g = 2$ for the sake of simplicity in our analysis defining $f_0 = 2\mu_B B_{\mathrm{ext}}/h$.

HOMO/LUMO energies: In Fig. S2a we show d*I*/d*V* spectra measured on single FePc molecules. From the spectra we extract the energy position of the two peaks associated with resonant tunneling through the HOMO and LUMO. On average, we find $E_{\mathrm{H}} = -1948 \pm 127\,\mathrm{meV}$ (Fig. S2b) and $E_{\mathrm{L}} = 955 \pm 128\,\mathrm{meV}$ (Fig. S2c) in agreement with the values reported in Ref.[2]. We note here that the determination of the HOMO peak is rather difficult due to its low prominence. Variations for both HOMO and LUMO can be partially explained by different electrostatic environments of the measured molecule: Plotting $E_{\mathrm{L}} - E_{\mathrm{H}} = 2926 \pm 78\,\mathrm{meV}$ (Fig. S2d), we observe less

variations. A change in electrostatic potential can for instance be explained by trapped charges in / under the MgO substrate or proximity to edges, leading to local inhomogeneities. Moreover, a quantitative assignment of the peak positions in d*I*/d*V* to HOMO/LUMO is not so straightforward, which will be addressed in the next section via d*I*/d*V* maps.

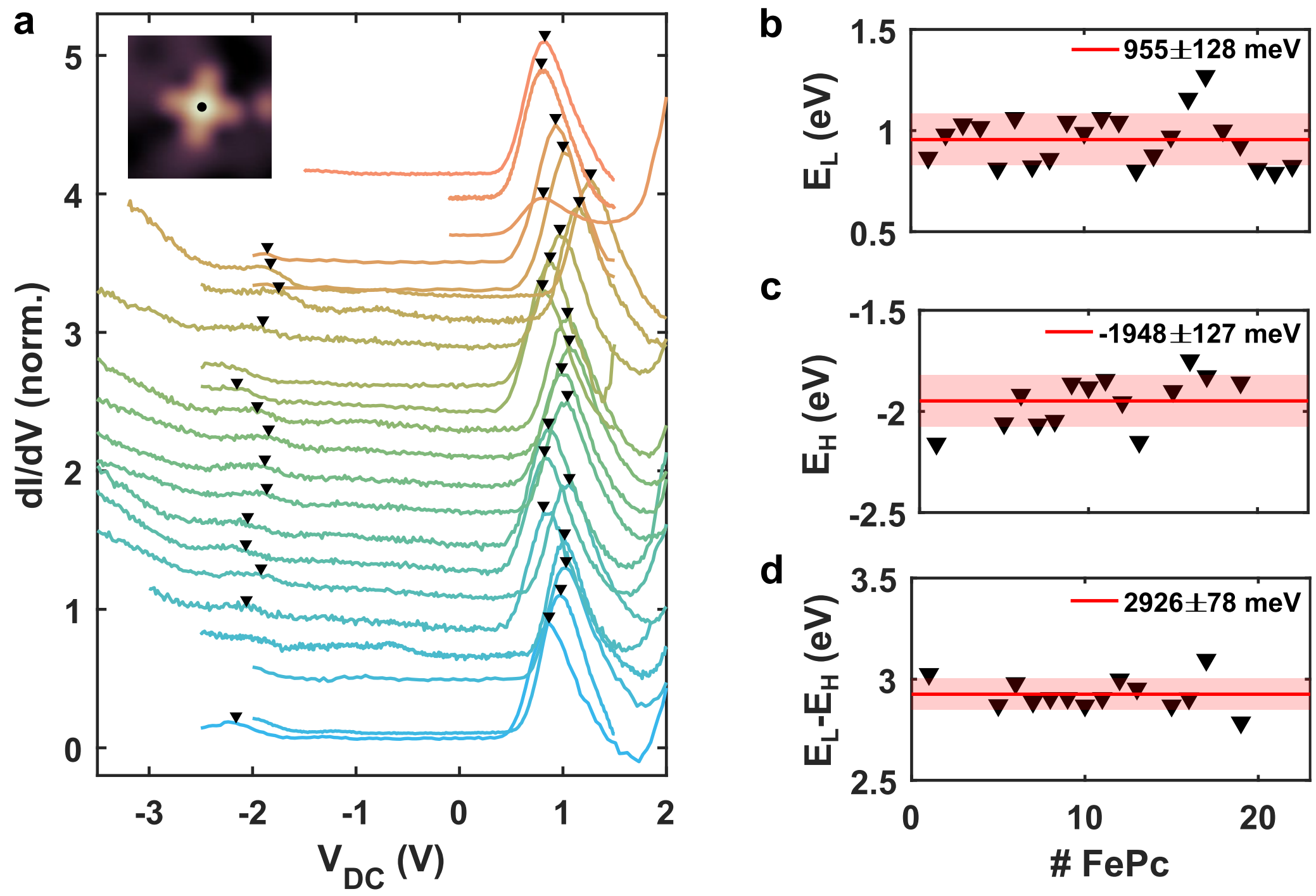

Supplementary Fig. S2. (a) d*I*/d*V* measurements on the FePc center obtained for different molecules and tips. The spectra were normalized by the value at the positive conductance peak and vertically shifted for clarity. The black rectangles mark the extracted positions of the HOMO (E_{H}) and LUMO (E_{L}). All measurements were performed at the center of the FePc as indicated by the black dot on the inserted topography. **(b)** Summary of the extracted E_{L} over the different molecules. The mean value and its standard deviation are plotted in red. **(c,d)** Analogous to b) for E_{H} and the difference between the two energies $E_{\mathrm{L}} - E_{\mathrm{H}}$.

d*I*/d*V* maps: In addition to Fig. 1b we provide more d*I*/d*V* maps on the FePc molecule in the Extended Data Fig. 1b. Extended Data Fig. 1a shows the d*I*/d*V* spectrum from Fig. 1b as a reference. We find the signal at $-2000\ \mathrm{mV}$ located at the center of the molecule, reflecting the HOMO. For positive voltages ($\approx 300 - 800\ \mathrm{mV}$), we see that the d*I*/d*V* signal spreads from the center towards the lobes, suggesting that tunneling into different orbitals contribute to the pronounced conductance peak[2]. We note that the association of the spatial shape of the d*I*/d*V* signal to molecular orbitals is not straightforward[5], since the pronounced signals observed in d*I*/d*V* measurements correspond to charge transitions which are best described via Dyson orbitals and may differ from the Kohn-Sham spin orbitals. Still, by comparison with DFT calculations of this system (next section) and the spatial information from d*I*/d*V* maps we conclude that the LUMO is not located at the center of the peak but rather coincides with its early onset at $\sim 300\ \mathrm{meV} - 400\ \mathrm{meV}$. The overall broad peak of the positive resonance is then attributed to further tunneling into other orbitals and electron-phonon coupling[6].

These results are in line with the observed values for $\epsilon + U$ from the exchange bias model.

4. Density Functional Theory (DFT) of FePc

We use DFT to correlate the charge and spin states of the FePc molecule with the charge states of a single impurity Anderson model (SIAM shown in Extended Data Fig. 2a). When adsorbed on 2 ML of MgO, the ground state of FePc has been found to be singly negatively charged, i.e. $[FePc]^{-1}$ and S=1/2[2]. This corresponds to the ground state of the SIAM which is singly occupied. To understand the charge transition points (i.e. ε and $\varepsilon + U$) we perform charge-constrained SCF calculations, often also referred to as ΔSCF calculations[7]. Calculating the DFT total energy of the positively charged $[FePc]^{0}$ (S=1) and doubly negatively charged $[FePc]^{-2}$ (S=0) states allows to estimate the ionization potential (IP) and the electron affinity (EA) of the system. Their difference is the charging energy $U = 2.6\ \mathrm{eV}$ (see Extended Data Fig. 2b), which is in good agreement with the experimental observations (see Tab. S1). We note that this value depends on several DFT parameters such as the van der Waals correction or the value of Hubbard-U.

Interestingly, the change in orbital occupation in these charge transition points indicates the addition or removal of an electron from an orbital with strong ($\sim 71\%$) dz^2 character, that is predominately localized at the Fe center. This is illustrated in the PDOS in Extended Data Fig. 2c and justifies, to a good approximation, the mapping of the charge and spin transitions of the $[FePc]^{-1}$ molecule with a_{1g} character to a single d-like orbital, as it is often considered in the SIAM. It also supports the results of the spatially resolved d*I*/d*V* measurements (see previous section), that the onset of the LUMO is overestimated by taking its peak position: Multiple orbitals occur at a similar energy of $\sim 0.5\ \mathrm{eV}$ (middle panel). Here, the spin-bearing orbital occurs at lower energies.

Details of the DFT calculation: DFT calculations were performed with an FePc molecule placed in a $20\mathrm{x}20\mathrm{x}20$ Angstrom vacuum box. We used plane-wave DFT with pseudopotentials as implemented in Quantum Espresso. Pseudopotentials from the PSL library were used for all atoms and the basis was expanded with a cutoff of 90 $(900)\ \mathrm{Ry}$ for the kinetic energy (charge density). The PBE generalized gradient approximation was used to tread exchange and correlation. We used rVV10 for dispersive forces and Markov-Payne correction to decouple the cells from their periodic image. The relaxed geometry of the singly charged $[FePc]^{-1}$ was used for the ΔSCF calculations and no further relaxations were taken into account (sudden approximation for the charge transitions). The spin ground state was obtained from constrained DFT calculations of all possible spin states. The Brillouin zone was sampled using only the Γ-point and no Hubbard-U correction on the Fe was used in this calculation.

5. Properties of the Fe-FePc Complex on MgO

As described in Ref.[8], the Fe-FePc complex consists of an FePc molecule and an additional Fe atom. They are connected via one of the ligands which covers the Fe adatom to form an arene complex (Fig. S3a). Here, the central Fe atom of the molecule is (2,1) lattice sites apart from the additional Fe atom (See Fig. S3b). Isolated Fe atoms on MgO ($3d^{6.54}s^{0.8}$ configuration[9]) possess a S=2 with strong out-of-plane anisotropy[10]. However, the presence of the benzene ring from the molecule's ligand rearranges the energy levels leading to S=1 via the formation of molecular orbitals. The total spin system can then be described by a S=1 (Fe) coupled to a S=1/2 (FePc):

$$H = J \cdot S_{\mathrm{FePc}} \cdot S_{\mathrm{Fe}} + D \cdot S_{\mathrm{z,Fe}}^2 \tag{S2}$$

With the antiferromagnetic exchange interaction $J = 14\,\mathrm{meV}$ and out-of-plane anisotropy $D = 1.9\,\mathrm{meV}$. The energy levels of this Hamiltonian are shown in Fig. S3c with a ground state doublet with $m_{\mathrm{z}} = \pm\frac{1}{2}$:

$$|0\rangle = \frac{1}{\sqrt{3}}\left|+\frac{1}{2};0\right\rangle - \frac{\sqrt{2}}{\sqrt{3}}\left|-\frac{1}{2};+1\right\rangle \tag{S3}$$

$$|1\rangle = \frac{\sqrt{2}}{\sqrt{3}}\left|+\frac{1}{2};-1\right\rangle - \frac{1}{\sqrt{3}}\left|-\frac{1}{2};0\right\rangle \tag{S4}$$

Leading to an effective S=1/2 two level system of the Fe-FePc complex. Note that the states here are correlated, since they are not separable anymore. The effective S=1/2 possesses a magnetic moment of $\mu = 1.003 \pm 0.025\,\mu_{\mathrm{B}}$ found by electron spin resonance measurements (For more details, see Ref.[8]). Therefore, we use $g = 2$ in our analysis for the ESR condition, see Eq. (1) in the main text.

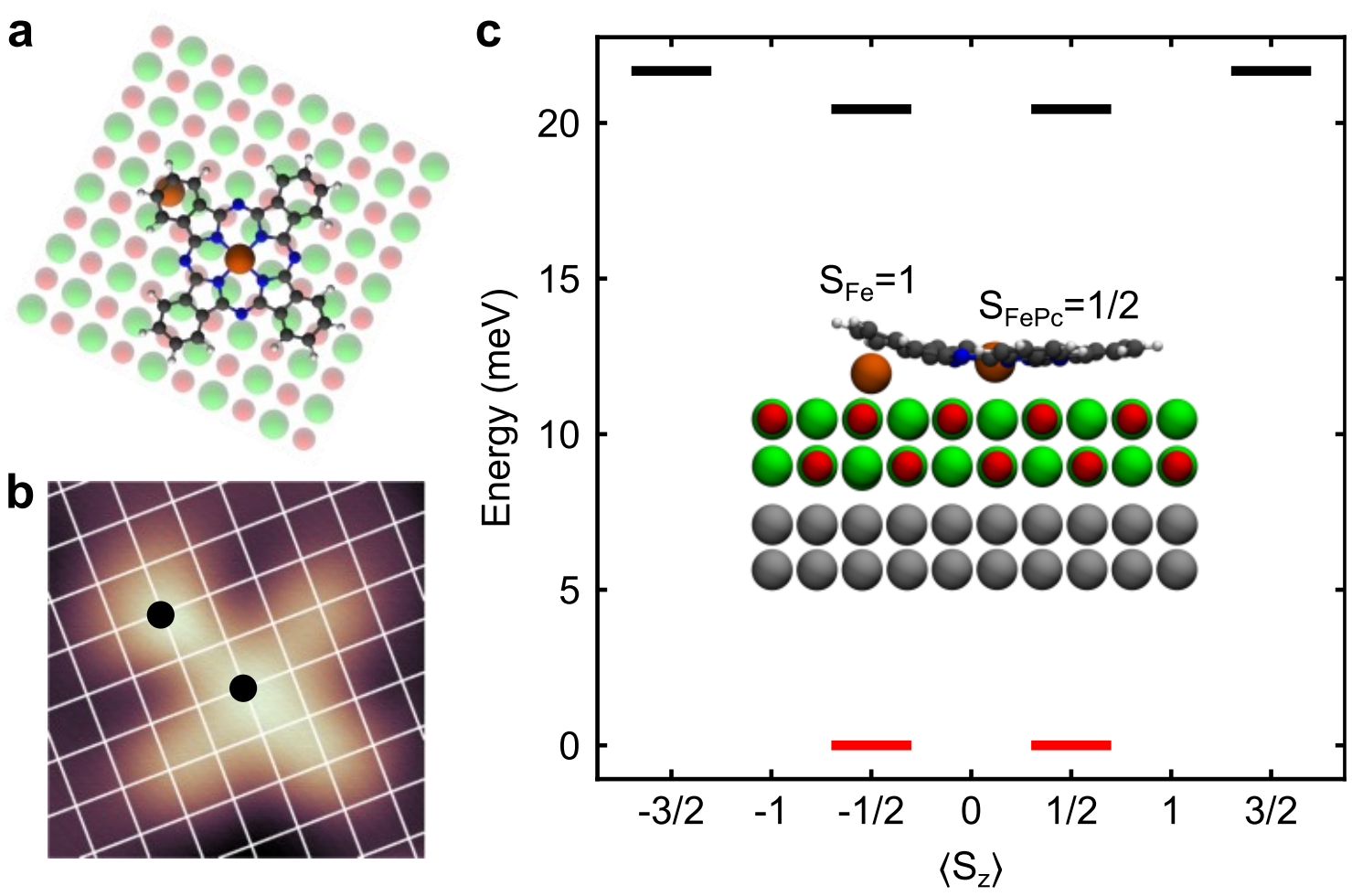

Supplementary Fig. S3. (a) Chemical structure of the Fe-FePc complex adsorbed on the MgO surface. **(b)** Topography of the Fe-FePc complex used for the experiments shown in Fig. 3 of the main text (Image conditions: $I = 10\ \mathrm{pA}, V_{\mathrm{DC}} = -100\ \mathrm{mV}$). The white grid is an overlay of the MgO lattice. The black dots indicate the adsorption sites of the two Fe atoms on the oxygen of the MgO. **(c)** Energy level diagram showing the eigenstates of the Hamiltonian from Eq. (S2) as a function of $\langle S_{\mathrm{z}}\rangle$. The ground state doublet is shown in red. The inserted chemical structure shows a side view of the Fe-FePc complex with the respective spin states.

The Fe-FePc complex offers advantages for coherent spin control, since we achieve higher Rabi rates with less RF voltage compared to pristine FePc. We attribute this to two factors. First, the relaxation time of Fe-FePc is increased compared to pristine FePc. This follows directly from the structure of the ground-state doublet, where electronic correlations reduce the transition matrix element between $|0\rangle$ and $|1\rangle$. As a result, the probability of inelastic scattering with tunneling electrons is suppressed. This allows for longer lifetimes in the measurement or operation at higher readout currents. Although increasing the readout current introduces additional relaxation channels[11], it also improves the signal-to-noise ratio and tip magnetic fields (which improve Rabi rates), which is the strategy adopted here. Second, a less explored contribution may arise from the energetic position of one of the spin-bearing orbitals (Section S8), which lies closer to the Fermi level: It is predicted by theory that such proximity enhances not only the SEC-induced frequency shift (as discussed in this work), but also the Rabi driving strength[12].

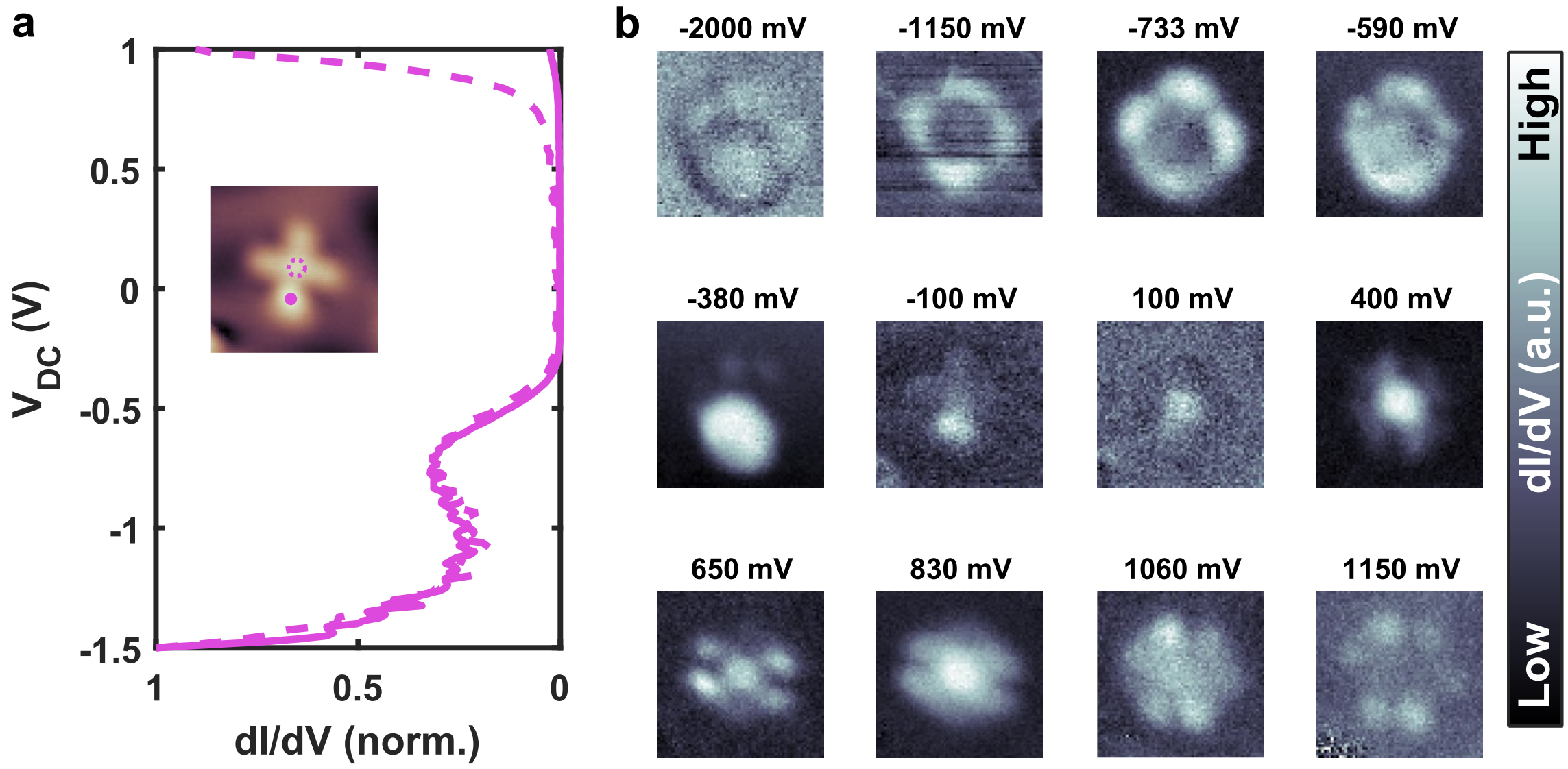

Supplementary Fig. S4. (a) Normalized differential conductance (d*I*/d*V*) spectra acquired on an Fe–FePc complex as the numerical derivative of the V_{DC} dependent tunnel current I ($I_{\mathrm{set}} = 5\ \mathrm{pA}, V_{\mathrm{set}} = 100\ \mathrm{mV}$). The inset shows the measurement positions as colored dots on the topography (Image conditions: $2.5\ \mathrm{nm} \times 2.5\ \mathrm{nm}, I_{\mathrm{set}} = 5\ \mathrm{pA}, V_{\mathrm{set}} = 100\ \mathrm{mV}$). **(b)** Spatially resolved d*I*/d*V* maps of the Fe-FePc complex shown in (a). The signal is recorded in closed loop at the bias voltages V_{DC} via a lock-in modulation V_{mod} ($2.5\ \mathrm{nm} \times 2.5\ \mathrm{nm}$, $I_{\mathrm{set}} = 5\ \mathrm{pA}, V_{\mathrm{mod}} = 10\ \mathrm{mV}$).

Fig. S4a shows d*I*/d*V* spectra obtained on the Fe-FePc complex. In contrast to the pristine FePc, we find the HOMO resonance closer to the Fermi level at $\approx -600\ \mathrm{mV}$ while the LUMO is $>\ 1000\ \mathrm{mV}$. For the LUMO, the signal is more pronounced on the FePc center site compared to the Fe site of the complex. To obtain spatial information we performed d*I*/d*V* maps, visible in Fig. S4b. Similar to the pristine FePc molecule (see Extended Data Fig. 1b), the signal corresponding to the LUMO spreads from the center to the lobes for increasing voltage and is more present on the molecule site. For negative voltages the signal is more located on the Fe adatom which is best visible in the map measured at $-380\ \mathrm{mV}$.

6. Spin-Electric Coupling Models

This section aims to give an overview and discuss the possible mechanisms that lead to SEC for spins on surfaces.

6.1 Piezo-Electric Displacement with g-factor Modulation

As proposed in Ref.[13], a piezoelectric displacement of a surface spin can originate from its local charge interacting with the electric field which is induced by the bias voltage in the tunnel junction. On the one hand this displacement by the electric field could change the g-factor[14]. Such a mechanism would be independent of the direction of the tip field i.e. the tip polarization P. However, as demonstrated by the measurements shown in Fig. 2, the direction of the SEC depends strongly on the tip polarization, where one can achieve a frequency shift in both directions for the same applied bias voltage. Therefore, we conclude that the observed SEC must be dominated by another mechanism, more related to the tip magnetic field orientation.

6.2 Piezo-Electric Displacement with Tip Exchange Field

On the other hand, a piezoelectric displacement in the magnetic field of the tip would lead to a voltage dependent tip field $B_{\text{tip}}(V_{\text{DC}})$. In the following, we discuss the spin-electric coupling (SEC) observed for FePc using a piezoelectric displacement model.

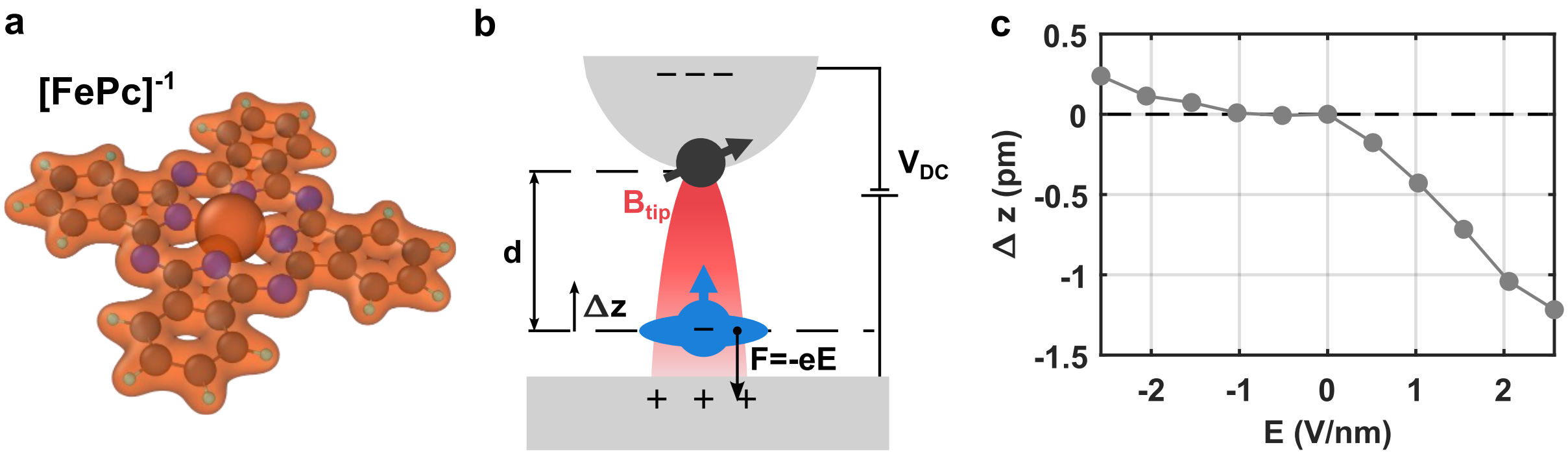

Supplementary Fig. S5. (a) Charge distribution of the $[FePc]^{-1}$ molecule obtained from DFT calculations. The iso-surface corresponding to a charge density of $-5 \times 10^{-4}\ e/a_0^3$ is shown in red. **(b)** Schematic drawing of the STM tunnel junction in the plate capacitor model. In our description, a positive displacement Δz is defined as a displacement towards the tip. For a negatively charged molecule, with charge $-|q|$, a downward (upward) force F is expected for a positive (negative) E. **(c)** Calculated displacement Δz of the center Fe atom of the FePc as a function of electric field. The symmetry is broken here by the surface of the MgO/Ag substrate.

In order to experience a force $\boldsymbol{F}_{\text{el}} = q\boldsymbol{E}$, the on-surface spin is required to have a net charge q. From DFT calculations we find that the charge distribution of the $[FePc]^{-1}$ is homogenously distributed over the center and the ligands (see Fig. S5a). Therefore, all areas experience in good approximation the same direction of displacement by an applied electric field E as a negative point charge in a plate capacitor (also see Ref.[13]). Here, one electrode is given by the tip and the other by the sample (Fig. S5b). In this model, we estimate the electric field as $|\boldsymbol{E}| = V_{\text{DC}}/d$ leading to the force $\boldsymbol{F} = q|\boldsymbol{E}|\hat{\boldsymbol{e}}_z$ perpendicular to the surface. Further, the system can be modelled by a displacement

force $F_{\mathrm{dis}} = -k \cdot \Delta z$. In the equilibrium state $F_{\mathrm{dis}} + F_{\mathrm{el}} = 0$ we then get the displacement as a function of the applied bias voltage $\Delta z = q \cdot V_{\mathrm{DC}}/(d \cdot k)$.

Using DFT calculations we calculated the expected displacement of the $[FePc]^{-1}$ molecule on MgO using Quantum Espresso and employing a plate capacitor model. The result is shown in Fig. S5c plotting the displacement Δz along the positive z-axis, i.e. away from the surface, over the applied electric field strength E along the same direction. As expected, we find negative (positive) Δz for positive (negative) E, i.e. $E \propto -\Delta z$. Thus, Δz and E are anticorrelated.

We compare this first *qualitatively* with our experimental data. The presented dataset in Fig. S6a (same molecule/tip as in Fig. 2b in the main text) shows the shift of the resonance peak Δf as a function of the applied bias voltage V_{DC} measured at different tip-sample distances d. We observe that Δf is positive and increases for a decreasing d. Therefore, the spin must experience a higher magnetic tip field when the tip is moved closer to it which has the same effect as a movement $\Delta z > 0$. Since we also observe an increase of Δf with V_{DC} (i.e. positive E), we also conclude a positive Δz through the electric field, i.e. $E \propto +\Delta z$. This is incompatible with the found anticorrelation between Δz and E from the plate capacitor model and DFT calculations presented above.

Next, we *quantitatively* compare the displacement predicted by our DFT calculations with the experimentally observed magnitude of the SEC. As a first step, we estimate the electric field in the tunnel junction. From point contact measurements we find a tip sample distance of ca. $300 - 400\ \mathrm{pm}$ for the setpoints used in ESR experiments. This is comparable to the values found for Ti and Fe on 2 ML MgO[15]. Thus, for a change in V_{DC} from $50\ \mathrm{mV}$ to $250\ \mathrm{mV}$ follows $E \approx 0.15 \frac{\mathrm{V}}{\mathrm{nm}}$ to $0.75 \frac{\mathrm{V}}{\mathrm{nm}}$. According to Fig. S5c we can expect a displacement of $< 0.5\ \mathrm{pm}$. Therefore, we use $0.5\ \mathrm{pm}$ as an upper bound for the subsequent evaluation.

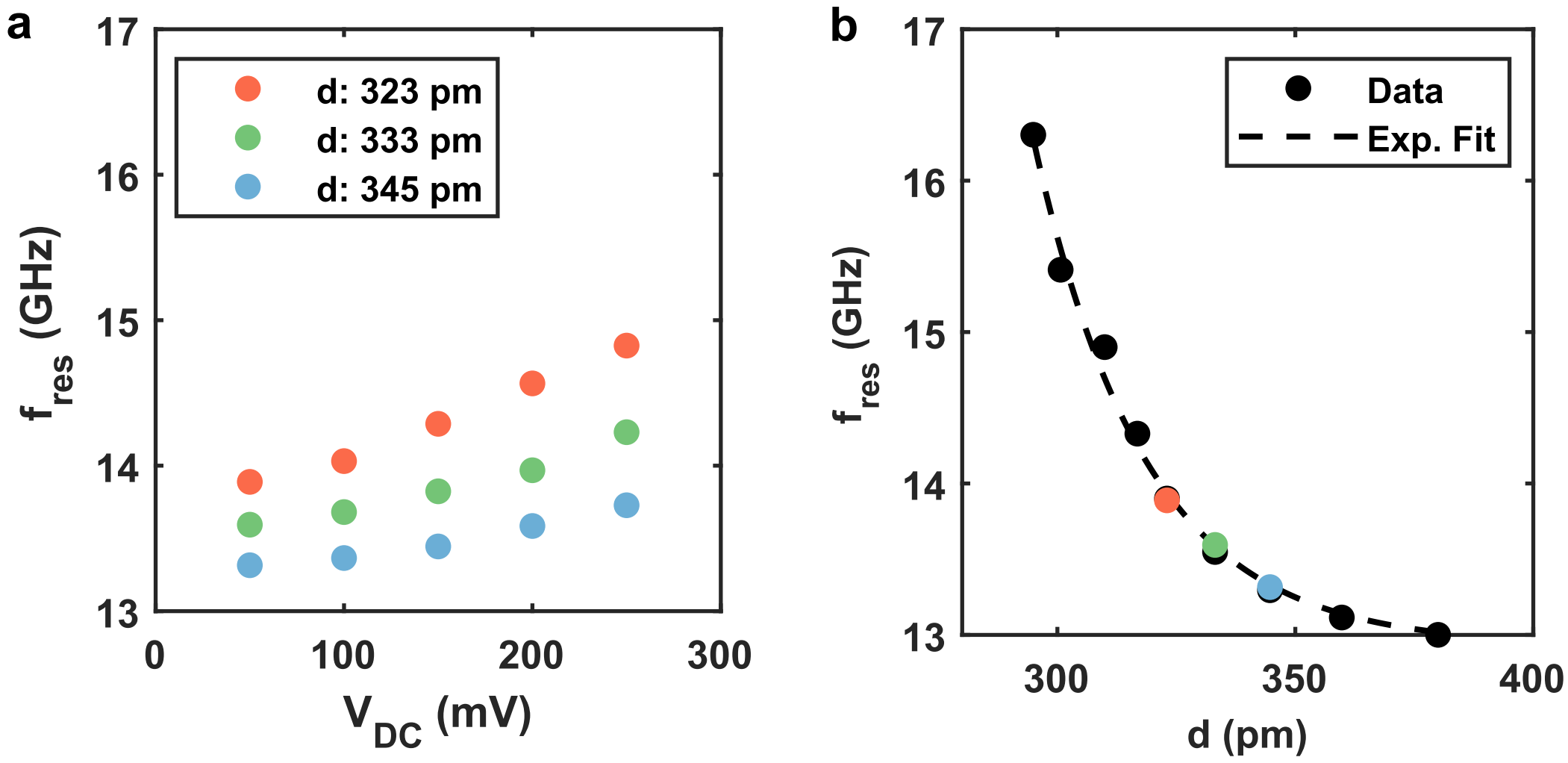

Supplementary Fig. S6. (a) Measured SEC showing the resonance frequency over the applied V_{DC} for different current setpoints corresponding to different tip-sample distances d (ESR conditions: $V_{\mathrm{set}} = 60\ \mathrm{mV}, B = 484\ \mathrm{mT}, V_{\mathrm{RF}} = 10\ \mathrm{mV}$). **(b)** Resonance frequency as a function of tip-sample distance d measured with the same tip as (a) (ESR conditions: $V_{\mathrm{set}} = V_{\mathrm{DC}} = 60\ \mathrm{mV}, B = 484\ \mathrm{mT}, V_{\mathrm{RF}} = 10\ \mathrm{mV}$). The dashed line shows an exponential fit to the data. The coloured dots mark the corresponding setpoint values from (a).

To connect a displacement experimentally with a shift in frequency we analyze setpoint-dependent frequency sweeps. The obtained resonance frequencies are plotted as a function of tip-sample distance d in Fig. S6b. We obtain the expected exponential dependence, which is characteristic of exchange interaction. Using the estimated displacement of $\Delta z = 0.5\ \mathrm{pm}$ and the tip–sample distances of $345, 333$ and $323\ \mathrm{pm}$ (colored points in Fig. S6b), we obtain expected frequency shifts of $43, 56$ and $65\ \mathrm{MHz}$, respectively. In contrast, the corresponding voltage dependent measurements (Fig. S6a) yield $\Delta f = 412, 636$ and $937\ \mathrm{MHz}$ between $50\ \mathrm{mV}$ and $250\ \mathrm{mV}$. With more than one order of magnitude difference, we conclude that the piezo-electric displacement is not responsible for the observed frequency shift. Together with the previously discussed mismatch of the displacement direction we find that a different mechanism must be responsible for the large SEC effect observed in the measurements.

6.3 The Exchange Bias Model

As discussed in the main text, the theory of the exchange bias model, that we use to model our data, has been originally established in the framework of DC transport using magnetic electrodes in quantum dots[16–20]. In more recent theoretical works by some of the authors this has been adapted for the framework of STM and single atoms and molecules[12,21,22].

Single Impurity Anderson Model: As discussed in Section S4, spins in these systems can be well described by a single impurity Anderson model (SIAM)[23,24]. As illustrated by the density of states in Extended Data Fig. 4a, the single occupied orbital lies below the Fermi Energy E_{F} with the ionization energy ϵ. Since the singly charged state is the ground state, ϵ needs to be negative, $\epsilon < 0$. The doubly occupied orbital

appears at positive energy. The energy difference between the orbitals is given by the Coulomb repulsion energy U. In our model, the chemical potential of the molecule aligns with the substrate electrode due to the adsorption on the MgO surface.

Virtual Tunneling: The SIAM is used to describe the virtual particle exchange between the impurity and the spin baths from the tip and surface. Extended Data Fig. 4b shows a schematic drawing of the tunnel junction. The energy levels from the SIAM are situated between the spin-polarized tip electrode (left) and the non-magnetic sample electrode (right), separated by two tunneling barriers with vacuum on the tip side and a thin MgO layer on the sample side. We consider a first-order virtual tunneling process between the tip electrode and the impurity. During this process, an electron tunnels into or out of the impurity and then back. Because the tip is spin-polarized, the probabilities for these virtual transitions differ for spin-up and spin-down state of the molecule. By applying a bias voltage V_{DC} we shift the chemical potential of the tip relative to the molecule, thereby tuning the rates of virtual electron addition (positive bias) or removal (negative bias).

Exchange Field: The virtual tunneling results in spin-dependent energy corrections to the molecule's spin states, modifying the effective energy splitting beyond the contribution of the external magnetic field B. An exemplary energy level diagram for $P > 0$ is shown in Extended Data Fig. 4c. The corrections can be described by an effective exchange field as presented in Eq. (2) in the main text. As shown in Extended Data Fig. 4d, this leads to a linear shift of the resonance frequency f_{res} for small bias voltage V_{DC} and logarithmic divergences near the impurity energy levels ϵ and $\epsilon + U$. To arrive at the expression given in Eq. (2) we further add a residual tip field B_0 which accounts for additional effects not covered by the transport model. One main contributor to this is the magnetic dipole field from the tip. To give a precise value of this contribution remains difficult since the magnetic moment of the tip and its angle is not known. However, for a tip-sample distance of $300 - 400\ \mathrm{pm}$, (obtained from point contact measurements) and assuming $\mu_{\mathrm{tip}} = 1 - 10\ \mu_{\mathrm{B}}$[15,25,26], we can expect contributions in the order of 10 to $100\ \mathrm{mT}$. Therefore, the residual fields observed in the dataset of Fig. 2 fall well within this range. Finally, we note that in Eq. (2) we use the convention that for a positive field $B > 0$, the up state of the electron spin is the ground state.

ESR Driving: The exchange-bias model has also been proposed as the driving mechanism for ESR-STM (see Ref.[12]). In this picture, the fraction of the tip's spin polarization that is oriented perpendicular to the quantization axis of the surface spin, produces - under the influence of the RF voltage - an oscillating exchange field that drives the spin when the RF frequency matches the resonance condition. Thus, to realize the proposed SEC through the DC voltage and the driving via the RF voltage simultaneously, the tip must be partially polarized parallel and perpendicular to the quantization axis (i.e. external magnetic field). In this analysis, only the parallel

component of the exchange field shifts the resonance frequency. For the sake of simplicity, we include the θ dependence in Ref.[22] into the tip's coupling γ_{T} (Eq. (2)), although the angle dependence is explicitly taken into account in the full transport simulations (see Tab. S1).

7. Linear Spin-Electric Coupling (SEC) of FePc and Fe-FePc

In this section, we investigate the linear regime of the spin-electric coupling (SEC) for FePc and Fe-FePc in greater detail. According to Eq. (2) the SEC depends on the magnetic tip electrode. This can greatly vary depending on the precise microtip[15,25] used in the STM experiment. Therefore, we show here statistical data for different tips and spin centers and for different setpoints.

We perform a Taylor expansion of Eq. (2) in the main text and find a linear approximation of the exchange field:

$$B_{\mathrm{tip}} \approx B_0 + \frac{P\gamma_{\mathrm{T}}}{2\pi}\left(\ln\left(\left|\frac{\epsilon}{\epsilon+U}\right|\right) + \left(\frac{1}{\epsilon} - \frac{1}{\epsilon+U}\right)\cdot V_{\mathrm{DC}}\right) \tag{S6}$$

To quantify the SEC we performed a linear fit to the peak shift $\Delta f = f_{\mathrm{res}} - f_0$ over V_{DC}:

$$\Delta f = m \cdot V_{\mathrm{DC}} + c \tag{S7}$$

$$c = \frac{g\mu_{\mathrm{B}}}{h}\left(B_0 - \frac{P\gamma_{\mathrm{T}}}{2\pi}\ln\left(\left|\frac{\epsilon}{\epsilon+U}\right|\right)\right) \tag{S8}$$

$$m = \frac{g\mu_{\mathrm{B}}}{h}\frac{P\gamma_{\mathrm{T}}}{2\pi}\left(\frac{1}{\epsilon} - \frac{1}{\epsilon+U}\right) \tag{S9}$$

For the offset c we identify two contributions. The first one comes from the residual tip field B_0. The second arises from the exchange bias model and reflects properties of the tip and the orbital structure of the molecule. The slope m captures the magnitude of the SEC and the direction of the frequency shift with voltage via its sign. It scales linear with the polarization P and the coupling between tip and molecule γ_T. Further, its magnitude increases when the orbitals are closer to the Fermi level. Its sign solely depends on the sign of the tip polarization. In contrast, the sign of c also depends on whether the HOMO or LUMO is closer to the Fermi level. Therefore, the exchange bias model predicts a correlation between m and c for the LUMO closer to E_{F} (as for the pristine FePc, $|\epsilon| > |\epsilon + U|$) and an anticorrelation for the HOMO closer to it (as for the Fe-FePc complex, $|\epsilon| < |\epsilon + U|$). These connections are independent of P and γ_T and thereby form a measure reflecting the spin orbital structure.

To examine this behavior, we measured the SEC in the linear regime for various molecules and tips. Fig. S7a provides an overview of the extracted m over the setpoint conductance G (corresponding to different tip-sample distances). m varies for different tips, with extracted values ranging from $0.44\ \mathrm{MHz/mV}$ to $8.22\ \mathrm{MHz/mV}$.

For the same tip and location, higher conductance values G, corresponding to smaller tip-sample distances, lead mostly to an enhanced SEC $|m|$. This is consistent with the expectation from a larger tip-molecule coupling γ_{T} since both quantities increase with

a smaller distance between tip and molecule. Different values obtained for the same molecule type are attributed to different polarizations P and γ_{T} of each tip. We note here that in our description γ_{T} includes the angle θ between the polarization axis and the quantization axis of the spin. Differences for the same tip with varying molecule sites could stem from the different orbital configuration but also from different couplings γ_{T}: Although the setpoint conductance fixes the vertical tip position, the molecular structures vary in height across the surface. As a result, γ_{T} can be different for the three different locations. This connection between tip proximity and magnetic coupling has been demonstrated previously in Ref.[15]. Additionally, we find a tendency for the Fe-FePc complex (center and Fe-site) to exhibit positive m ($P < 0$) whereas FePc tends to show negative m ($P > 0$).

Fig. S7b shows the extracted offset c over the conductance G, which shows an increase of $|c| \propto G$, despite a general scattering of the values that we again attribute to the different tip configurations. In Fig. S7c,d we analyze the correlation of m and c for FePc and Fe-FePc by plotting the offset c over the slope m. We quantify their correlation via their r coefficient[27]. For the FePc we find $r = 0.67$ and a p-value of $p = 0.005$. For Fe-FePc we find a weak but present anticorrelation with $r = -0.45, p = 0.045$. These correlations are in line with the discussion above concerning Eq. (S7)-(S9) on the dependence of the orbital structure and SEC in the linear regime.

In summary, although the results scatter for different tips and molecules, all measurements yield a SEC on the order of $m \approx 1\ \mathrm{MHz/mV}$. Moreover, the data clearly reveals a trend of increasing SEC with larger setpoint conductance G, which we attribute to the enhanced coupling γ_{T}. Finally, the observed correlation between m and c agrees well with the predictions from the exchange bias model and the SIAM for the respective molecules.

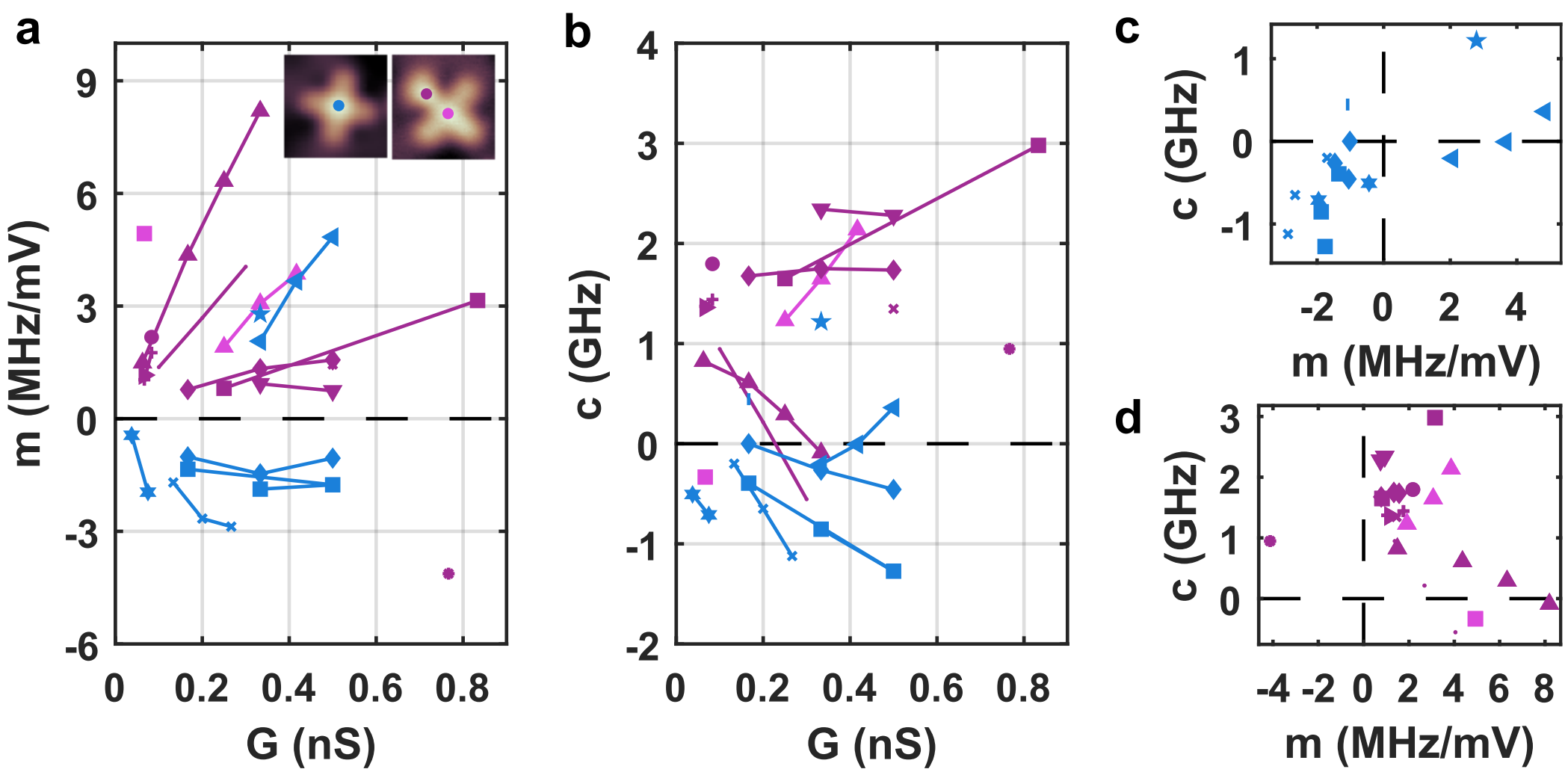

Supplementary Fig. S7. (a) Extracted slope m of the resonance frequency shift $\Delta f = m \cdot V_{\mathrm{DC}} + c$ in the linear regime plotted over the setpoint conductance G of the respective measurement. Different symbols correspond to different tips. Connected data points represent measurements at various G with the same tip. Blue: measurements on the FePc center. Pink: measurements on the Fe-FePc center. Purple: measurements on the Fe-site of the Fe-FePc. The measurement positions are also indicated in the inserted topographies of the respective molecules. **(b)** Accordingly, the plot shows the obtained offset c over the setpoint conducntace G. **(c)** c as a function of m for the measurements on pristine FePc. The data shows a correlation between the two values with $r = 0.67, p = 0.005$. **(d)** c as a function of m for the Fe-FePc complex with a weak anticorrelation: $r = -0.45, p = 0.045$.

8. Polarity Preference of FePc and Fe-FePc

Studying the SEC, we found a strong dependence of the ESR amplitude on the bias polarity. For FePc, the ESR signal is typically stronger for positive V_{DC} (Fig. S8a), whereas the Fe-FePc complex generally shows a more prominent signal for negative V_{DC} (Fig. S8b or Extended Data Fig. 6). This polarity-dependent trend was consistently observed for all ESR-active tips. Within the framework of the exchange bias model, this effect can be caused by an enhanced Rabi rate close to the energy levels of the SIAM[21,22]. Since the two orbitals are not equally distant from the Fermi energy, we expect an enhanced Rabi rate for the FePc (Fe-FePc) at positive (negative) V_{DC}. This results in an enhanced ESR amplitude for the respective voltage polarity.

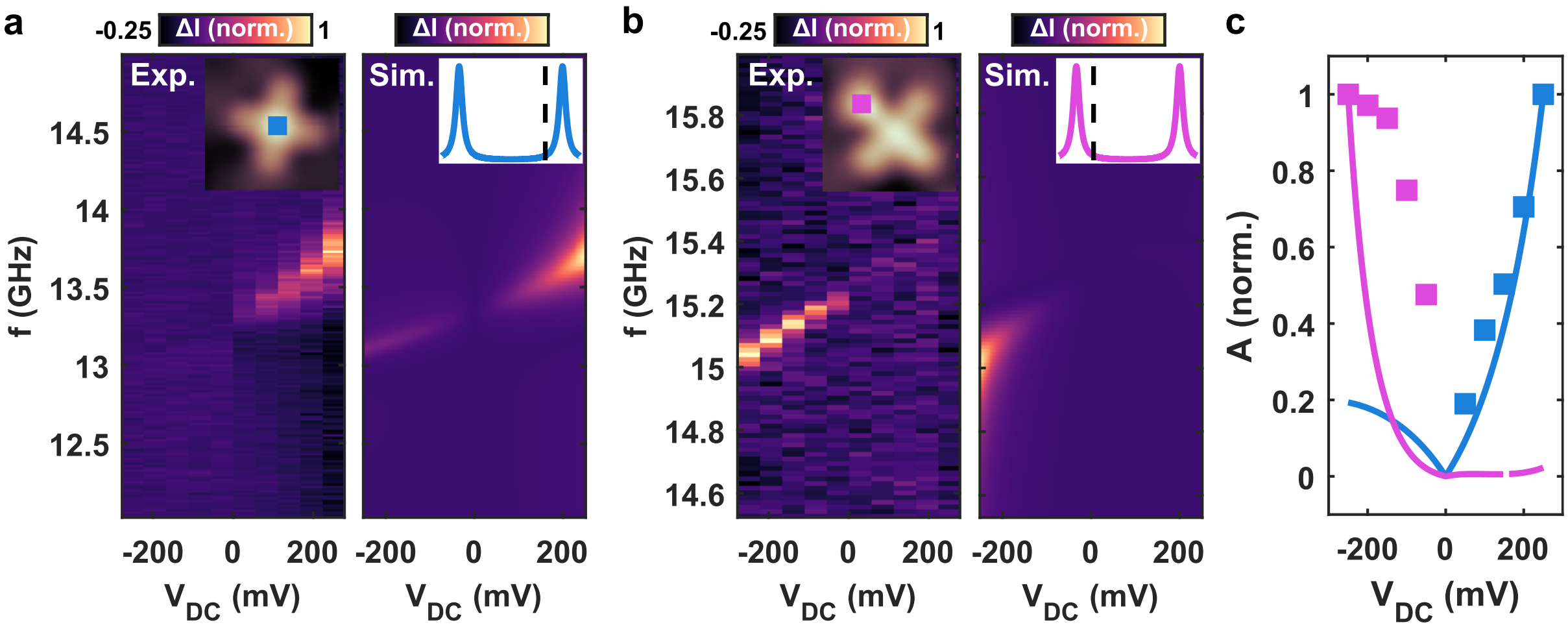

Supplementary Fig. S8. (a) Left: ESR frequency sweeps on FePc (see inserted topography) for different bias voltages plotting ΔI as a function of f and V_{DC} (ESR conditions: $I_{\mathrm{set}} = 5\ \mathrm{pA}, V_{\mathrm{set}} = 100\ \mathrm{mV}, B = 484\ \mathrm{mT}, V_{\mathrm{RF}} = 10\ \mathrm{mV}$). Right: Corresponding simulation based on the transport model (Simulation parameters: $\epsilon = -2000\ \mathrm{meV}, U = 2500\ \mathrm{meV}, P = 0.7, \gamma_{\mathrm{T}} = 15\ \mathrm{\mu eV}, \gamma_{\mathrm{S}} = 10\ \mathrm{\mu eV}, T = 1\ \mathrm{K}, \Delta = 20\ \mathrm{meV}, B = 455\ \mathrm{mT}, \theta = 15°, g = 2$). Both colormaps were normalized to the maximum value. The inset on the top right shows a schematic plot of the SIAM's spectral function with the doubly occupied orbital close to the Fermi level (dotted line). **(b)** Left: Measurements on the Fe-FePc complex (see inserted topography) showing ΔI as a function of f and V_{DC} (ESR conditions: $I_{\mathrm{set}} = 10\ \mathrm{pA}, V_{\mathrm{set}} = -60\ \mathrm{mV}, B = 484\ \mathrm{mT}, V_{\mathrm{RF}} = 8\ \mathrm{mV}$). Right: Corresponding simulation using the electronic properties of the Fe-FePc complex (simulation parameters: $\epsilon = -350\ \mathrm{meV}, U = 1000\ \mathrm{meV}, P = 0.7, \gamma_{\mathrm{T}} = 5\ \mathrm{\mu eV}, \gamma_{\mathrm{S}} = 10\ \mathrm{\mu eV}, T = 1\ \mathrm{K}, \Delta = 20\ \mathrm{meV}, B = 549\ \mathrm{mT}, \theta = 15°, g = 2$). Both colormaps were normalized to the maximum value. The inset on the top right shows a schematic plot of the SIAM's spectral function with the single unoccupied orbital close to the Fermi level. **(c)** Extracted ESR amplitudes A as a function of V_{DC}. The values obtained from the experiment are shown as squares (blue for FePc, purple for Fe–FePc), and simulation results are plotted as solid lines.

We reproduce the two experimental datasets presented in Fig. S8 quantitatively by simulations based on the exchange bias[12,22] using the code provided by Ref.[28]. We set the chemical potential of the sample to zero. Further, we fixed the temperature, g-factor, the broadening of the orbital and the modulation of the tunneling matrix element: $T = 1\ \mathrm{K}, g = 2, \Delta = 20\ \mathrm{meV}, A_{\mathrm{T}} = 50\%$. The ionization energy and Coulomb repulsion energy are chosen to match the orbital structure of FePc (Fig. S8a, right: $U = 2500\ \mathrm{meV}, \epsilon = -2000\ \mathrm{meV}$) and Fe-FePc (Fig. S8b, right: $U = 1000\ \mathrm{meV}, \epsilon = 350\ \mathrm{meV}$). To match the experiment we adapted the magnetic field B, the angle between the polarization axis and the external magnetic field θ, the tip polarization P and the coupling to the substrate (tip) γ_{S} (γ_{T}) as stated in the description of Fig. S8a,b. Fig. S8c shows a plot of the extracted amplitudes $A(V_{\mathrm{DC}})$ from both experiment and simulations. We find a qualitative agreement between experiment and simulations. For a more quantitative explanation, we believe that additional effects need to be considered, such as spin-pumping.

9. Non-linear Spin-Electric Coupling of FePc

In this section we provide further information on the non-linearity of the SEC on FePc which is observed in ESR frequency sweeps. Extended Data Fig. 3 shows a selection of the data of Fig. 2a and b in the main text as raw data waterfall plots. The plots capture the drastic increase of the resonance amplitude at high V_{DC} together with the non-linear shift of the resonance position. In the case of Extended Data Fig. 3a, an additional increased asymmetry of the peak line shape is observed. In both cases, we also see an increased noise level and additional features stemming from the mismatches in the transfer function for high V_{DC} due to the large currents. However, in all cases the fit to a Fano Lorentzian still shows good agreement.

As mentioned in the main text we fitted the experimentally determined resonance frequencies to the exchange bias model described by Eq. (2) (see Extended Data Fig. 5): As measurements were only performed at positive bias voltages, we fixed $\epsilon = -2000\,\mathrm{meV}$, consistent with the value obtained from dI/dV measurements. To incorporate all pre- factors of B_{tip} into the coupling term γ_{T}, we fixed the magnitude of the tip spin polarization to $|P| = 1$. Notably, γ_{T} contains the angle θ between polarization axis and the quantization axis of the surface spin which is used in the simulations later. Additionally, we set the g-factor to $g = 2$, attributing any deviation from the exchange bias model to the residual magnetic field B_0. For the dataset shown in Fig. 2a (red), we find the following fit parameters: $P = 1, \gamma_{\mathrm{T}} = 100 \pm 9\ \mathrm{mT},\ \epsilon + U = 553 \pm 6\ \mathrm{meV}, B_0 = 0.1 \pm 2.7\ \mathrm{mT}$. And for Fig. 2b (blue): $P = -1, \gamma_{\mathrm{T}} = 225 \pm 24\ \mathrm{mT},\ \epsilon + U = 477 \pm 10\ \mathrm{meV}, B_0 = -57 \pm 7\ \mathrm{mT}$. Therefore, we find a higher coupling to the tip γ_{T} for the blue dataset as expected from the higher setpoint conductance used. Although, this could also be explained with a higher tip-polarization. For the blue dataset we find a quite substantial residual tip field B_0 which hints towards further contributions from other mechanisms e.g. magnetic dipole-dipole interaction.

Discussion on the Orbital Energies $\boldsymbol{\epsilon}$ and $\boldsymbol{\epsilon + U}$: We note that the extracted values of $\varepsilon + U$ differ between the two spin polarizations. Within the transport model, such a dependence on spin polarization is not expected. We rather attribute the difference to different tunneling setpoints as well as different molecules in the two measurements, that we believe can slightly shift the orbital energies as observed in the measurements presented in Section S3.

A more substantial difference is observed when comparing the values obtained from the exchange-bias model ($\varepsilon + U \approx 500\,\mathrm{meV}$) with those extracted from the conductance peak in the dI/dV ($E_{\mathrm{L}} \approx 1000\,\mathrm{meV}$). We explain this difference from the fact that the dI/dV peak does not contain a single orbital but a superposition of several unoccupied molecular states: Our DFT calculations (Section S4) show that in the relevant energy range ($\approx\ 300 - 1000\,\mathrm{meV}$) multiple unoccupied orbitals contribute. The spin-bearing dz^2-like orbital appears first, followed by other d-orbitals and ligand states. This is supported experimentally by spatially resolved dI/dV maps (Extended Data Fig. 1b): the onset of the unoccupied-state signal appears on the central Fe site

already around $300\ \mathrm{meV}$ and ligand states start to occur at around $500\ \mathrm{meV}$. Thus, the full dI/dV peak is shifted upward due to contributions from these additional orbitals. Further effects may also contribute to the upshift of the dI/dV peak, including vibronic excitations which were reported for related molecular systems[6] but not resolved here and the differences in tunneling setpoints between dI/dV and ESR-STM measurements, which could modify both electrostatic and force interactions in the junction. Importantly, the fitted values of $\approx 500\ \mathrm{meV}$ lie well within the broad LUMO feature.

Transport Simulation: For the full simulation of the ESR signal we used the obtained energy levels ϵ and $\epsilon + U$ from the previous fit to Eq. (2). We found best agreement with the experiment for the simulation parameters presented in Tab. S1 where we focused on reproducing $f_{\mathrm{res}}(V_{\mathrm{DC}})$. The result is shown in the panels of Extended Data Fig. 5. We find a very good agreement between experiment and the model for the resonance frequency f_0 and amplitude A. Further, the simulation qualitatively captures the observed trend in linewidth Γ of the resonance peaks. However, quantitative discrepancies remain, which are most prominent in the red dataset. Additionally, the evolution of the Fano asymmetry parameter q is not well described by the model. The discrepancies found can be explained primarily by the increase in conductance as V_{DC} approaches the prominent peak obtained in the dI/dV measurements: The resulting non-linear change of the tunnel current leads to a rapid increase of A and Γ and a change of q. This conductance change is not included in the simulation. Further contributions such as spin-pumping are also possible. Despite these limitations, the comparison proves the ability of the exchange bias model in describing the spin-electric coupling of single spins on surfaces.

	B	θ	P	γ_{S}	γ_{T}	U
Red	$589\ \mathrm{mT}$	$17.5°$	-0.66	$17\ \mu\mathrm{eV}$	$19\ \mu\mathrm{eV}$	$2553\ \mathrm{meV}$
Blue	$337\ \mathrm{mT}$	$14.5°$	0.72	$32\ \mu\mathrm{eV}$	$36\ \mu\mathrm{eV}$	$2477\ \mathrm{meV}$

Supplementary Tab. S1. Simulation parameters of the colormaps presented in Fig. 2. With the magnetic Field B, the angle between the polarization axis and the external magnetic field θ (see Section S6.3) the tip polarization P, the coupling to the substrate (tip) γ_{S} (γ_{T}) and the already introduced Coulomb repulsion energy U. For both simulations we fixed: $T = 1\ \mathrm{K}, g = 2, \Delta = 20\ \mathrm{meV}, A_{\mathrm{T}} = 50\ \%, \epsilon = 2000\ \mathrm{meV}$.

Setpoint Dependence: Besides the demonstrated dependence on the tip polarization P, the magnitude of the exchange bias also depends on the coupling between tip and molecule γ_{T}, which can be altered by varying the thickness of the vacuum tunnel barrier. This is achieved by varying the conductance setpoint G with the relation $\gamma_{\mathrm{T}} \propto G$. As shown in Fig. S9, the magnitude of the SEC increases for positive and negative polarizations with increasing G.

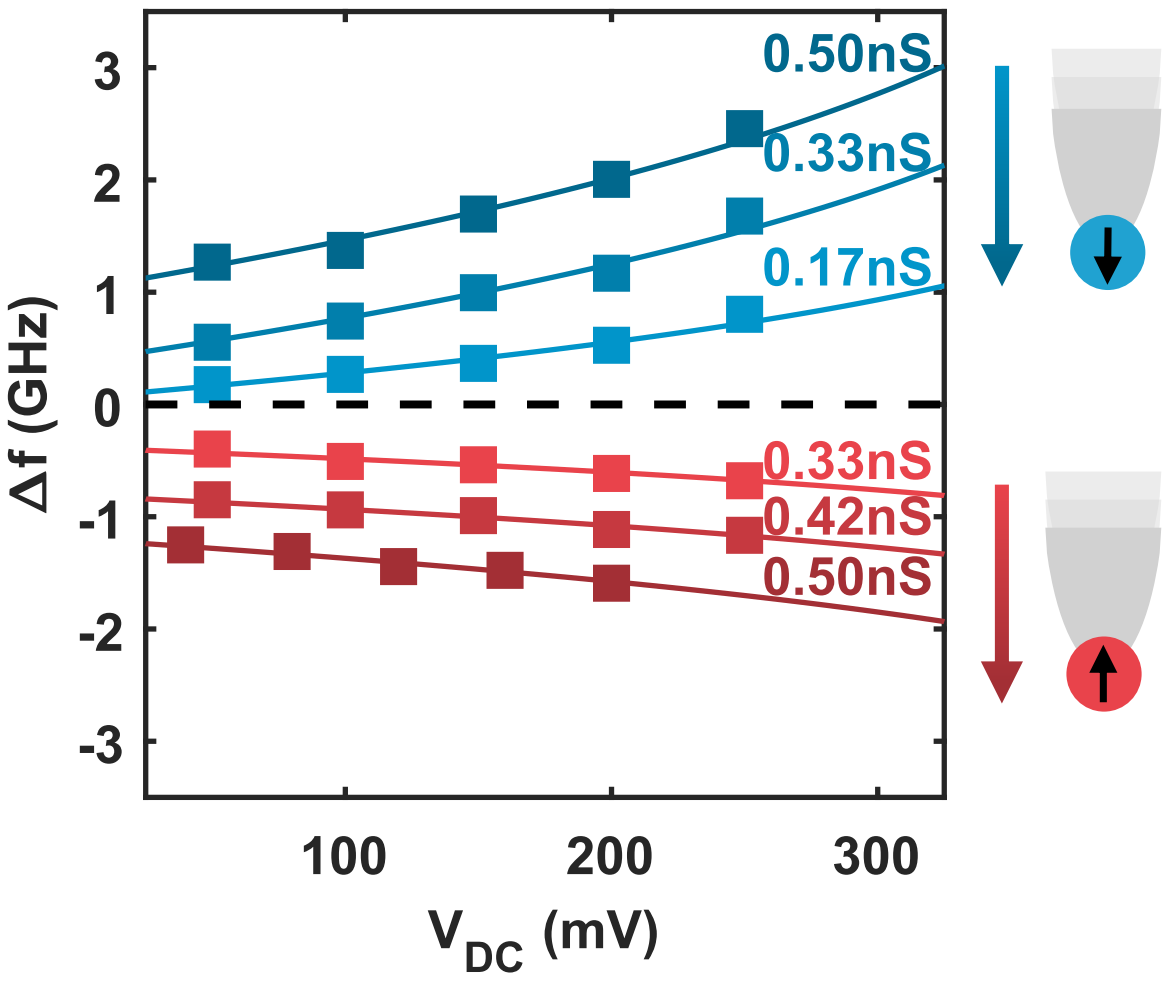

Supplementary Fig. S9. Frequency shift $\Delta f(V_{\mathrm{DC}})$ for different setpoint conductance G (ESR conditions, red: $V_{\mathrm{set}} = 60\ \mathrm{mV}, B = 500\ \mathrm{mT}, V_{\mathrm{RF}} = 10\ \mathrm{mV}$; blue: $V_{\mathrm{set}} = 60\ \mathrm{mV}, B = 383\ \mathrm{mT}, V_{\mathrm{RF}} = 10\ \mathrm{mV}$). The solid lines show fits to the data according to the exchange bias model. The measurements allow the assignment of the direction of B_{tip}: The magnitude of Δf increases with G since $B_{\mathrm{tip}} \propto G$ in good approximation[26,29] and the sign of B_{tip} can according to Eq. (1) be interpreted as opposite tip polarities (see sketches).

External Magnetic Field: In Fig. S10 we show the magnetic field dependence of the resonance peak in the non-linear regime for three magnetic fields: $450\ \mathrm{mT}, 518\ \mathrm{mT}, 585\ \mathrm{mT}$. The color plots in Fig. S10a and the extracted resonance frequencies in Fig. S10b show a clear shift of the feature with magnetic field attributing it to the spin resonance of the surface spin. The data was obtained on the same molecule as Fig. 2a but with a different tip.

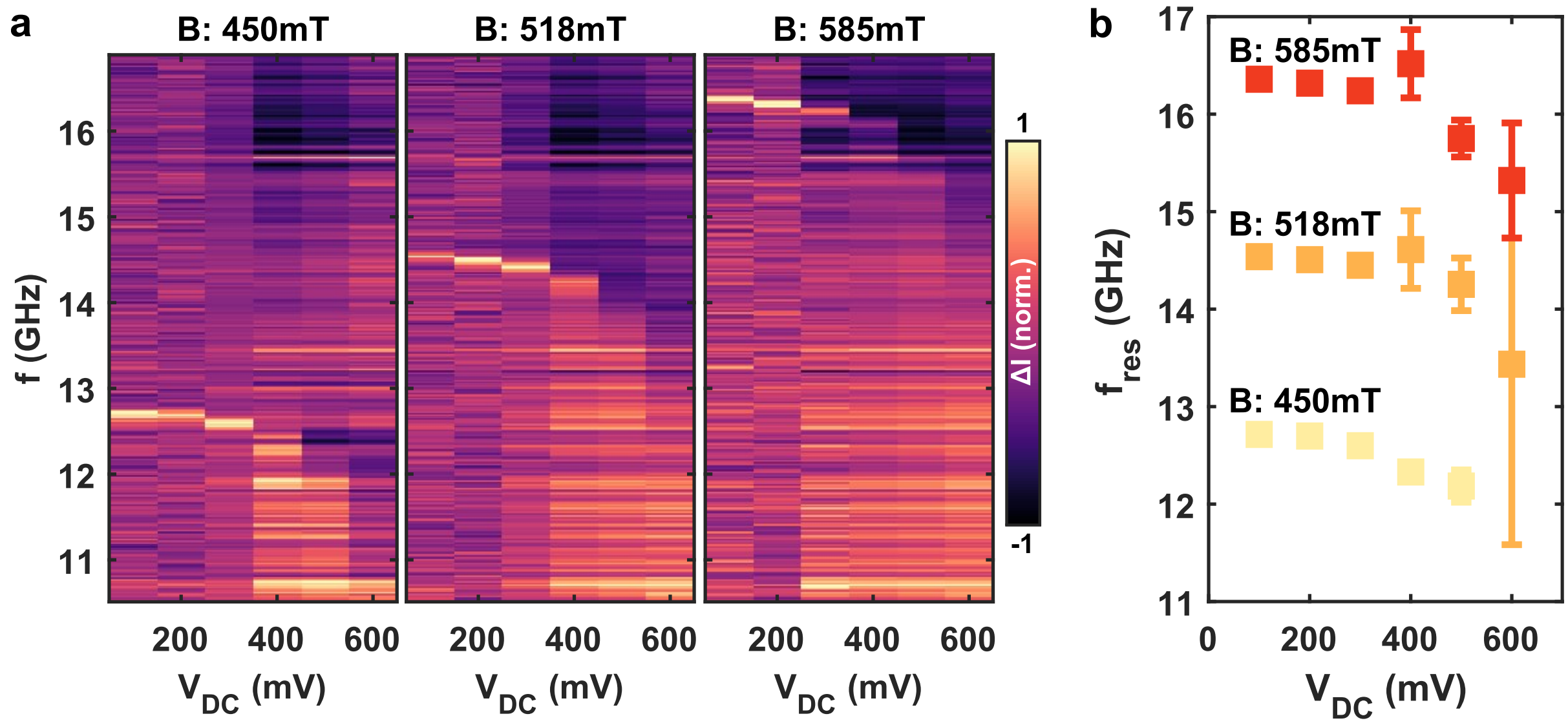

Supplementary Fig. S10. (a) Colormaps of ΔI as a function of V_{DC} and f on FePc for different external magnetic fields B (ESR conditions: $I_{\mathrm{set}} = 4\ \mathrm{pA}, V_{\mathrm{set}} = 60\ \mathrm{mV}, V_{\mathrm{RF}} = \ 12\ \mathrm{mV}$). For better visibility each frequency sweep is normalized separately. **(b)** Extracted resonance frequencies f_{res} from (a) as a function of V_{DC}.

10. Coherent Control Experiments

In addition, to gain a deeper understanding of the Rabi detuning measurements via either frequency or voltage, we provide a more detailed analysis in the following.

Fig. S11a shows a waterfall plot of the Rabi oscillations which were detuned by changing the frequency $\delta f = f - f_{\mathrm{res}}$, shown in Fig. 3c. Each trace was fitted according to Eq. (3) in the main text. As can be seen from Fig. S11b,c the extracted Rabi rate Ω and amplitude A match the expected behaviors from Eq. (4) in the main text. In Fig. S11d we use the fitted datasets in b,c to simulate the observed experimental data, showing good agreement. T_2 and ϕ were chosen according to the mean values obtained from the experiment.

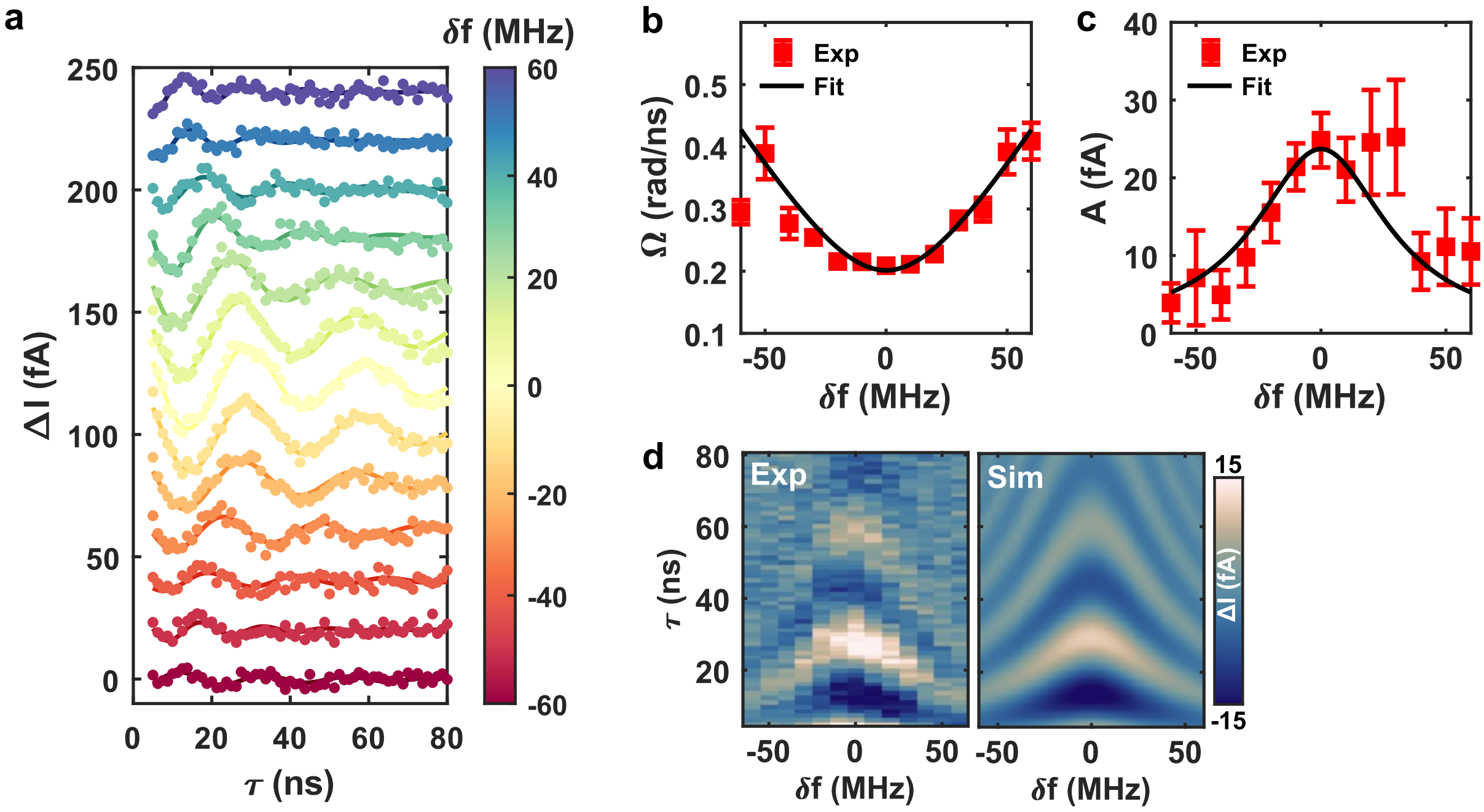

Supplementary Fig. S11. (a) Waterfall plot of Rabi oscillation measurements, corresponding to Fig. 3c in the main text, recorded at various frequency detuning δf. The traces showing ΔI over τ are vertically shifted for clarity. Experimental data points are shown as circles, and the solid lines represent fits to Eq. (3). **(b)** Extracted Rabi rates Ω as a function of δf, obtained from the fits in panel (a). The solid curve is a fit to Eq. (4) in the main text, yielding an intrinsic Rabi rate of $\Omega_0 = 0.201 \pm 0.006\ \mathrm{rad/ns}$. **(c)** Extracted amplitudes A from panel (a) with a fit to Eq. (4), yielding $A_0 = 23.7 \pm 1.6\ \mathrm{fA}$. **(d)** Left: Colormap of the measurements from (a) showing ΔI as a function of τ and δf. Right: Simulated chevron pattern using Eq. (3) and (4) with $A_0 = 23.7\ \mathrm{fA}, \Omega_0 = 0.2\ \mathrm{rad/ns}, T_2 = 27.6\ \mathrm{ns}, \phi = 12.9°$.

Fig. S12a shows the same analysis for the voltage-detuned Rabi oscillations displayed in Fig. 3d in the main text (displayed again in Fig. S12d). We observe again a chevron pattern, demonstrating the detuning through the SEC. Further, the pattern appears slightly distorted. This is reflected in the extracted Rabi rates Ω and amplitudes A (see Fig. S12b,c). We explain this feature via an additional modulation of Ω_0 with V_{DC}, which is predicted by the exchange bias model (see Ref.[21,22]). In first approximation, we consider a linear contribution:

$$\Omega = \sqrt{\Omega_0^2 + \alpha^2 (V_{\mathrm{DC}} - V_{\mathrm{set}})^2},\ \ \Omega_0 = \Omega_{00} + \beta (V_{\mathrm{DC}} - V_{\mathrm{set}}) \qquad \text{(S10)}$$

Here, α is parameterizing the frequency shift with voltage detuning $\delta V = (V_{\mathrm{DC}} - V_{\mathrm{set}})$ from the setpoint voltage. Ω_{00} is the intrinsic Rabi rate at V_{set} and β the linear factor accounting for the additional dependence of Ω_0 on V_{DC}. By fitting the combined formula to the experimental data in Fig. S12b we find the best agreement for $\Omega_{00} = 0.34 \pm 0.02\ \mathrm{rad/ns}, \alpha = 2.1 \pm 0.4\ \mathrm{MHz/mV}, \beta = -0.6 \pm 0.2\ \mathrm{MHz/mV}$. Comparing α to the extracted magnitudes of the SEC m from Section S7 we find that it nicely fits into the observed range. Also, β lies in the same order of magnitude. The negative sign means a stronger increase of Ω with negative δV compared to positive δV which as it is observed in the extracted data. This is expected from the ϵ and U values extracted for

the Fe-FePc complex, since we have the HOMO closer to the Fermi level as the LUMO and therefore expect a stronger virtual tunneling for negative V_{DC}.

For the amplitude A we further assume a scaling with the tunnel current in the junction. We result with the following expression:

$$A = A_0 \frac{\Omega_0^2}{\Omega^2} \cdot \left|\frac{V_{\mathrm{DC}}}{V_{\mathrm{set}}}\right| \tag{S11}$$

With the amplitude at the setpoint voltage A_0. A fit to the experimental data is shown in Fig. S12c finding $A_0 = 21.8 \pm 3.3\ \mathrm{fA}$. Generally, we find a good description of the experiment by this formula with some deviations for $\delta V = -20\ \mathrm{mV}$ and $-25\ \mathrm{mV}$ which most likely stems from a bad fit to the strongly suppressed Rabi oscillations. In Fig. S12b,c we additionally plot fits without the additional correction, i.e. $\beta = 0$. While the agreement is still reasonable, it is not as good as in the case of frequency sweep (Fig. S11) or with the inclusion of a change in $\Omega \propto V_{\mathrm{DC}}$. We interpret these results as evidence that Ω_0 is tuned via exchange bias, in line with theoretical expectations[22]. However, due to the subtle nature of the changes observed, we emphasize that further investigation is required to confirm this mechanism. Considering all dependencies we can reproduce the chevron pattern as demonstrated in Fig. S12d showing a similar tilt as the experimental data. Overall, we find a quantitative understanding of frequency and voltage detuning in Rabi measurements. Additionally, we find experimental fingerprints of the SEC stemming from the exchange bias model.

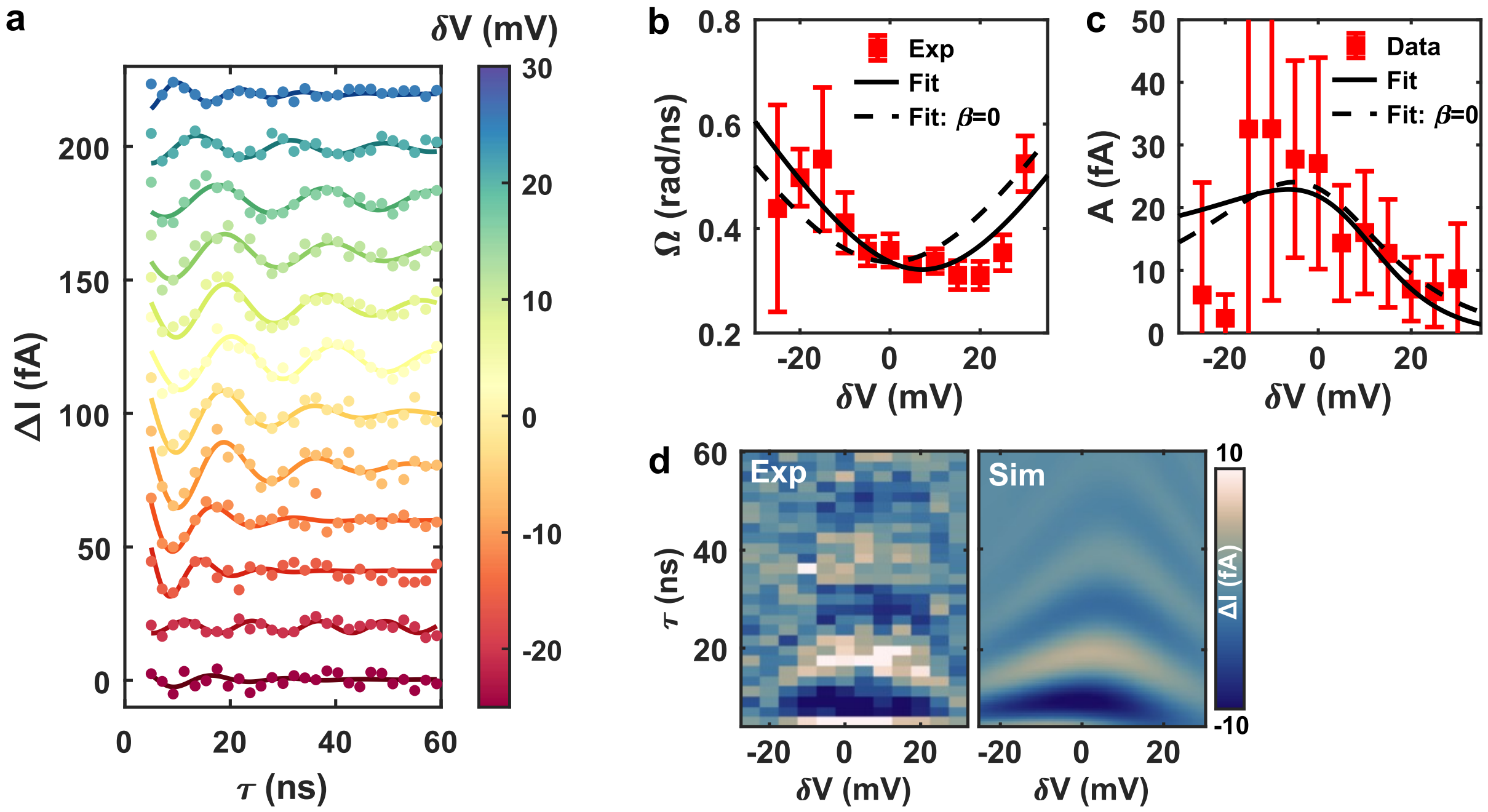

Supplementary Fig. S12. (a) Waterfall plot of the Rabi measurements from Fig. 3c measured at different voltage detuning δV. The solid lines in slightly darker colors as the round datapoints from the experiment show fits to Eq. (3). **(b)** Extracted Rabi rates and error bars from the fits of (a) as a function of δV. The solid line shows a fit to Eq. (S10). **(c)** Extracted amplitudes and error bars from the fits of (a) as a function of δV. The solid line shows a fit to Eq. (S11). **(d)** Left: Colormap of the measurements from (a) showing ΔI as a function of τ and δV. Right: Simulated chevron pattern using Eq. (S10) and (S11).

In this paragraph, we discuss the coherence time of the molecular spins in our experiments. From the Rabi measurements in Fig. 3 and 4, we extract $T_{2,\mathrm{Rabi}}$ times in the order of a few tens of nanoseconds (see Extended Data Tab. 1). These values are comparable to those reported previously for molecules[8,30,31] and Ti atoms[1]. The relatively large error bars originate from the nature of the measurement where $T_{2,\mathrm{Rabi}}$ is obtained from the exponential envelope of the Rabi oscillations Eq. (3). Since only a few oscillations are observable, the exponential envelope is not well resolved. In particular, when detuning from resonance, the Rabi amplitude becomes strongly suppressed, which further deteriorates the precision of the fitted T_2.

As pointed out by Ref.[1] the dominant source of decoherence is voltage induced tunneling current which is needed to read out the spin state. Because V_{DC} also generates the SEC in our experiment, both effects are inseparably linked, and the achievable coherence times are strongly limited by current-induced relaxation. From the experiments shown in Fig. 4 we find the useful range of the SEC provided by V_{DC} limited to ca. $100\ \mathrm{mV}$ which is caused by the required readout current of 1 to $10\ \mathrm{pA}$ and the decoherence from tunneling electrons: For lower currents, the signal becomes too weak and for higher currents the decoherence exceeds the Rabi rate. Therefore, the obtained limitation is not caused by the decoherence from the SEC itself. For a discussion on this, we refer to Section S13. Despite the constraints, the SEC remains sufficiently strong to tune the system between driving the first and the second ESR transition, which constitutes a promising benchmark for future applications. A more sophisticated device architecture, in which SEC and spin readout are provided by separate electrodes would eliminate this constraint as discussed in Section S13.

11. SEC in a Coupled Spin System

The coupled spin system shown in Fig. 4 in the main text can be described by two coupled spin ½ as already demonstrated by earlier works[2,32,33,8]. This leads to the spin Hamiltonian:

$$H = g_{1,z}\mu_B\left(B_z + B_{\mathrm{tip}}\right)S_{1,z} + g_{2,z}\mu_B B_z S_{2,z} + J_{\perp}\left(S_{1,\mathrm{x}}S_{2,\mathrm{x}} + S_{1,\mathrm{y}}S_{2,\mathrm{y}}\right) + J_{\parallel}S_{1,\mathrm{z}}S_{2,\mathrm{z}} \quad \text{(S12)}$$

Where S_1 is the first spin probed by the tip and S_2 is the second spin. We apply an out-of-plane magnetic field B_{z} which determines the quantization axis of the spin. Moreover, in previous ESR-STM measurements on the Fe-FePc complex, we determined $g_{\mathrm{z}} = (2.01\ \pm\ 0.05)$[8]. The coupling between the spins is described by the Heisenberg exchange interaction parallel to the applied magnetic field $J_{\parallel}$ and perpendicular $J_{\perp}$. The eigenstates of the Hamiltonian are given by:

$$|0\rangle = |\uparrow\uparrow\rangle \quad \text{(S13)}$$

$$|1\rangle = |-\rangle = \frac{-\alpha}{\sqrt{1+\alpha^2}}|\uparrow\downarrow\rangle + \frac{1}{\sqrt{1+\alpha^2}}|\downarrow\uparrow\rangle \quad \text{(S14)}$$

$$|2\rangle = |+\rangle = \frac{1}{\sqrt{1+\alpha^2}}|\uparrow\downarrow\rangle + \frac{\alpha}{\sqrt{1+\alpha^2}}|\downarrow\uparrow\rangle \quad \text{(S15)}$$

$$|3\rangle = |\downarrow\downarrow\rangle \quad \text{(S16)}$$

With the relative weight $\alpha = \frac{\delta E + \sqrt{\delta E^2 + J_\perp^2}}{J_\perp}$ and the Zeeman energy difference $\delta E = g_{1,z}\mu_\mathrm{B}(B_\mathrm{z} + B_\mathrm{tip}) - g_{2,z}\mu_\mathrm{B}B_\mathrm{z}$. Therefore, the eigenstates can be tuned by the tip magnetic field. A corresponding energy diagram is displayed in Extended Data Fig. 7a showing how the mixed eigenstates evolve under the influence of the tip field. The simulated ESR signal in Extended Data Fig. 7b shows 4 peaks for $\delta E \to 0$ corresponding to mixed states evolving into a singal with 2 peaks corresponding to Zeeman product states. One finds that at the avoided level crossing the frequency splitting of the two center peaks is given by $\delta f = |J_\perp| - |J_\parallel|$ while the splitting of the two outer peaks $\Delta f = |J_\parallel|$ stays constant.

For the measurements in Fig. 4 the system was operated in the Zeeman state regime described by the energy levels on the left-hand side of Extended Data Fig. 7a. Since in this regime we have a low degree of hybridization between the two spins we can model the Rabi oscillations measured in Fig. 4 well by adding the signal of two individual Chevron patterns.

We note that in principle the dimer of Fe-FePc complexes consists of four spins in total. As shown in Ref.[8] due to the effective S=1/2 ground state, the avoided level crossing can be modelled with both a four and two spin model. We here only introduce the effective two spin model for the sake of simplicity.

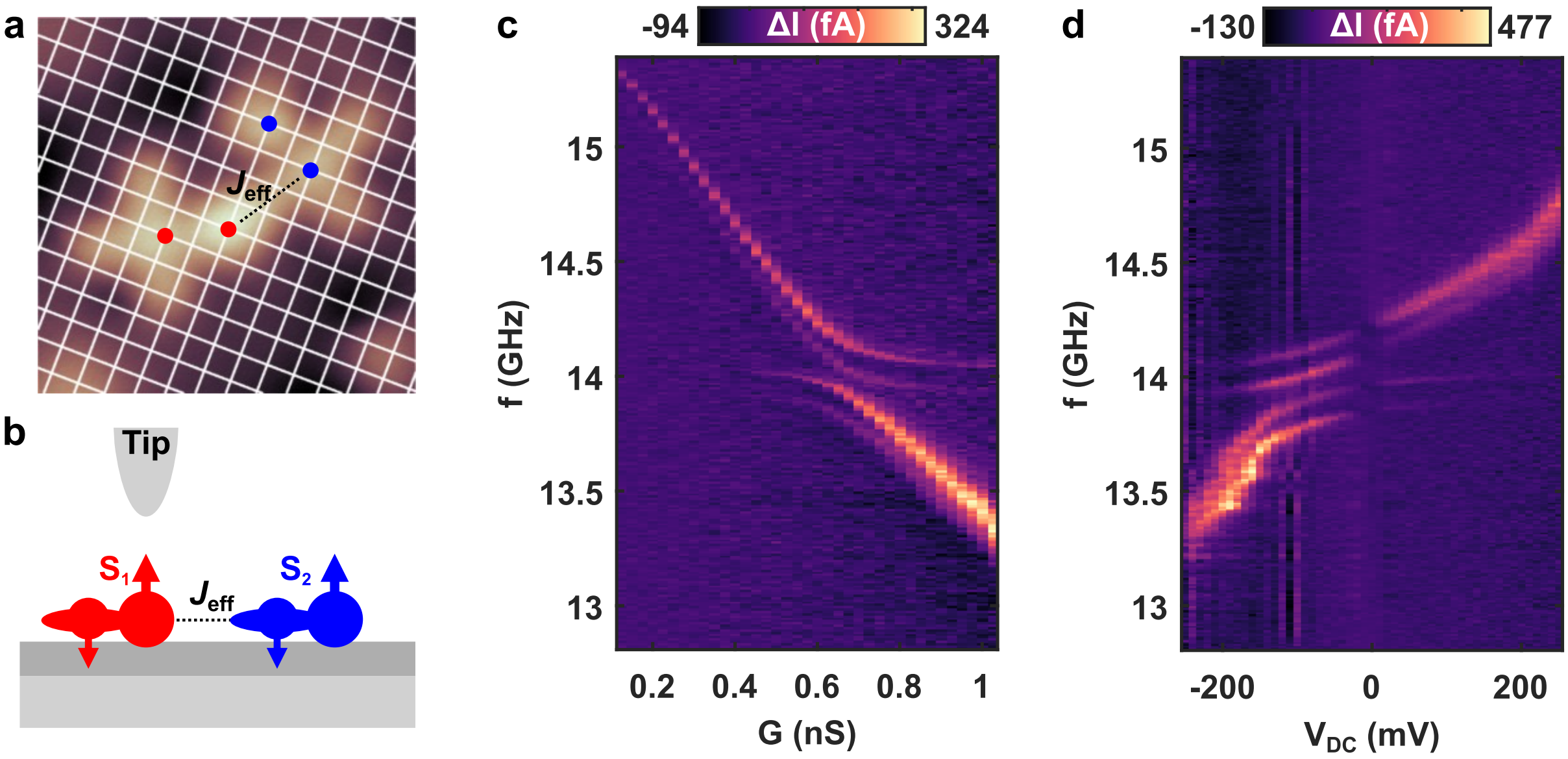

Supplementary Fig. S13. (a) Topography, showing the two coupled Fe-FePc complexes from Fig. 4 (Image conditions: $I = 10\,\mathrm{pA}, V_{\mathrm{DC}} = -100\,\mathrm{mV}$). The inserted white lattice corresponds to the MgO lattice. The colored dots show the respective adsorption sites of the Fe atoms of the first complex (red) and the second complex (blue). **(b)** Illustration of the tunnel junction with the first (red) and second (blue) complex. Both form effective S=1/2 systems which are coupled through an effective exchange interaction in the MHz to GHz range. In the experiment the combined system was addressed by placing the tip above the Fe adatom of the first (red) complex. **(c)** Colormap of ΔI as a function of f and setpoint conductance G of the coupled spin system from Fig. 4 (ESR conditions: $V_{\mathrm{set}} = 40\,\mathrm{mV}, V_{\mathrm{RF}} = 10\,\mathrm{mV}, B = 494\,\mathrm{mT}$) **(d)** Colormap of ΔI as a function of f and V_{DC} (ESR conditions: $I_{\mathrm{set}} = 20\,\mathrm{pA}, V_{\mathrm{set}} = -60\,\mathrm{mV}, V_{\mathrm{RF}} = 10\,\mathrm{mV}, B = 494\,\mathrm{mT}$).

Fig. S13 shows the detuning experiment conducted on the two coupled Fe-FePc complexes presented in Fig. 4. The topography shown in Fig. S13a reveals the lattice site position of all spin centers, where the two left dots in red mark the first complex and the two right dots in blue mark the second complex. The two form S=1/2 units that are coupled with an effective exchange interaction J_{eff} as illustrated in Fig. S13b.

Fig. S13c demonstrates how the coupled spin system can be tuned via the setpoint conductance. Given the SEC we can use V_{DC} at an adjusted setpoint close to the avoided level crossing to obtain the same behavior of the resonance peaks (Fig. S13d). Therefore, the SEC can also provide a fast alternative to tune the eigenstates of a coupled spin system without any movement of the STM tip. Here, our observations match the eigenstate tuning which was reported for single Ti atoms[13]. Notably, this possible tuning of the eigenstates requires the SEC to be localized to the first spin.

In the following section we further evaluate the localization of the SEC. We can use the setpoint dependent measurements to determine the resonance frequency of the first and second spin simultaneously while keeping the tip only above the first spin: For the first spin, it can be extracted directly from the frequency sweeps. It follows a linear dependency on the setpoint conductance G as shown in Fig. S13a. As described above, the Zeeman energies of the two spins are equal at the avoided level crossing. Thus, we can read of the resonance frequency of the second spin directly from this

position as marked by the dotted white lines in Extended Data Fig. 9a. To investigate the SEC, we evaluated this for different bias voltages: $-60\,\mathrm{mV}$, $-40\,\mathrm{mV}$, $-30\,\mathrm{mV}$, $30\,\mathrm{mV}$, $40\,\mathrm{mV}$, $60\,\mathrm{mV}$. Extended Data Fig. 9a shows two examples. Extended Data Fig. 9b shows the combined evaluation where the frequency shift $\Delta f = f_{\mathrm{res}} - 2\mu_{\mathrm{B}}B/h$ is plotted over V_{DC}. While the frequency shift of the first spin (red) changes with V_{DC} and G according to the SEC, the frequency shift of the second spin (blue) remains constant. Therefore, the reported SEC only acts locally on the spin underneath the tip. This localization is in-line with the mechanism of the exchange bias model and is an important demonstration of nanometer scale electric control.

12. Rabi Measurements in a Coupled Spin System

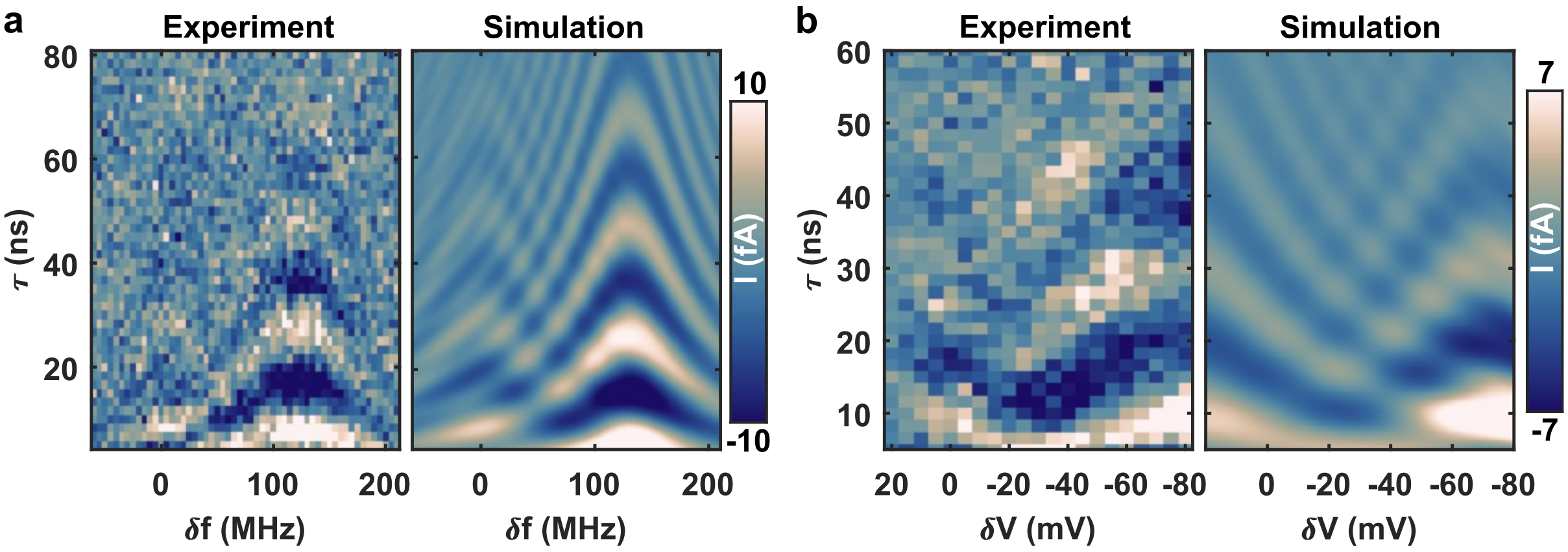

Supplementary Fig. S14. (a) Left: Colormap of Rabi measurements from Fig. 4e plotting ΔI as a function of τ and δf showing a chevron pattern with two arcs. Right: Corresponding simulation using a sum of two chevron patterns with Eq. (3-4) and: $A_1 = 8\,\mathrm{fA}, \Omega_1 = 0.3\,\frac{\mathrm{rad}}{\mathrm{ns}}, \Phi_1 = -150°, T_2^1 = 30\,\mathrm{ns}, A_2 = 20\,\mathrm{fA}, \Omega_2 = 0.3\,\frac{\mathrm{rad}}{\mathrm{ns}}, \Phi_2 = -100°, T_2^1 = 40\,\mathrm{ns}$. **(b)** Left: Colormap of Rabi measurements from Fig. 4f plotting ΔI as a function of τ and δV showing a distorted chevron pattern with two arcs. Right: Corresponding simulation using a sum of two chevron patterns created via Eq. (S10-S11) with two different amplitudes and a voltage dependent decoherence time: $V_{\mathrm{set}}^1 = -20\,\mathrm{mV}, V_{\mathrm{set}}^2 = -120\,\mathrm{mV}, A_1 = 8\,\mathrm{fA}, A_2 = 20\,\mathrm{fA}, \Omega_{00} = 0.3\,\frac{\mathrm{rad}}{\mathrm{ns}}, \alpha = 1.5\,\frac{\mathrm{MHz}}{\mathrm{mV}}, \beta = -0.4\,\frac{\mathrm{MHz}}{\mathrm{mV}}, \Phi = 190°, T_2 = 0.25\,\mathrm{ns/mV} \cdot V_{\mathrm{DC}} + 45\,\mathrm{ns}$.

13. Operation and Implementation Strategies of the Exchange Bias

13.1 Chemical Design Principles for Molecules

The exchange bias formalism allows us to derive general guidelines for the chemical design of molecular systems with strong SEC. In Eq. (2), the strength of the exchange bias is governed by three key parameters: (i) the ionization energy ϵ, (ii) the Coulomb repulsion energy U and (iii) the coupling to the magnetic electrode γ_{T}.

For (i), the SEC is enhanced when the chemical potential of the magnetic electrode approaches one of the two energy levels involved: A strong SEC can be achieved for a small ($\epsilon \approx 0$) or large ($\epsilon \approx -U$) ionization energy, which shifts the exchange bias into the non-linear regime (see e.g. Ext. Data Fig 3d and Eq. S9). For (ii), we utilize that for a given ionization energy (e.g., $\epsilon = -U/2$), the slope of the SEC scales $\propto 1/U$ (Eq. S9) and thus a small Coulomb repulsion energy U is beneficial. From a chemical perspective, (i) and (ii) translate into HOMO ($\epsilon \approx 0$) or LUMO ($\epsilon \approx -U$) states that lie close to the Fermi level as well as a small HOMO-LUMO gap (small U), respectively. We suggest that both can be tuned via e.g. ligand modification, structural alterations, tuning of the spatial extent of the frontier orbitals, or functionalization with electron-donating or electron-withdrawing groups. For (iii), we propose to increase the coupling parameter γ_{T}, that linearly scales with SEC. The exchange bias requires a finite probability for virtual tunneling between the spin-bearing orbital and the magnetic electrode. In this regard molecular geometries and orbitals that lead to a large overlap are favorable (e.g. diffuse s-orbitals or d-orbitals with a z–component such as dz^2 and dxz). Planar systems such as metal phthalocyanines represent convenient examples, but this requirement is not limited to a specific ligand class, as long as the overlap remains finite (typically within ~1 nm). In addition, orbitals with significant spatial extension such as exposed d-orbitals or delocalized radical spins are potentially interesting candidates for a strong SEC.

13.2 Decoherence

In this section, we analyze the relaxation and decoherence introduced by the spin–electric coupling within the transport model and outline design principles for optimizing the SEC mechanism. In the experimental setup of the STM, the coherence of the system is limited by the DC tunneling current[1]. In principle, this could be mitigated by a separate gate electrode (as used in Ref.[20]) that controls the SEC independently from the readout procedure. In that case, the main limitation remains the coupling to the electrodes.

First, we discuss the coherence time T_2 in relation to the frequency shift Δf induced by the exchange field of the magnetic electrode, as described in Eq. (3). According to Ref.[22] Eq. (26), the theoretical limit of T_2 is given by the spin relaxation from sequential tunneling events, $T_2 = 2T_1$. For simplicity, we neglect the coupling to the sample and the driving of the spin, $\gamma_{\mathrm{S}} = A_{\mathrm{T}} = 0$. We find:

$$\frac{1}{T_2} = \frac{1}{h}\gamma_{\mathrm{T}} \cdot \left(\pi - \arctan\left(\frac{eV_{\mathrm{DC}}-\epsilon}{\Delta}\right) + \arctan\left(\frac{eV_{\mathrm{DC}}-\epsilon-U}{\Delta}\right)\right) \quad \text{(S17)}$$

With the energy levels of the SIAM ϵ and $\epsilon + U$ and their broadening $\Delta \ll \mathrm{U}$. In Extended Data Fig. 8b we plot this function over V_{DC} together with the frequency shift

caused by the SEC (Extended Data Fig. 8a). T_2 decreases as V_{DC} approaches one of the energy levels. We find the maximum T_2 when the chemical potential is adjusted in a way that the distance to ϵ and $\epsilon + U$ is equal ($V_{\mathrm{DC}} = \epsilon + \frac{U}{2}$):

$$T_2^{\max} = \frac{U \cdot h}{4\gamma_{\mathrm{T}} \cdot \Delta} \tag{S18}$$

From this expression, it is evident that T_2 is limited by U/Δ. This suggests that designing systems with sharp and well-separated energy levels is advantageous. A more detailed discussion on U will follow below. We note that the level broadening Δ depends on the hybridization with the electrodes and the temperature.

By plotting $\Delta f \cdot T_2(V_{\mathrm{DC}})$ in Extended Data Fig. 8c, an optimal voltage V_{opt} emerges at which the trade-off between frequency shift and decoherence is maximized. In the limit of $\Delta \ll U$ we find analytically $V_{\mathrm{opt}} \approx \epsilon + 0.176\, U$ for negative voltage and $V_{\mathrm{opt}} \approx (\epsilon + U) - 0.176\, U$ for positive voltages. This operating point lies just before the onset of the non-linear SEC regime, where coherence begins to be suppressed by sequential tunneling into the states.

In Extended Data Fig. 8d we plot T_2 over Δf, extracted at different bias voltages, for several tip-molecule couplings γ_{T}. As indicated in Extended Data Fig. 8a,b, T_2 is maximized at $\Delta f = 0$. With increasing Δf, T_2 decreases and eventually saturates at its minimum value once the system enters the non-linear regime of the SEC. Larger γ_{T} values reduce the maximum achievable decoherence time. However, because γ_{T} also enhances the SEC per applied voltage, the decline of T_2 with Δf becomes more gradual. The obtained dependency shows that for a desired Δf we can find an optimal γ_{T} to achieve a maximal decoherence time $T_2(V_{\mathrm{opt}})$. Extended Data Fig. 8e then plots T_2 over Δf at V_{opt} obtained for different γ_{T}. This curve represents the maximal achievable coherence for a given frequency shift when γ_{T} is variable and nicely shows the trade-off between Δf and T_2. We find that one should design the system with weak (strong) γ_{T} when desiring a small (large) SEC, as indicated by the inserted sketches of Extended Data Fig. 8e via the thickness of the tunnel barrier.

Next, we discuss the influence of the Coulomb repulsion U, i.e., the energy separation between the two charge states. Extended Data Fig. 8f shows that increasing U leads to an enhanced coherence time T_2 for all Δf. This improvement is also seen when plotting $\Delta f \cdot T_2$ over the applied voltage V_{DC} (Extended Data Fig. 8g). Here, the maximal point and V_{opt} shift upwards with U, as a larger V_{DC} is required to reach the energy level for SEC. We attribute the improvement with larger U to the underlying mechanism of the SEC: a finite SEC requires an asymmetry in the virtual tunneling probabilities, which is achieved by tuning the chemical potential close to one of the two energy levels. In contrast, decoherence arises from all available tunneling channels and therefore arises from the vicinity to both energy levels. Consequently, increasing U, while adjusting the operating point to V_{opt}, maintains the same SEC strength but reduces decoherence by suppressing tunneling into the energetically more distant level.

The dependence of T_2 and Δf on energy levels highlights useful design principles for quantum-dot architectures or for chemical synthesis strategies. By adjusting the relation between ϵ and $\epsilon + U$ one can naturally tune the system for a maximized coherence $\epsilon = -U/2$ as found in Eq. (S18) or optimal spin-electric coupling $\epsilon = -0.824\ U$ without applying any V_{DC}.

Ultimately, the exchange-mediated SEC will always involve a balance between increased detuning and increased decoherence (Extended Data Fig. 8e). To improve spin coherence within a quantum architecture, an appealing strategy is to implement a spin cascade[34], for example by coupling the electron spin to nuclear spins in the molecule (e.g., ^{57}Fe or ^{15}N). This mirrors the original strategy of the Kane quantum architecture[35], where electrically controlled electron spins mediate interactions between long-lived nuclear spin qubits via the hyperfine coupling.

References:

1. Yang, K. *et al.* Coherent spin manipulation of individual atoms on a surface. *Science* **366**, 509–512 (2019).
2. Zhang, X. *et al.* Electron spin resonance of single iron phthalocyanine molecules and role of their non-localized spins in magnetic interactions. *Nat. Chem.* **14**, 59–65 (2022).
3. Chen, Q. Microscopic Mechanism of Coexisting Electron Spin Resonance and Kondo Resonance in a Single Iron Phthalocyanine Molecule. *Phys. Rev. Lett.* **135**, (2025).
4. Seifert, T. S., Kovarik, S., Gambardella, P. & Stepanow, S. Accurate measurement of atomic magnetic moments by minimizing the tip magnetic field in STM-based electron paramagnetic resonance. *Phys. Rev. Res.* **3**, 043185 (2021).
5. Kumar, M. *et al.* Multireference Theory of Scanning Tunneling Spectroscopy Beyond One-Electron Molecular Orbitals: Can We Image Molecular Orbitals? *J. Am. Chem. Soc.* **147**, 24993–25003 (2025).
6. Qiu, X. H., Nazin, G. V. & Ho, W. Vibronic States in Single Molecule Electron Transport. *Phys. Rev. Lett.* **92**, 206102 (2004).

7. Jones, R. O. & Gunnarsson, O. The density functional formalism, its applications and prospects. *Rev. Mod. Phys.* **61**, 689–746 (1989).
8. Huang, W. *et al.* Quantum spin-engineering in on-surface molecular ferrimagnets. *Nat. Commun.* **16**, 5208 (2025).
9. Wolf, C., Delgado, F., Reina, J. & Lorente, N. Efficient Ab Initio Multiplet Calculations for Magnetic Adatoms on MgO. *J. Phys. Chem. A* **124**, 2318–2327 (2020).
10. Baumann, S. *et al.* Electron paramagnetic resonance of individual atoms on a surface. *Science* **350**, 417–420 (2015).
11. Paul, W. *et al.* Control of the millisecond spin lifetime of an electrically probed atom. *Nat. Phys.* **13**, 403–407 (2017).
12. Reina-Gálvez, J., Wolf, C. & Lorente, N. Many-body nonequilibrium effects in all-electric electron spin resonance. *Phys. Rev. B* **107**, 235404 (2023).
13. Kot, P. *et al.* Electric control of spin transitions at the atomic scale. *Nat. Commun.* **14**, 6612 (2023).
14. Ferrón, A., Rodríguez, S. A., Gómez, S. S., Lado, J. L. & Fernández-Rossier, J. Single spin resonance driven by electric modulation of the g-factor anisotropy. *Phys. Rev. Res.* **1**, 033185 (2019).
15. Willke, P., Yang, K., Bae, Y., Heinrich, A. J. & Lutz, C. P. Magnetic resonance imaging of single atoms on a surface. *Nat. Phys.* **15**, 1005–1010 (2019).
16. König, J. & Martinek, J. Interaction-Driven Spin Precession in Quantum-Dot Spin Valves. *Phys. Rev. Lett.* **90**, 166602 (2003).
17. Martinek, J. *et al.* Kondo Effect in Quantum Dots Coupled to Ferromagnetic Leads. *Phys. Rev. Lett.* **91**, 127203 (2003).

18. Braun, M., König, J. & Martinek, J. Theory of transport through quantum-dot spin valves in the weak-coupling regime. *Phys. Rev. B* **70**, 195345 (2004).

19. Weymann, I., König, J., Martinek, J., Barnas, J. & Schön, G. Tunnel magnetoresistance of quantum dots coupled to ferromagnetic leads in the sequential and cotunneling regimes. *Phys. Rev. B* **72**, 115334 (2005).

20. Hauptmann, J. R., Paaske, J. & Lindelof, P. E. Electric-field-controlled spin reversal in a quantum dot with ferromagnetic contacts. *Nat. Phys.* **4**, 373–376 (2008).

21. Urdaniz, C., Taherpour, S., Yu, J., Reina-Galvez, J. & Wolf, C. Transition-Metal Phthalocyanines as Versatile Building Blocks for Molecular Qubits on Surfaces. *J. Phys. Chem. A* **129**, 2173–2181 (2025).

22. Reina-Gálvez, J., Nachtigall, M., Lorente, N., Martinek, J. & Wolf, C. Contrasting exchange-field and spin-transfer torque driving mechanisms in all-electric electron spin resonance. *Phys. Rev. B* **112**, 245408 (2025).

23. Anderson, P. W. Localized Magnetic States in Metals. *Phys. Rev.* **124**, 41–53 (1961).

24. Ternes, M. Spin excitations and correlations in scanning tunneling spectroscopy. *New J. Phys.* **17**, 063016 (2015).

25. Yu, J., Urdaniz, C., Namgoong, Y. & Wolf, C. On the magnetic bistability of small iron clusters used in scanning tunneling microscopy tip preparation. *New J. Phys.* **25**, 113035 (2023).

26. Yang, K. *et al.* Tuning the Exchange Bias on a Single Atom from 1 mT to 10 T. *Phys. Rev. Lett.* **122**, 227203 (2019).

27. Pearson, K. VII. Note on regression and inheritance in the case of two parents. *Proc. R. Soc. Lond.* **58**, 240–242 (1997).

28. Reina-Galvez, J., Lorente, N., Wolf, C. & Switzer, E. D. Timeesr: An stm-esr code solving a qme in the time domain. (2025).

29. Willke, P. *et al.* Tuning Single-Atom Electron Spin Resonance in a Vector Magnetic Field. *Nano Lett.* **19**, 8201–8206 (2019).

30. Willke, P. *et al.* Coherent Spin Control of Single Molecules on a Surface. *ACS Nano* **15**, 17959–17965 (2021).

31. Kovarik, S. *et al.* Spin torque–driven electron paramagnetic resonance of a single spin in a pentacene molecule. *Science* **384**, 1368–1373 (2024).

32. Yang, K. *et al.* Engineering the Eigenstates of Coupled Spin-$1/2$ Atoms on a Surface. *Phys. Rev. Lett.* **119**, 227206 (2017).

33. Veldman, L. M. *et al.* Free coherent evolution of a coupled atomic spin system initialized by electron scattering. *Science* **372**, 964–968 (2021).

34. Thiele, S. *et al.* Electrically driven nuclear spin resonance in single-molecule magnets. *Science* **344**, 1135–1138 (2014).

35. Kane, B. E. A silicon-based nuclear spin quantum computer. *Nature* **393**, 133–137 (1998).
