# Supplementary material for: Exchange-mediated spin–electric control of single molecules on surfaces
Source: Nat Phys. 2026 Jun 29;22(9):1394–400. doi: 10.1038/s41567-026-03353-w (PMC13558043; doi:10.1038/s41567-026-03353-w)
Supplement: Supplementary file 2 [file 41567_2026_3353_MOESM2_ESM.pdf]

nature portfolio

Peer Review File

Exchange-mediated Spin-Electric Control of Single Molecules on Surfaces

Corresponding Author: Professor Philip Willke

This manuscript has been previously reviewed at another journal. This document only contains information relating to versions considered at Nature Physics.

Version 0:

Reviewer comments:

Reviewer #1

(Remarks to the Author)
The authors observed the linear and nonlinear dependence of external applied voltage in the FePc system via STM probes, demonstrating the significant spin-electric coupling effect of the molecule. Subsequently, they employed this technique to achieve coherent spin control. Through the author's supplementary materials, several intermediate factors have been confirmed or excluded. Currently, there are still a few minor issues that the author is requested to supplement or comment on:
1 The voltage value in mV is consistently used as the unit for measuring spin-electric field coupling. Given that this involves coupling with an external electric field, could the authors supplement the experimental parameters or convert the unit to electric field magnitude?
2 In another study (J. Am. Chem. Soc. 2024,146,25841–25851), researchers similarly observed nonlinear variations in spin crystal field parameters under enhanced external electric fields. Could this phenomenon be related to the behavior described in this paper?

Reviewer #2

(Remarks to the Author)
This resubmission has largely taken my comments into account and the authors have improved their presentation of the chemical background of their system.
Regarding the Zeeman diagram I requested, Figure S19 is more or less what I had in mind. Moreover, the discussion in that section is illuminating on the spin Hamiltonian they use.
Minor error: I believe Equation S12 is missing S2z in its second Zeeman term.
About their expectations of similar behaviour by other molecules, they describe some key physicochemical parameters in section S14. I understand that the manuscript is now submitted to a physics journal, yet still largely concerns a chemical audience. Unfortunately, these physicochemical parameters cannot be easily translated to chemical structures. To put it more plainly, can the authors propose general guidelines as to molecules of potential interest?
Obviously complexes based on pyrrolic ligands (phthalocyanines, porphyrins) are excellent candidates for such studies (flat structures, high thermal stability that allows thermal evaporation, etc). Should we expect such single-molecule techniques to be limited to this family of molecules, or is there a possibility/need/desire for synthetic flexibility?
Once these minor issues are addressed, I am happy to suggest acceptance of the manuscript.

Reviewer #3

(Remarks to the Author)
After carefully perusing the authors' responses to my comments, the replies to the other referees, and the revised version of the manuscript (including the Supplementary Material), I believe the authors have substantially improved the clarity and robustness of the work. The experimental results are convincing, and the interpretation is quite convincing. In the previous round of review for Nature Chemistry, I was positive about the manuscript being considered there once my concerns were addressed, which the authors have now done.

With respect to the present submission to Nature Physics, IMO the work represents an important advance in the electrical

control of molecular spins using ESR-STM, notably by providing an alternative to mechanisms such as piezoelectric displacement or magnetic dipole-dipole interaction. The results will be of clear interest to the surface-science and quantum-spin communities. While the central insights remain closely connected to a specific experimental platform, the demonstrated strength and nonlinearity of the spin–electric coupling, together with the coherent control experiments, may justify consideration in Nature Physics. I therefore leave the final assessment of scope to the editor, noting that the manuscript would also be very well suited for a broad-scope journal such as Nature Communications.

Reviewer #4

(Remarks to the Author)
The authors have adequately addressed my points, which has also strengthened the manuscript. I now recommend publication of the manuscript.

Version 1:

Reviewer comments:

Reviewer #1

(Remarks to the Author)
This work represents an important advance in the electrical control of molecular spins using electron spin resonance–scanning tunneling microscopy (ESR-STM). In particular, it provides a novel alternative to mechanisms. The results will clearly attract considerable attention from researchers in the fields of spin-electric coupling.

Reviewer #2

(Remarks to the Author)
The authors have further improved their manuscript by addressing the additional comments of the reviewers.

I am happy to propose acceptance as is.

Open Access This Peer Review File is licensed under a Creative Commons Attribution 4.0 International License, which permits use, sharing, adaptation, distribution and reproduction in any medium or format, as long as you give appropriate credit to the original author(s) and the source, provide a link to the Creative Commons license, and indicate if changes were made.
In cases where reviewers are anonymous, credit should be given to 'Anonymous Referee' and the source.
The images or other third party material in this Peer Review File are included in the article’s Creative Commons license, unless indicated otherwise in a credit line to the material. If material is not included in the article’s Creative Commons license and your intended use is not permitted by statutory regulation or exceeds the permitted use, you will need to obtain permission directly from the copyright holder.
To view a copy of this license, visit https://creativecommons.org/licenses/by/4.0/

Reviewer comments:

Reviewer #1 (Remarks to the Author):

The authors observed the linear and nonlinear dependence of external applied voltage in the FePc system via STM probes, demonstrating the significant spin-electric coupling effect of the molecule. Subsequently, they employed this technique to achieve coherent spin control. Through the author's supplementary materials, several intermediate factors have been confirmed or excluded. Currently, there are still a few minor issues that the author is requested to supplement or comment on:

1 The voltage value in mV is consistently used as the unit for measuring spin-electric field coupling. Given that this involves coupling with an external electric field, could the authors supplement the experimental parameters or convert the unit to electric field magnitude?

We thank the reviewer for this remark and agree that electric field values are of interest for comparing with other works. In section S6.2 we provide E≈ 0.15 V/nm to 0.75 V/nm for an applied voltage of 50-250 mV using a plate capacitor model E=V/d and d≈333 pm. We present these values specifically in the context of the discussion on the displacement model and not throughout the manuscript, as this could otherwise be misleading: our spin-electric coupling mechanism does not originate from classical electric fields but from the shift in electrochemical potential of the spin-polarized electrode with respect to the molecular spin. That motivated the use of mV, since the applied bias voltage causing the potential shift is the relevant control parameter in our experiment.

2 In another study (J. Am. Chem. Soc. 2024,146,25841–25851), researchers similarly observed nonlinear variations in spin crystal field parameters under enhanced external electric fields. Could this phenomenon be related to the behavior described in this paper?

While we have already cited this work, the reviewer is correct that here a non-linear spin-electric coupling has been found theoretically (Fig. 5). We have overlooked this and now also cite this work when discussing non-linear/ higher-order SEC effects (end of the first paragraph).

We note that the mechanism of the referred work is different as it is based on electric-field-induced structural distortions that modify the crystal field. In our case, the spin–electric coupling emerges from a virtual tunnelling process between the molecule and a spin-polarized electron bath – not relying on structural distortions.

Reviewer #2 (Remarks to the Author):

This resubmission has largely taken my comments into account and the authors have improved their presentation of the chemical background of their system.

Regarding the Zeeman diagram I requested, Figure S19 is more or less what I had in mind. Moreover, the discussion in that section is illuminating on the spin Hamiltonian they use.

Minor error: I believe Equation S12 is missing S2z in its second Zeeman term.

About their expectations of similar behaviour by other molecules, they describe some key physicochemical parameters in section S14. I understand that the manuscript is now submitted to a physics journal, yet still largely concerns a chemical audience. Unfortunately, these physicochemical parameters cannot be easily translated to chemical structures. To put it more plainly, can the authors propose general guidelines as to molecules of potential interest?

Obviously complexes based on pyrrolic ligands (phthalocyanines, porphyrins) are excellent candidates for such studies (flat structures, high thermal stability that allows thermal evaporation, etc). Should we expect such single-molecule techniques to be limited to this family of molecules, or is there a possibility/need/desire for synthetic flexibility?

Once these minor issues are addressed, I am happy to suggest acceptance of the manuscript.

On equation S12: We thank the reviewer for the careful read. Indeed, equation S12 was missing the spin operator of the second spin S2z. We corrected the formula in the revised version of the supplement.

On section S14: We agree with the reviewer that addressing the design principles for molecules is of interest, and that this can be further optimized by chemistry. In section S13 (former S14) we now added a general discussion on chemical design principles. While our expertise in organic synthesis is limited, the SEC according to Eq. (2) in the main text clearly translates into molecular design criteria, which in short are:

a. small ionization energy $\epsilon \sim 0$ (shallow HOMO) or large ionization energy $\epsilon \sim -U$ (low lying LUMO)
b. a small Coulomb repulsion U (small HOMO-LUMO gap)
c. strong coupling with the magnetic electrode γ_T

Concerning the last point, phthalocyanines and porphyrins are somewhat beneficial due to their flat structure, but the underlying exchange bias mechanism applies in general to all molecules. Indeed, we believe that there is substantial room for improvement via synthetic chemistry for all three factors.

We have attached the new subsection (S13.1) of former Section S14 (now Section S13) for convenience below:

"The exchange bias formalism allows us to derive general guidelines for the chemical design of molecular systems with strong SEC. In Eq. (2), the strength of the exchange bias is governed by three key parameters: (i) the ionization energy ϵ, (ii) the Coulomb repulsion energy U and (iii) the coupling to the magnetic electrode γ_T.

For (i), the SEC is enhanced when the chemical potential of the magnetic electrode approaches one of the two energy levels involved: A strong SEC can be achieved for a small (ϵ≈0) or large (ϵ≈-U) ionization energy, which shifts the exchange bias into the non-linear regime (see e.g. Ext. Data Fig 3d and Eq. S9). For (ii), we utilize that for a given ionization energy (e.g., ϵ=-U/2), the slope of the SEC scales ∝1/U (Eq. S9) and thus a small Coulomb repulsion energy U is beneficial. From a chemical perspective, (i) and (ii) translate into HOMO (ϵ≈0) or LUMO (ϵ≈-U) states that lie close to the Fermi level as well as a small HOMO-LUMO gap (small U), respectively. We suggest that both can be tuned via e.g. ligand modification, structural alterations, tuning of the spatial extent of the frontier orbitals, or functionalization with electron-donating or electron-withdrawing groups. For (iii), we propose to increase the coupling parameter γ_T, that linearly scales with SEC. The exchange bias requires a finite probability for virtual tunneling between the spin-bearing orbital and the magnetic electrode. In this regard molecular geometries and orbitals that lead to a large overlap are favorable (e.g. diffuse s-orbitals or d-orbitals with a z–component such as d_(z^2) and d_xz). Planar systems such as metal phthalocyanines represent convenient examples, but this requirement is not limited to a specific ligand class, as long as the overlap remains finite (typically within ~1 nm). In addition, orbitals with significant spatial extension such as exposed d-orbitals or delocalized radical spins are potentially interesting candidates for a strong SEC."

We thank the reviewer for the remark, which we believe has strengthened the message of the paper

Reviewer #3 (Remarks to the Author):

After carefully perusing the authors' responses to my comments, the replies to the other referees, and the revised version of the manuscript (including the Supplementary Material), I believe the authors have substantially improved the clarity and robustness of the work. The experimental results are convincing, and the interpretation is quite convincing. In the previous round of review for Nature Chemistry, I was positive about the manuscript being considered there once my concerns were addressed, which the authors have now done.

With respect to the present submission to Nature Physics, IMO the work represents an important advance in the electrical control of molecular spins using ESR-STM, notably by providing an alternative to mechanisms such as piezoelectric

displacement or magnetic dipole-dipole interaction. The results will be of clear interest to the surface-science and quantum-spin communities. While the central insights remain closely connected to a specific experimental platform, the demonstrated strength and nonlinearity of the spin–electric coupling, together with the coherent control experiments, may justify consideration in Nature Physics. I therefore leave the final assessment of scope to the editor, noting that the manuscript would also be very well suited for a broad-scope journal such as Nature Communications.

We thank the reviewer for the support. We highlight again, that while the SEC has been implemented in the present case inside an STM tunnel junction, the exchange bias mechanism in itself is universal and can be readily translated to other spin qubit platforms such as quantum dots and solid state spin defects.

Reviewer #4 (Remarks to the Author):

The authors have adequately addressed my points, which has also strengthened the manuscript. I now recommend publication of the manuscript.

We thank the reviewer for the support.
